# Supplementary material for: DUSP6 inhibition overcomes neuregulin/HER3-driven therapy tolerance in HER2+ breast cancer
Source: EMBO Mol Med. 2024 Jun 17;16(7):8. doi: 10.1038/s44321-024-00088-0 (PMC11251193; doi:10.1038/s44321-024-00088-0)
Supplement: Supplementary file 1 — Table EV1 [file 44321_2024_88_MOESM1_ESM.docx]

**Expanded view table 1:**

**Used siRNA sequences**

| Target Gene | Cat number | Company | Concentration |
| --- | --- | --- | --- |
| *HER2* | s613 | ThermoFisher | 20 nM |
| *HER3* | SI02660245 | Qiagen | 100 nM |
| *AKT1* | s659 | ThermoFisher | 20 nM |
| *DUSP1* | SI03100048 | Qiagen | 150 nM |
| *DUSP6* | SI03106404  s4379  s4378 | Qiagen  ThermoFisher  ThermoFisher | 150 nM |
| Negative controls | SI03650318  4390844  4390847 | Qiagen  ThermoFisher  ThermoFisher | - |
