# Supplementary material for: DUSP6 inhibition overcomes neuregulin/HER3-driven therapy tolerance in HER2+ breast cancer
Source: EMBO Mol Med. 2024 Jun 17;16(7):8. doi: 10.1038/s44321-024-00088-0 (PMC11251193; doi:10.1038/s44321-024-00088-0)
Supplement: Supplementary file 2 — Table EV2 [file 44321_2024_88_MOESM2_ESM.docx]

**Expanded view Table 2:**

**Antibodies**

| Target protein | Cat number | Company | Clone | Dilution |
| --- | --- | --- | --- | --- |
| HER2 | sc-33684 | Santa Cruz | 3B5 | 1:500-1:5000 |
| p-HER2 | 2243 | CST | 6B12 | 1:1000 |
| HER3 | 12708  05-390 | CST  Millipore | D22C5  2F12 | 1:1000  1:500 |
| p-HER3 | 4791 | CST | 21D3 | 1:1000 |
| DUSP1 | 07-535 | Millipore | - | 1:500 |
| DUSP6 | ab76310 | Abcam | EPR129Y | 1:1000 |
| AKT | sc-5298 | Santa Cruz | B-1 | 1:500 |
| p-AKT | 4060 | CST | D9E | 1:1000 |
| ERK1/2 | sc-514302 | Santa Cruz | C-9 | 1;1000 |
| p-ERK1/2 | 9101 | CST | - | 1:1000 |
| β-actin | sc-47778 | Santa Cruz | C4 | 1:5000 |
| P38 | 8690 | CST | D13E1 | 1:1000 |
| p-P38 | 4511 | CST | D3F9 | 1:1000 |
| JNK1/2 | 3708 | CST | 2C6 | 1:1000 |
| p-JNK1/2 | 4668 | CST | 81E11 | 1:1000 |
| PARP-1 | 9532 | CST | 46D11 | 1:1000 |
| Cl-PARP-1 | 5625  ab32064 | CST  Abcam | D64E10  E51 | 1:1000  1:2500 |
| GAPDH | 5G4-6C5 | HyTest | - | 1:5000 |

CST: Cell signaling technology.
