# Supplementary material for: DUSP6 inhibition overcomes neuregulin/HER3-driven therapy tolerance in HER2+ breast cancer
Source: EMBO Mol Med. 2024 Jun 17;16(7):8. doi: 10.1038/s44321-024-00088-0 (PMC11251193; doi:10.1038/s44321-024-00088-0)
Supplement: Supplementary file 3 — Table EV3 [file 44321_2024_88_MOESM3_ESM.docx]

**Expanded view Table 3**

**qRT-PCR primers**

| Gene | Accession number | Forward primer (5’→3’) | Reverse primer (5’→3’) | Size (bp) |
| --- | --- | --- | --- | --- |
| *DUSP1* | NM_004417 | AGGCCATTGACTTCATAGACTCC | TGGGAGAGATGATGCTTCGC | 177 |
| *DUSP6* | NM_001946 | CCTGGAAGGTGGCTTCAGTAA | GCACTATTGGGGTCTCGGTC | 180 |
| *B2M* | NM_004048 | GATGAGTATGCCTGCCGTGT | CTGCTTACATGTCTCGATCCCA | 79 |
| *HER3* | NM_001982 | GGTGATGGGGAACCTTGAGAT | CTGTCACTTCTCGAATCCACTG | 80 |
| *HER2* | NM_004448 | TGTGACTGCCTGTCCCTACAA | CCAGACCATAGCACACTCGG | 152 |
| *BIRC5* | NM_001168 | CCAGATGACGACCCCATAGAG | TTGTTGGTTTCCTTTGCAATTTT | 152 |
| *E2F1* | NM_005225 | TGGACTCTTCGGAGAACTTTCA | TGATCCCACCTACGGTCTCC | 93 |
| *CCNE2* | NM_057749 | TAGCTGGTCTGGCGAGGTTT | GGCCTGGATTATCTGGGCTT | 130 |
| *CDC6* | NM_001254 | GCGAGGCCTGAGCTGTG | AGGCAGGGCTTTTACACGAG | 181 |
| *CENPF* | NM_016343 | CGTCCCCGAGAGCAAGTTTA | GTAGGCAGCCCTTCTTTCCA | 98 |
| *NCAPG* | NM_022346 | AGTCCACATAGAGAAGAATGATGC | TCCACAGCATCCCAAGCATA | 199 |
| *CDK1* | NM_001786 | AAACTACAGGTCAAGTGGTAGCC | TCCTGCATAAGCACATCCTGA | 148 |
| *TYMS* | NM_001071 | CTGCTGACAACCAAACGTGTG | GCATCCCAGATTTTCACTCCCTT | 116 |
| *RAD51* | NM_133487 | CAACCCATTTCACGGTTAGAGC | TTCTTTGGCGCATAGGCAACA | 107 |
| *PCNA* | NM_002592 | GCGTGAACCTCACCAGTATGT | TCTTCGGCCCTTAGTGTAATGAT | 76 |
| *RRM1* | NM_001033 | GCCAGGATCGCTGTCTCTAAC | GAGAGTGTTTGCCATTATGTGGA | 106 |
| *RFC3* | NM_002915 | GTGGACAAGTATCGGCCCTG | TGATGGTCCGTACACTAACAGAT | 120 |
| *ESCO2* | NM_001017420 | TGTGTGCAAGTCTTGTGGTATG | CCCATCCCAAAACTCTGCTACT | 145 |
| *FOXM1* | NM_202002 | ATAGCAAGCGAGTCCGCATT | AGCAGCACTGATAAACAAAGAAAGA | 151 |
| *CDC25A* | NM_001789 | CTACCTCAGAAGCTGTTGGGA | AAAGGCTTCATTTTCCTTGTTCTCA | 117 |
| *AURKB* | NM_001313950 | ACCTGCACCATCCCAACATC | TGCCAACTCCTCCATGATCG | 164 |
| *PLK1* | NM_005030 | TCTTCCAGGATCACACCAAGC | AGGAGACTCAGGCGGTATGT | 100 |
| *CCNB1* | NM_031966 | GATACTGCCTCTCCAAGCCC | TGACTGCTTGCTCTTCCTCAA | 196 |
| *CDC25C* | NM_001287582 | CTACCCAGTCGGAAGGCAGA | AAACAAAACCTAGCTCAAGCCT | 159 |
| *AURKA* | NM_001323303 | CTCAGTGGCGGACGAGGA | GGAGTGAGACCCTCTAGCTGT | 200 |
| *SYNJ1* | NM_003895 | AGCTCTTGAAGGGAAAGCGAA | TGTCTGCTCAGAAACACGCAA | 193 |
|  |  |  |  |  |
