# Supplementary material for: DUSP6 inhibition overcomes neuregulin/HER3-driven therapy tolerance in HER2+ breast cancer
Source: EMBO Mol Med. 2024 Jun 17;16(7):8. doi: 10.1038/s44321-024-00088-0 (PMC11251193; doi:10.1038/s44321-024-00088-0)
Supplement: Supplementary file 4 — Appendix [file 44321_2024_88_MOESM4_ESM.pdf]

Appendix

Momeny et al.,
DUSP6 inhibition overcomes Neuregulin/HER3-driven therapy tolerance in HER2+ breast cancer

Table of content:

Appendix Figure S1 (page 2)

Appendix Figure S2 (page 3)

Appendix Figure S3 (page 4)

Appendix Figure S4 (page 5)

Appendix Figure S5 (page 6)

Appendix Figure S6 (page 7)

Appendix Figure S7 (page 8)

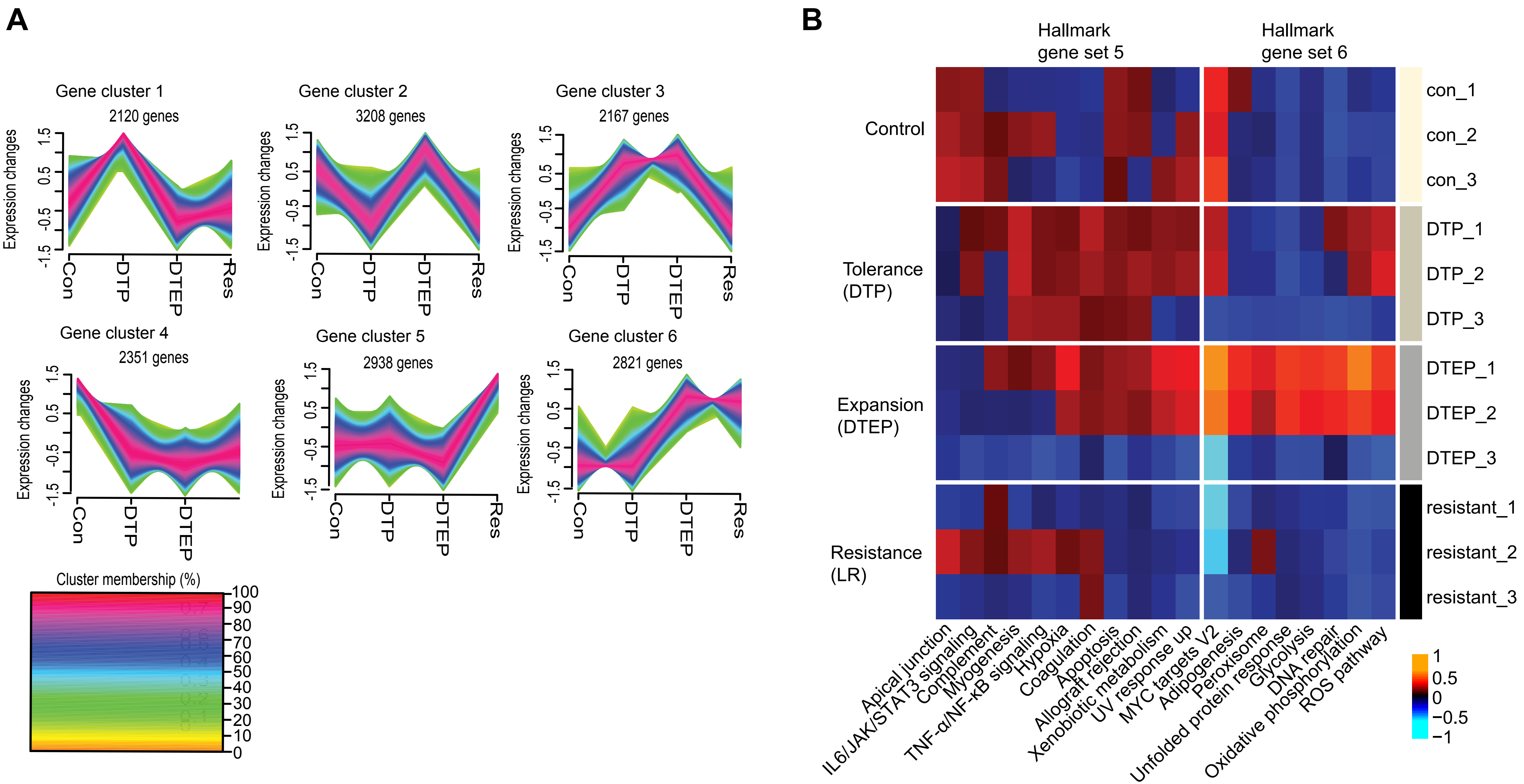

Appendix Figure S1 (A) Genes with similar patterns of expression changes during the lapatinib resistance development were clustered by unsupervised soft clustering analysis (see Table EV2 for genes included in each cluster). The Cluster membership color coding indicates how tightly the genes are associated with the overall pattern of gene regulation across development of lapatinib resistance.
(B) Continued from Figure 1C: Differentially expressed pathways were identified using the R package limma and hallmark gene sets were used for GSVA analysis to reveal hallmarks and signal transduction pathways involved in each step of the resistance acquisition.

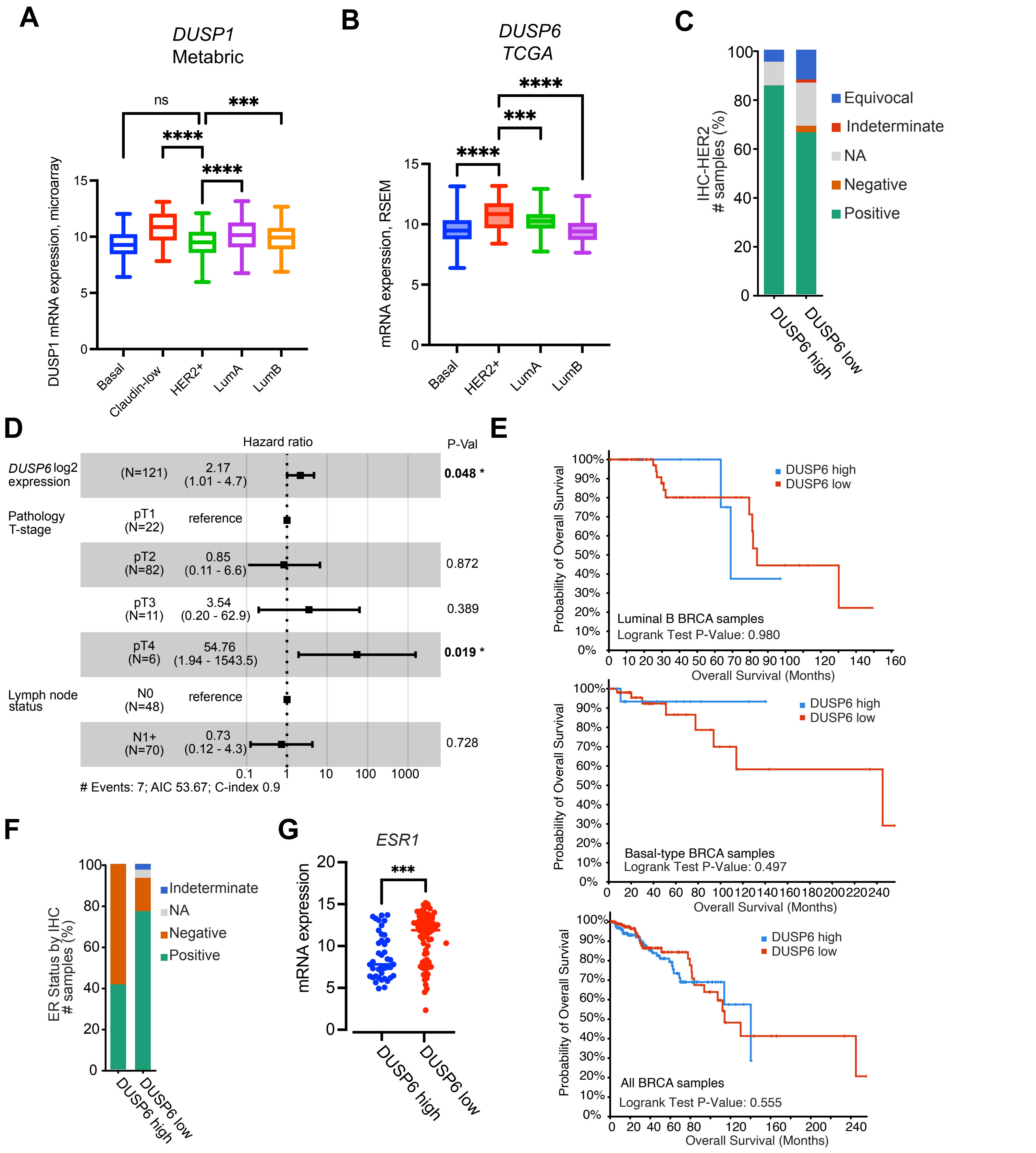

Appendix Figure S2

(A) DUSP1 is not overexpressed in HER2+ breast cancer over other breast cancer subtypes. Data were extracted from the METABRIC dataset and categorized into five molecular subtypes according to the PAM50 gene expression subtype classification (basal, claudin-low, HER2+, Luminal A, and Luminal B). Data were analyzed by one-way ANOVA followed by Tukey's multiple comparisons test. Statistically significant values of ***p < 0.001 and ****p < 0.0001 were determined. (B) DUSP6 overexpression in HER2+ breast cancer in the TCGA breast invasive carcinoma dataset. Data were categorized into 4 molecular subtypes including basal (171 patients), HER2+ (78 pts), Luminal A (499 pts), and Luminal B (197 pts). Data were analyzed by one-way ANOVA followed by Tukey's multiple comparisons test. Statistically significant values of ***p < 0.001 and ****p < 0.0001 were determined. (C) Breast cancer patients from the TCGA Firehose legacy dataset were divided into DUSP6 high (LogFC>1, FDR<0.05) and low expression (LogFC<-1, FDR<0.05) groups and IHC staining positivity for HER2 was compared between the two groups. (D) Breast cancer patients with HER2+ from the TCGA-BRCA were included with relapse free survival modelling in multivariable Cox regression, with predictors being DUSP6 expression in log2-scale, pathological T-stage, and lymph node status. (E) Breast cancer patients from the TCGA-BRCA dataset were divided into DUSP6 high (LogFC>1, FDR<0.05) and low expression (LogFC<-1, FDR<0.05) groups and the clinical attributes including the overall survival were compared between the two groups in each of the indicated breast cancer (BRCA) subtype (Log-rank Test p value=0.555). (F, G) ERBB2 high-expressing (LogFC>1, FDR<0.05) breast cancer patients from the TCGA Firehose legacy dataset were subdivided into DUSP6high and low groups and IHC positivity for ER (F) and the mRNA levels of ESR1 (G) were compared between the two groups. Data were analyzed by two-tailed t test; ***p < 0.001 (DUSP6high=41, DUSP6low=74).

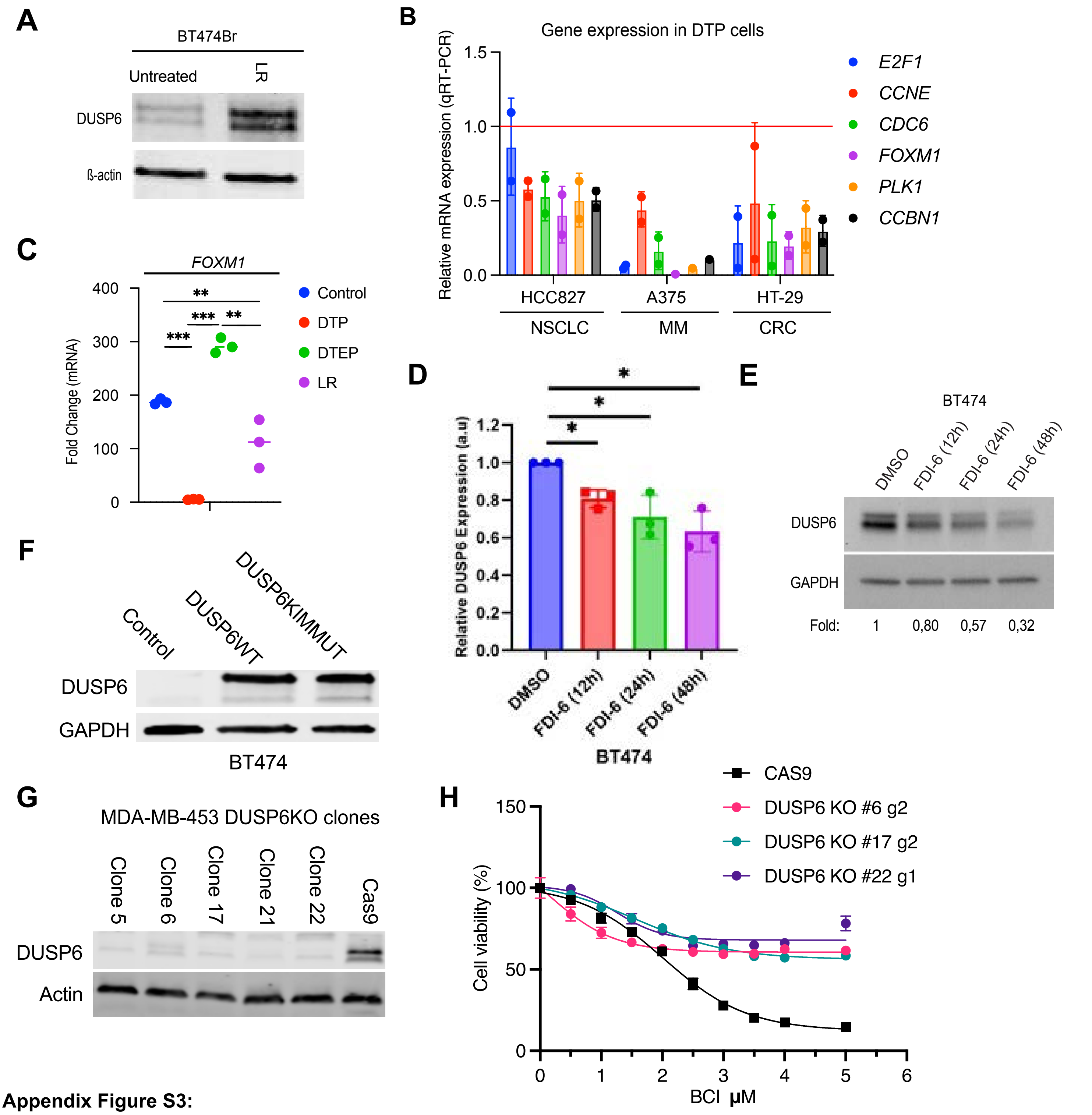

Appendix Figure S3:
(A) Increased expression of DUSP6 in LR of BT474Br cells by Western blot analysis. (B) qRT-PCR analysis of the mRNA expression of the indicated genes in cell lines treated for 10 days as follows: A375 (human epithelial BRAF V600E malignant melanoma; MM) 1 µM dabrafenib + 100 nM trametinib, HT-29 (BRAF V600E colorectal cancer; CRC) 1 µM dabrafenib + 10 µg/ml cetuximab, HCC827 (EGFR-mutant NSCLC) 1 µM osimertinib. Shown is the mean + SD from two experiments. (C) Changes in the FOXM1 mRNA levels during the acquisition of lapatinib resistance in BT474 cells. Data is based on RNA sequencing analysis (Dataset EV1) and was analyzed by one-way ANOVA followed by Tukey's multiple comparisons test. Statistically significant values of ***$p < 0.001$ and ****$p < 0.0001$ were determined. (D) qRT-PCR analysis of DUSP6 mRNA expression in BT474 cells treated with the FOXM1 inhibitor FDI-6 (20 µM) for 12h, 24h, and 48h respectively. Shown is the mean + SD from three independent experiments analysed by student's t-test with the statistically significant value of *$p < 0.05$.(E) Western blot analysis of DUSP6 expression in BT-474 cells treated with the FOXM1 inhibitor FDI-6 for the indicated periods of time. Shown is a representative data from three biological replicates with identical results. Fold change indicates expression of DUSP6 normalized to GAPDH in each sample. (F) Western blot analysis of DUSP6 protein expression in BT474 cells transduced with either wild-type DUSP6 (DUSPWT) or KIM mutant (DUSP6KIMMUT). GAPDH was used as a loading control. (G) CRISPR-mediated knockout of DUSP6 in MDA-MB-453 cells. DUSP6 protein expression of five independent DUSP6 KO single cell clones and CAS9 control cells by Western blot analysis. ß-actin was used as a loading control. (H) DUSP6 knockout decreases sensitivity to BCI in MDA-MB-453 cells. MDA-MB-453 CAS9 control cells and three independent DUSP6 KO single cell clones were treated with the increasing concentrations of BCI for 48 h and cell viability was measured using WST-1 assay. Shown is data from four technical replicate samples from a representative of three experiments with similar results. Data is presented as mean and ± standard deviation (SD).

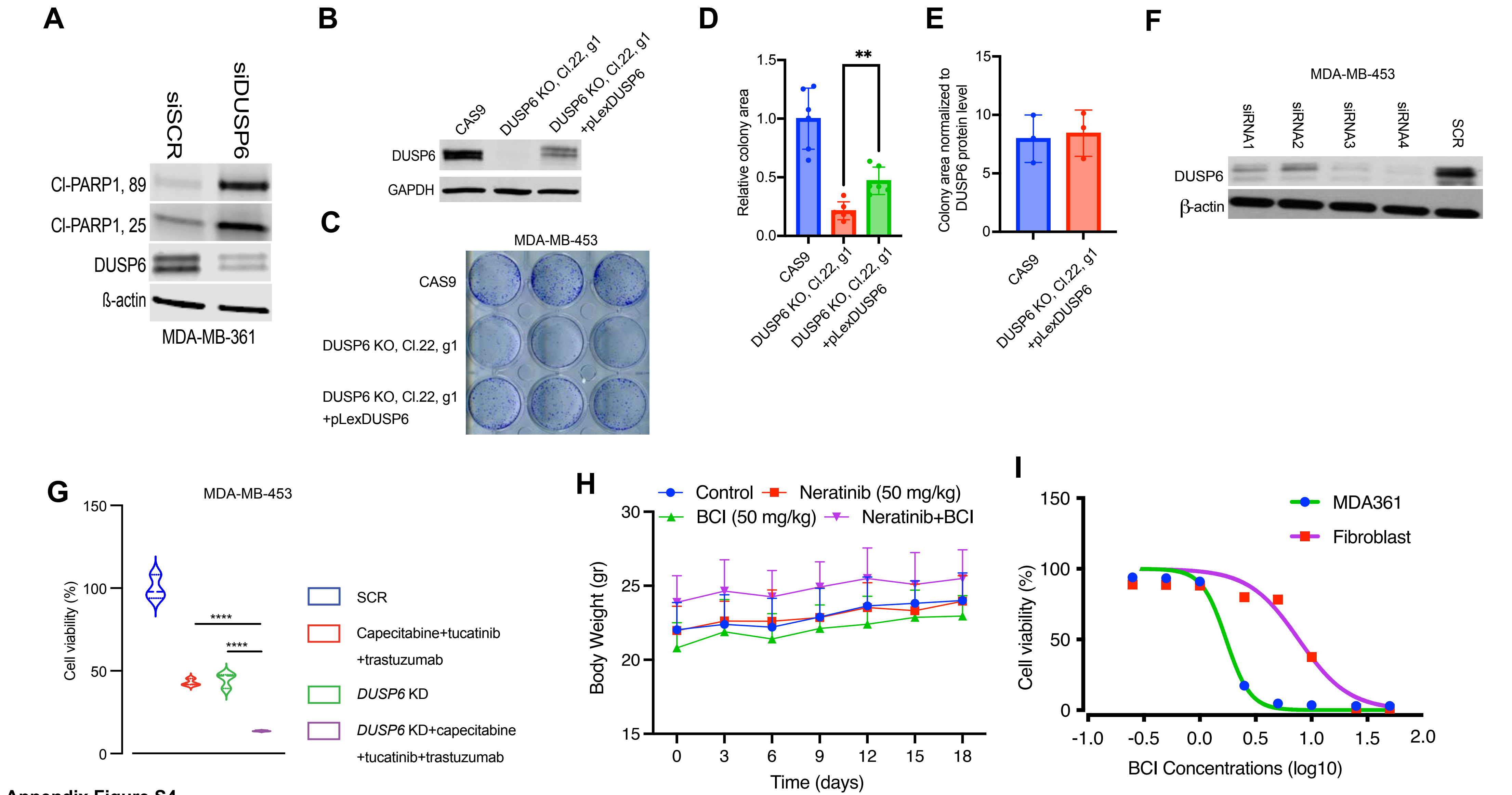

Appendix Figure S4

(A) RNAi-mediated DUSP6 knockdown induces apoptotic cell death in MDA-MB-453 cells, as shown by PARP-1 cleavage. (B) Western blot analysis of DUSP6 protein expression in MDA-MB-453 CAS9 control, DUSP6 KO, and DUSP6 rescue cells. GAPDH was used as a loading control. (C) DUSP6 expression rescues colony growth in DUSP6KO MDA-MB-453 cells. The MDA-MB-453 CAS9 control, DUSP6 KO, and DUSP6 rescue cells were seeded at low density and maintained for 10 d. The colonies were stained with 0.5% crystal violet and imaged using an Epson scanner. (D) The relative colony area of DUSP6 KO and DUSP6 rescue cells normalized to MDA-MB-453 CAS9 control cells. Statistical analysis was performed using an unpaired two-tailed t-test. (n=6)(E) The percentage of area covered by cell colonies normalized to relative DUSP6 protein levels in MDA-MB-453 CAS9 control and DUSP6 rescue cells (n=3). (F) Optimization of DUSP6 knockdown by 4 different siRNAs. The cultures were transfected with 150 nM of the DUSP6-targeting and the negative control siRNA for 3 d, followed by Western blot analysis for DUSP6. (G) DUSP6 siRNA knockdown increases sensitivity of HER2i resistant MDA-MB-453 cells to combination with capecitabine, trastuzumab and tucatinib. Cell viability was measured by WST-1 assay after 48 h of drug treatment. Data were collected from three independent experiments each performed in triplicate and analyzed by one-way ANOVA followed by Tukey's multiple comparisons test. Statistically significant values of ****$p < 0.0001$ were determined. (H) The impact of BCI in combination with neratinib on mice body weight in xenograft models of HCC1954. Mice with tumor size 100 mm3 were randomized into the experimental and the control groups and the body weights were measured every 3 d. (I) Comparison of the anti-proliferative effects of BCI on MDA-MB-361 and an immortalized murine fibroblast cell line. The cells were treated with the increasing concentrations of the drug for 48h and cell viability was measured by WST-1 assay.

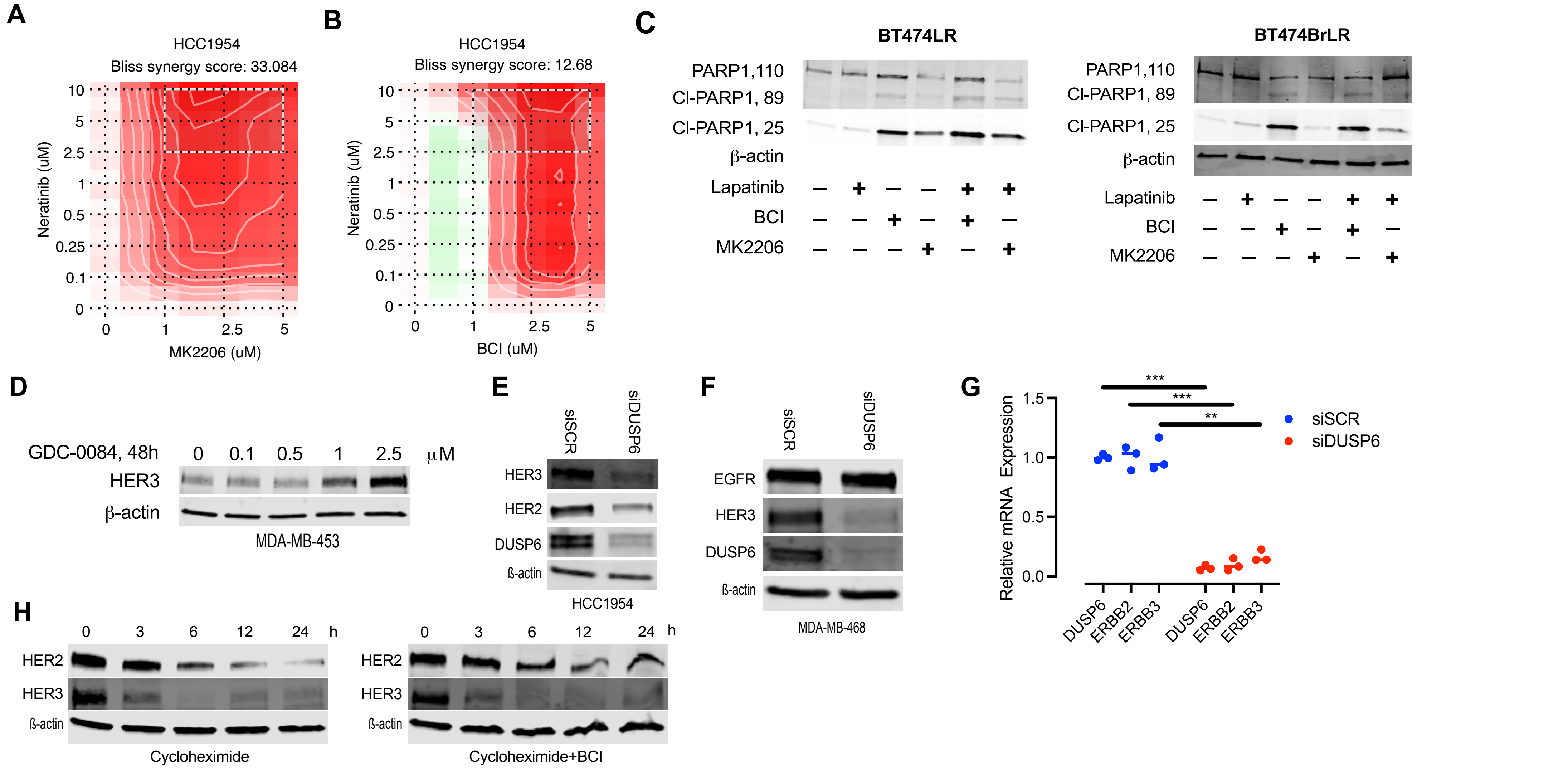

Appendix Figure S5 (A,B) A 2D synergy map of neratinib+MKK2206 (A) and neratinib+BCI (B) combination in HCC1954 cells calculated by Bliss SynergyFinder. The cultures were treated with increasing concentrations of the compounds for 48 h and cell viability was measured by WST-1 assay. (B) Comparison of the effects of lapatinib+BCI and lapatinib+MK2206 on cleaved PARP-1 protein levels by Western blot analysis in BT474LR and BT474BrLR cells. The cells were treated with lapatinib (1 μM), MK2206 (2.5 μM) and BCI (2.5 μM) for 48 h. (C) The dose-dependent effects of the dual PI3K/mTOR inhibitor paxalisib (GDC-0084) on HER3 levels, as indicated by Western blot analysis. (D, E) DUSP6 knockdown inhibits HER2 and HER3 expression in HCC1954 and HER3 protein in MDA-MB-468 cells, respectively, as shown by Western blot analysis. (F) DUSP6 knockdown decreases the mRNA levels of both ERBB2 and ERBB3 in MDA-MB-453 cells, as measured by qRT-PCR. The cells were transfected with the DUSP6-targeting and the negative control siRNA for 3 d, followed by RNA harvest and qRT-PCR analysis for DUSP6, ERBB2 and ERBB3. Data were analyzed by two-way ANOVA followed by Šídák's multiple comparisons test. Statistically significant values of ** p < 0.01 and ***p < 0.001 were determined. (G) BCI has no effect on HER2 and HER3 protein stability, as shown by the cycloheximide chase assay. MDA-MB-453 cells were treated with BCI (2.5 μM) and cycloheximide (50 μg/mL) in a time-dependent manner and the expression of HER2 and HER3 was compared with a cycloheximide-treated group.

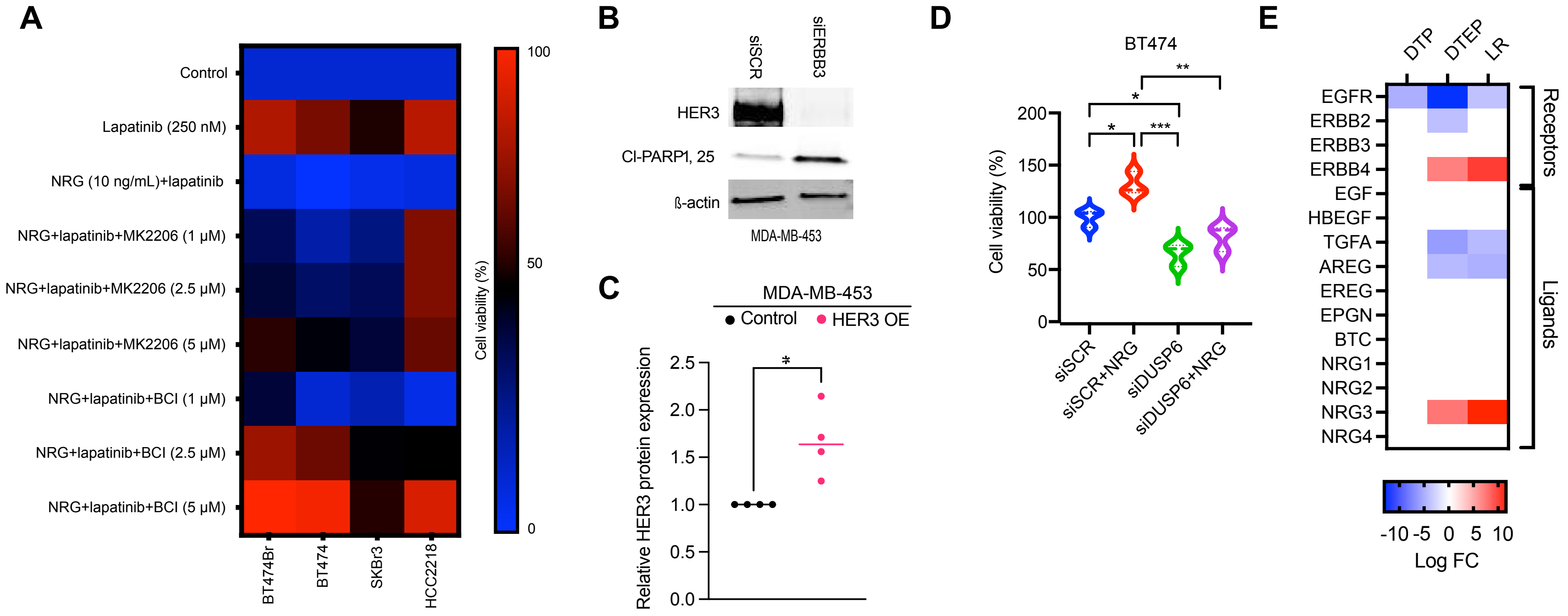

Appendix Figure S6

(A) Comparison of the effects of lapatinib+BCI and lapatinib+MK2206 on NRG-mediated rescue from the anti-proliferative activity of lapatinib. The cells were treated with NRG (10 ng/mL), lapatinib (250 nM), MK2206 (1, 2.5 and 5 μM) and BCI (1, 2.5 and 5 μM) for 48 h and cell viability was measured by WST-1 assay. (B) ERRB3 knockdown by RNAi induces apoptosis in MDA-MB-453 cells, as shown by Western blotting for cleaved PARP-1 protein. (C) Expression of HER3 protein in either control MDA-MB-453 cells or stable clones in which HER3 was overexpressed (OE) by lentivirus. Shown is quantitated data from four independent Western blot analysis. *p < 0.05 by Welch´s t-test. (D) DUSP6 knockdown by RNAi prevents NRG-elicited induction in cell viability in BT474 cells after 2 d of NRG (10 ng/mL) treatment. Data were analyzed by one-way ANOVA followed by Tukey's multiple comparisons test. Statistically significant values of *p < 0.05, ** p < 0.01 and ***p < 0.001 were determined. (E) Changes in the mRNA levels of the EGFR family of receptors and ligands during the acquisition of lapatinib resistance in BT474 cells. Data was obtained from RNA-seq. analysis from cells in Fig. 1A.

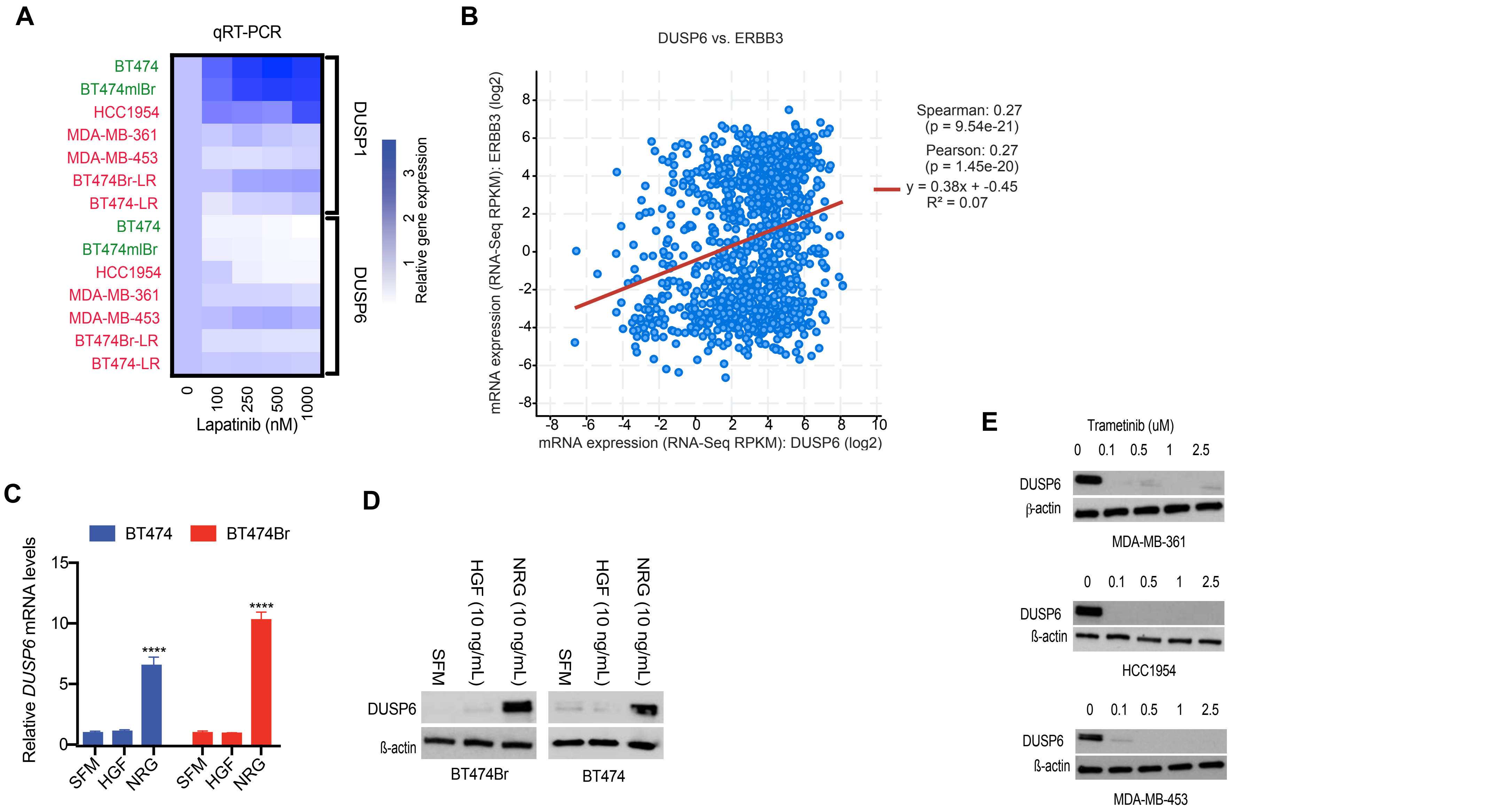

Appendix Figure S7 (A) The effects of lapatinib on the mRNA levels of DUSP1 and DUSP6 in HER2+ breast cancer cell lines. The cells were treated with the increasing concentrations of lapatinib for 48 h, followed by RNA harvest and qRT-PCR analysis. (B) A significant association between the mRNA levels of DUSP6 and ERBB3 in the TCGA breast invasive carcinoma dataset. (C)The effects of NRG on USP6 mRNA levels in BT474 and BT474Br cells. The cultures were serum-starved for 24 h, then treated with NRG (10 ng/mL) or HGF (10 ng/mL) for 48 h, followed by RNA harvest and qRT-PCR analysis. Data were analyzed by one-way ANOVA followed by Dunnett's multiple comparisons test. Statistically significant values ****p < 0.0001 were determined. (D) NRG increases DUSP6 protein levels in BT474 and BT474Br cells. The cultures were serum-starved for 24 h, treated with NRG (10 ng/mL) and HGF (10 ng/mL) for 48 h, followed by Western blot analysis for DUSP6. (E) The MEK inhibitor trametinib inhibits DUSP6 in HER2+ breast cancer cells. The cells were treated with increasing concentrations of trametinib for 48 h, followed by Western blotting for DUSP6.
