# Supplementary figures and images for: DUSP6 inhibition overcomes neuregulin/HER3-driven therapy tolerance in HER2+ breast cancer (part 1 of 2)
Source: EMBO Mol Med. 2024 Jun 17;16(7):8. doi: 10.1038/s44321-024-00088-0 (PMC11251193; doi:10.1038/s44321-024-00088-0)

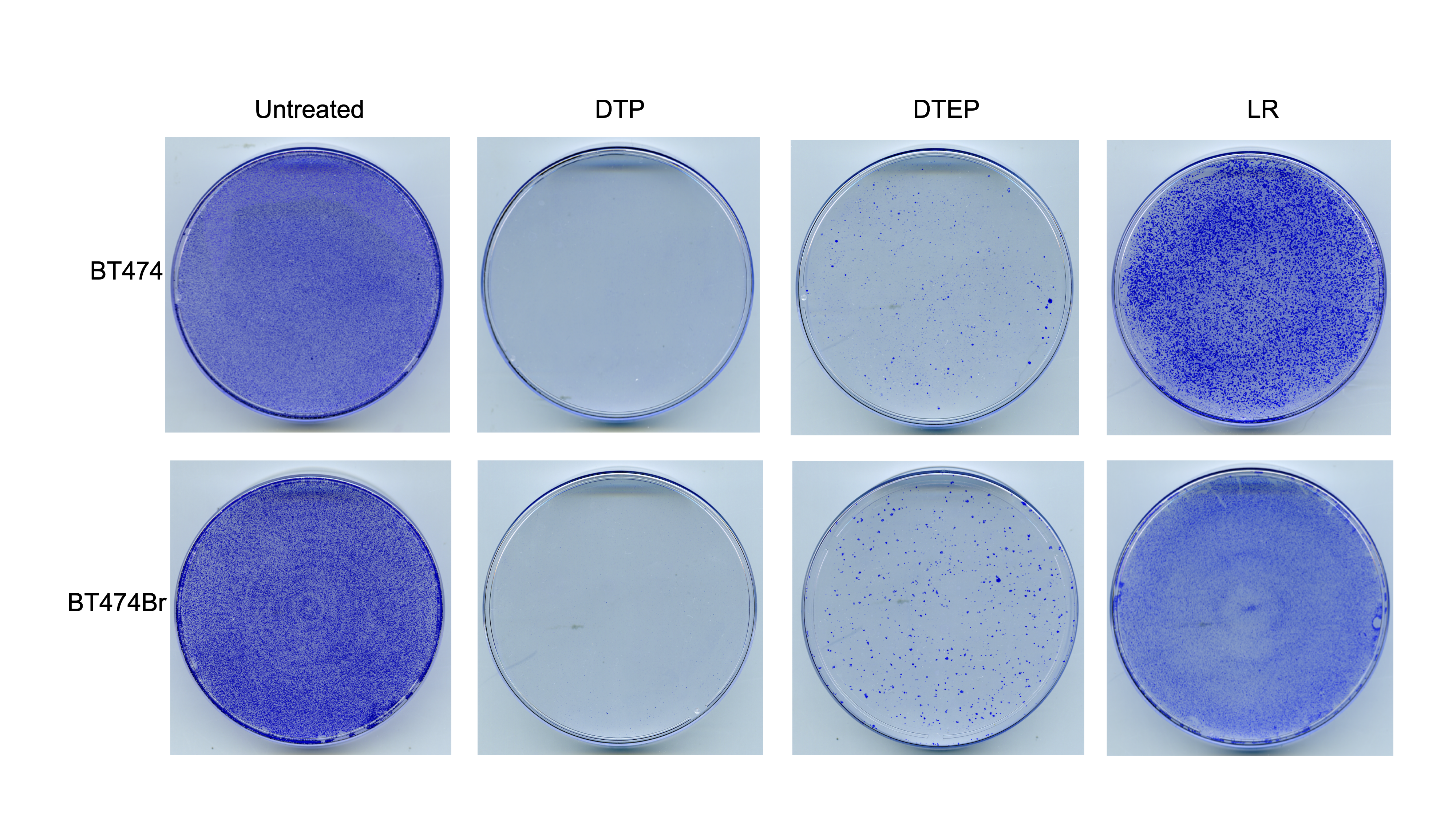

Supplement: Supplementary file 10 — Source data Fig. 1 [file 44321_2024_88_MOESM10_ESM.zip › Figure 1/1A/1A.tiff]

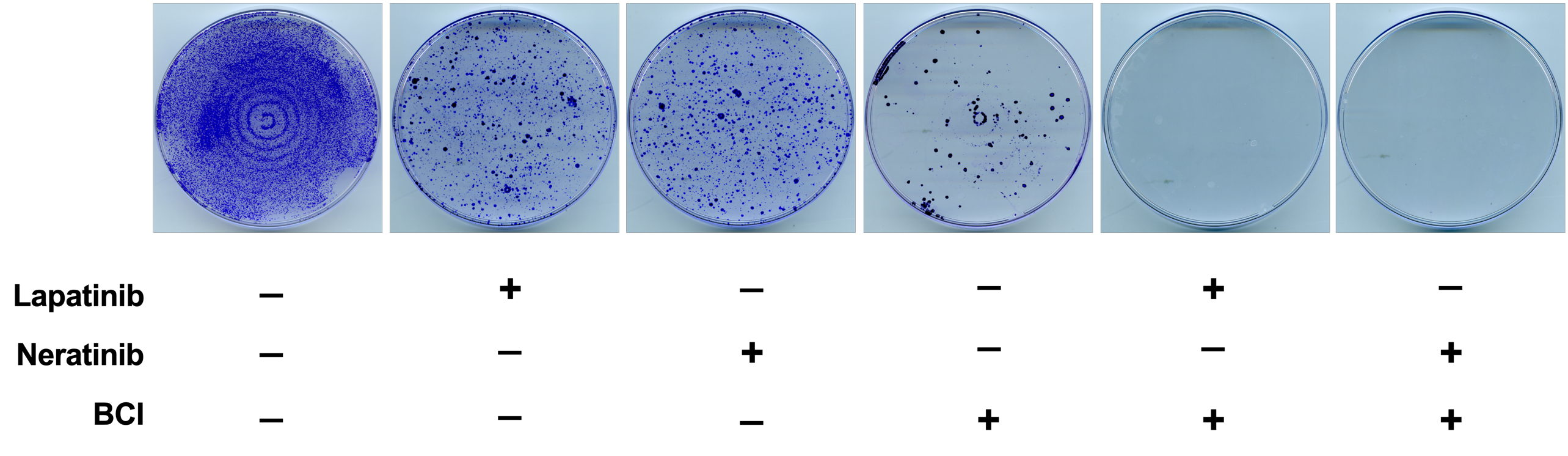

Supplement: Supplementary file 12 — Source data Fig. 3 [file 44321_2024_88_MOESM12_ESM.zip › Figure 3/3G/3G.tiff]

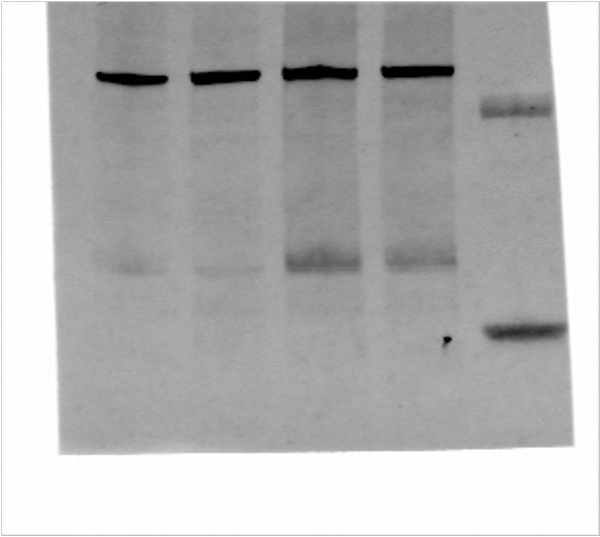

Supplement: Supplementary file 12 — Source data Fig. 3 [file 44321_2024_88_MOESM12_ESM.zip › Figure 3/3A/3rd/DUSP6.tiff]

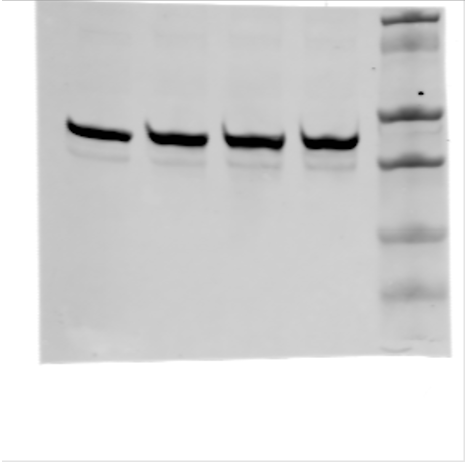

Supplement: Supplementary file 12 — Source data Fig. 3 [file 44321_2024_88_MOESM12_ESM.zip › Figure 3/3A/3rd/B-actin.tiff]

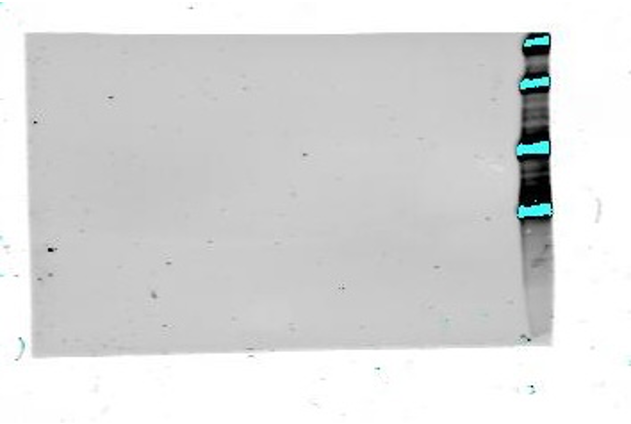

Supplement: Supplementary file 12 — Source data Fig. 3 [file 44321_2024_88_MOESM12_ESM.zip › Figure 3/3A/3rd/DUSP1.tiff]

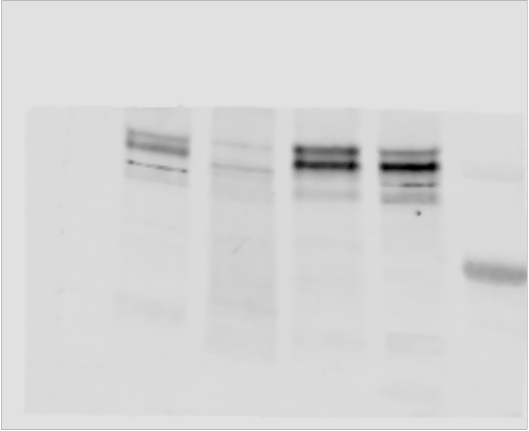

Supplement: Supplementary file 12 — Source data Fig. 3 [file 44321_2024_88_MOESM12_ESM.zip › Figure 3/3A/1st/DUSP6-1.tiff]

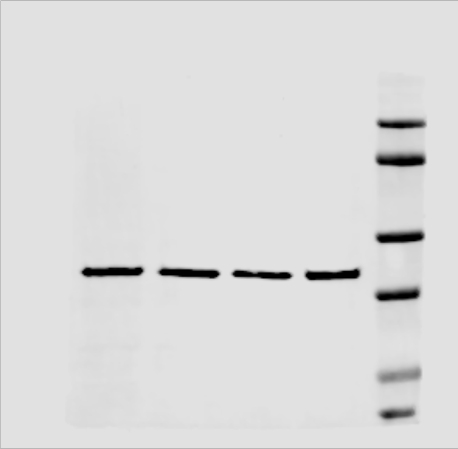

Supplement: Supplementary file 12 — Source data Fig. 3 [file 44321_2024_88_MOESM12_ESM.zip › Figure 3/3A/1st/B-actin 1.tiff]

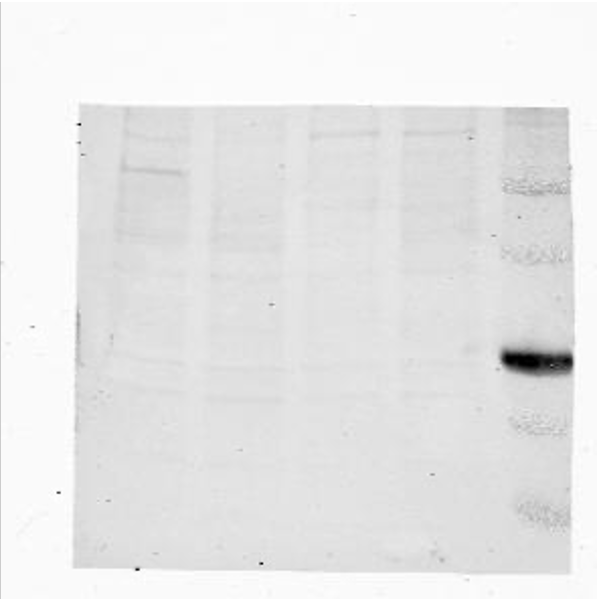

Supplement: Supplementary file 12 — Source data Fig. 3 [file 44321_2024_88_MOESM12_ESM.zip › Figure 3/3A/1st/DUSP1-1.tiff]

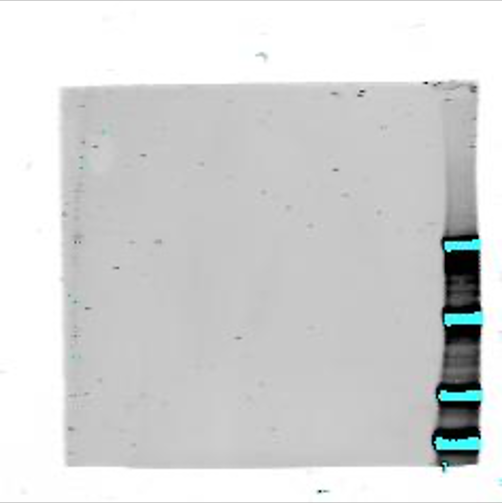

Supplement: Supplementary file 12 — Source data Fig. 3 [file 44321_2024_88_MOESM12_ESM.zip › Figure 3/3A/2nd/DUSP1-2.tiff]

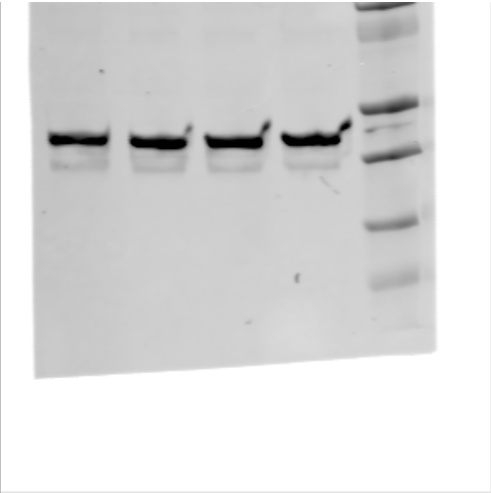

Supplement: Supplementary file 12 — Source data Fig. 3 [file 44321_2024_88_MOESM12_ESM.zip › Figure 3/3A/2nd/B-actin 2.tiff]

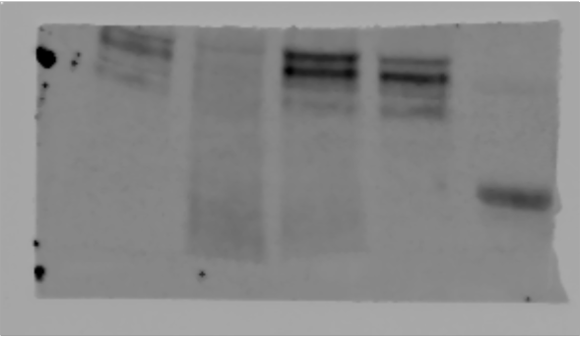

Supplement: Supplementary file 12 — Source data Fig. 3 [file 44321_2024_88_MOESM12_ESM.zip › Figure 3/3A/2nd/DUSP6-2.tiff]

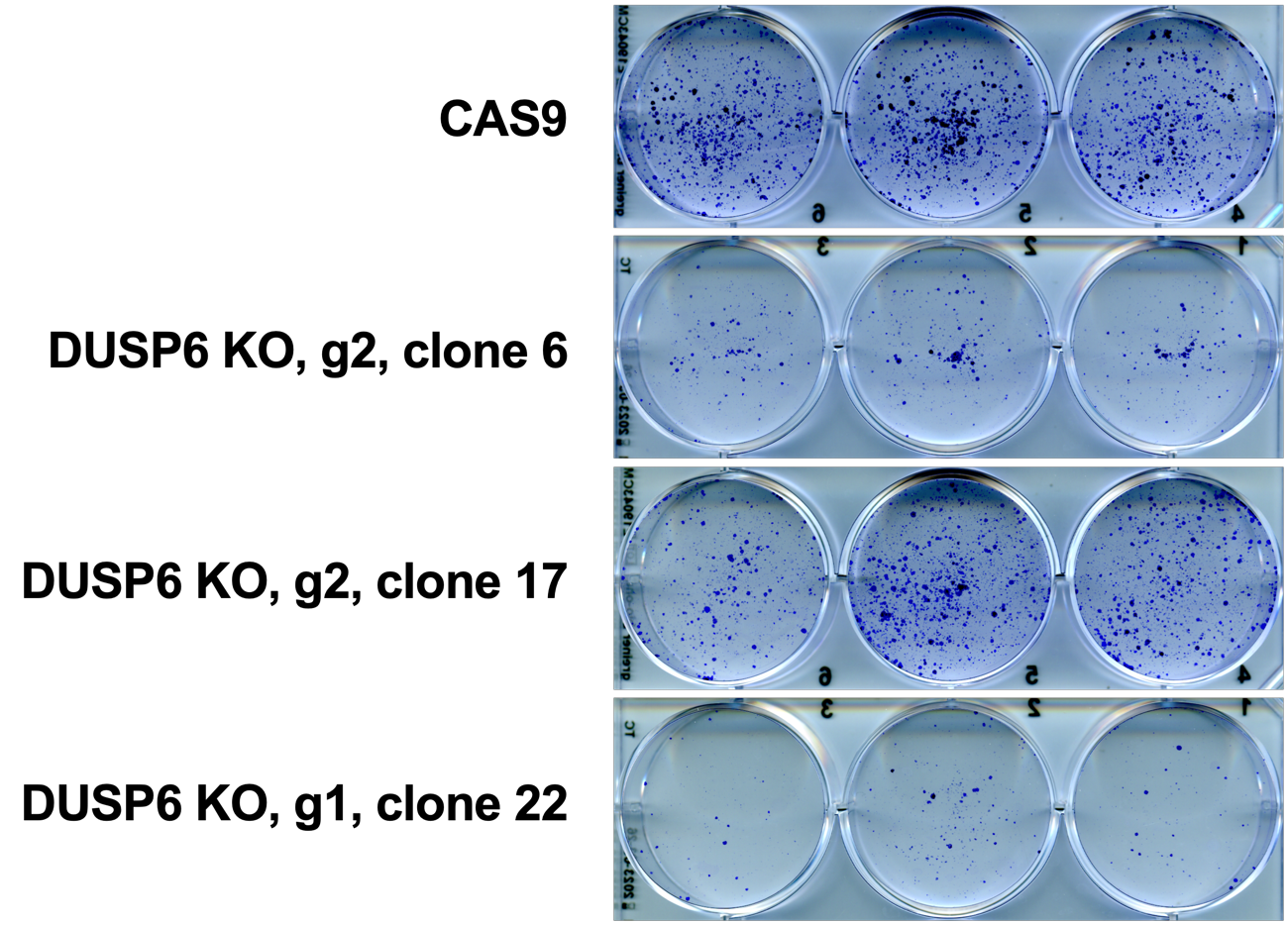

Supplement: Supplementary file 13 — Source data Fig. 4 [file 44321_2024_88_MOESM13_ESM.zip › Figure 4/4B/4B.tiff]

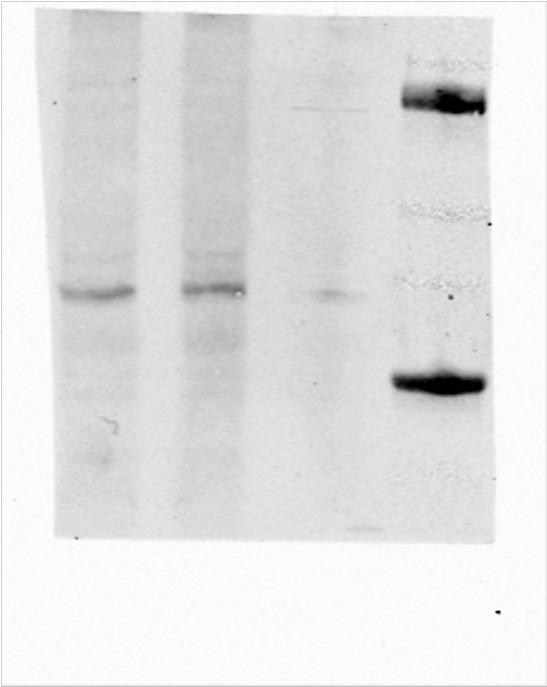

Supplement: Supplementary file 13 — Source data Fig. 4 [file 44321_2024_88_MOESM13_ESM.zip › Figure 4/4A/3rd/DUSP6.tiff]

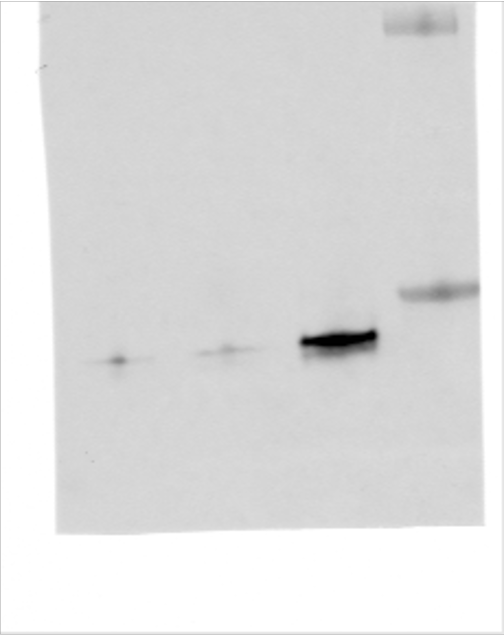

Supplement: Supplementary file 13 — Source data Fig. 4 [file 44321_2024_88_MOESM13_ESM.zip › Figure 4/4A/3rd/Cl-PARP, 25.tiff]

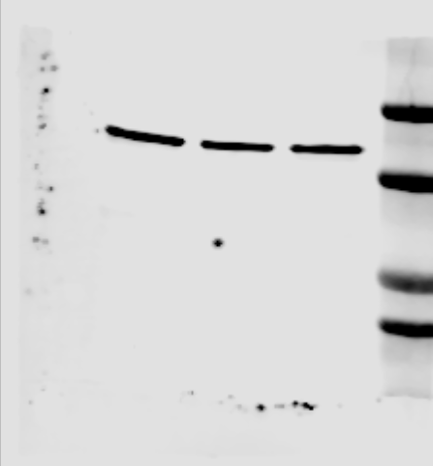

Supplement: Supplementary file 13 — Source data Fig. 4 [file 44321_2024_88_MOESM13_ESM.zip › Figure 4/4A/3rd/B-actin.tiff]

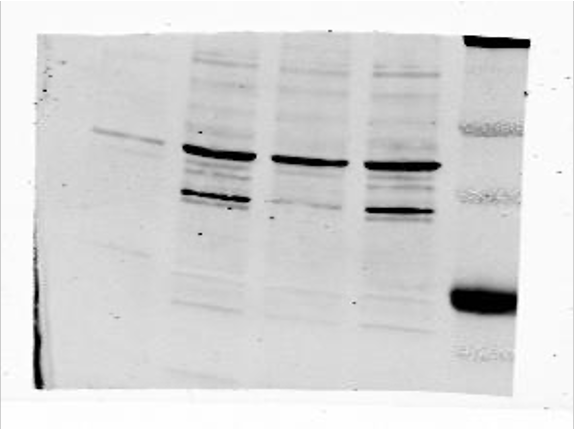

Supplement: Supplementary file 13 — Source data Fig. 4 [file 44321_2024_88_MOESM13_ESM.zip › Figure 4/4A/3rd/DUSP1.tiff]

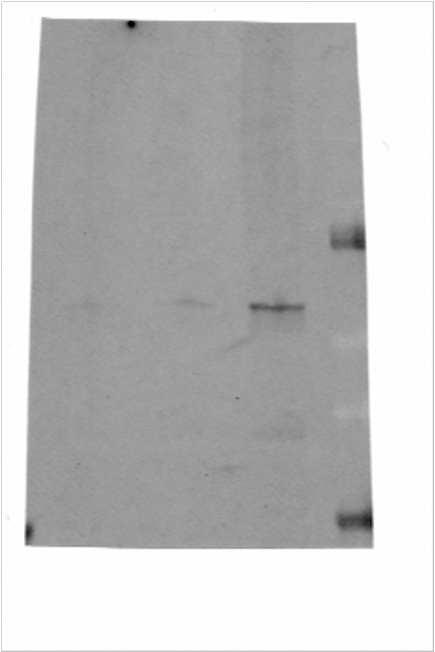

Supplement: Supplementary file 13 — Source data Fig. 4 [file 44321_2024_88_MOESM13_ESM.zip › Figure 4/4A/3rd/Cl-PARP, 89.tiff]

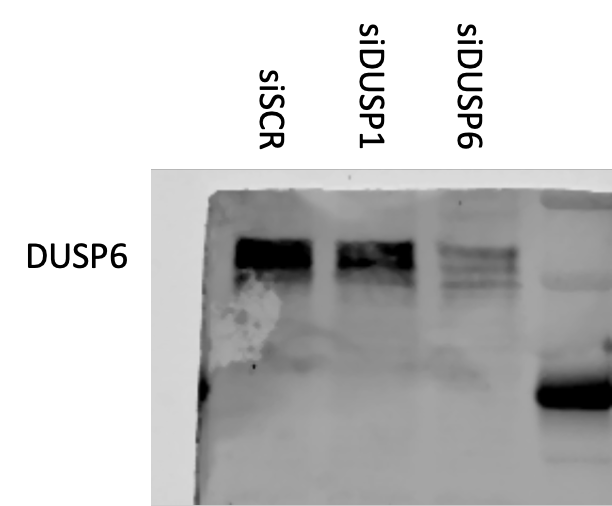

Supplement: Supplementary file 13 — Source data Fig. 4 [file 44321_2024_88_MOESM13_ESM.zip › Figure 4/4A/1st/DUSP6.tiff]

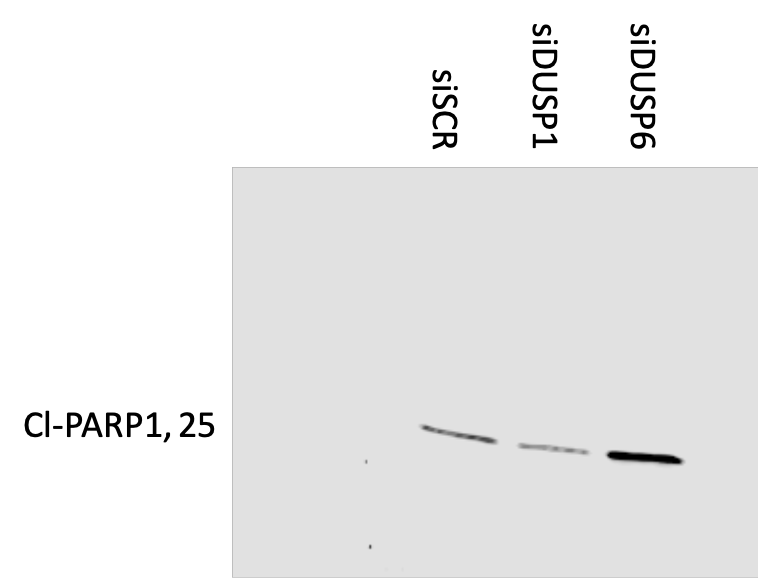

Supplement: Supplementary file 13 — Source data Fig. 4 [file 44321_2024_88_MOESM13_ESM.zip › Figure 4/4A/1st/Cl-PARP, 25.tiff]

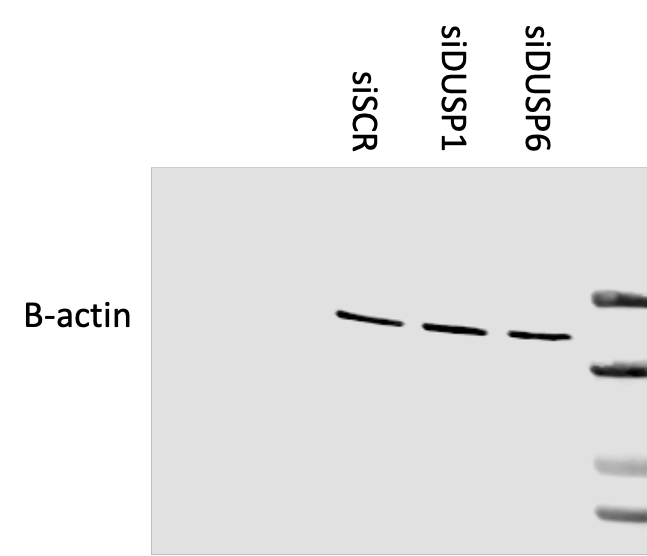

Supplement: Supplementary file 13 — Source data Fig. 4 [file 44321_2024_88_MOESM13_ESM.zip › Figure 4/4A/1st/B-actin.tiff]

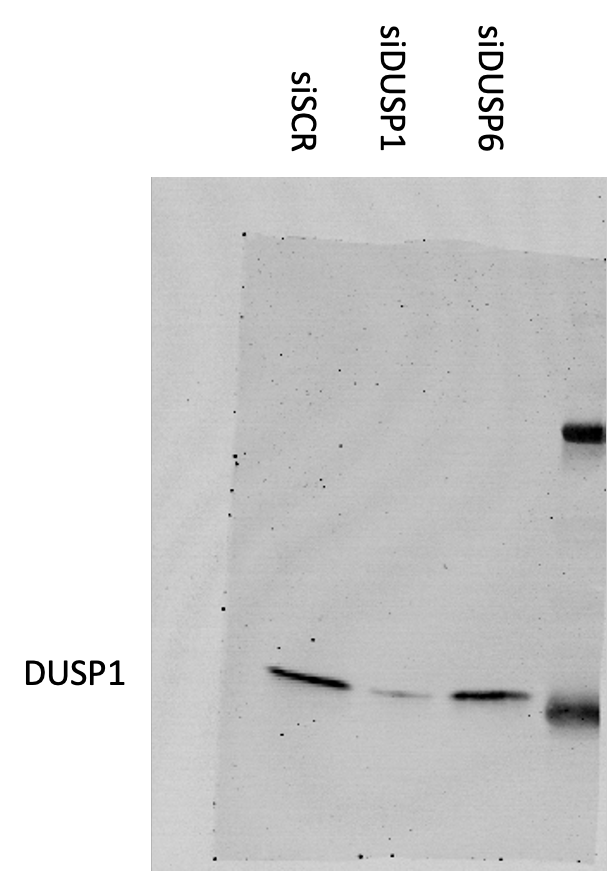

Supplement: Supplementary file 13 — Source data Fig. 4 [file 44321_2024_88_MOESM13_ESM.zip › Figure 4/4A/1st/DUSP1.tiff]

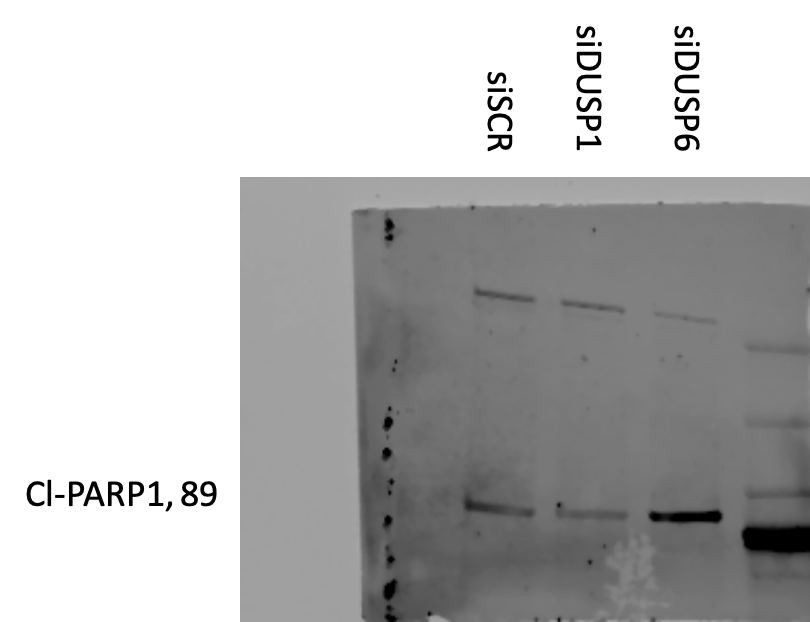

Supplement: Supplementary file 13 — Source data Fig. 4 [file 44321_2024_88_MOESM13_ESM.zip › Figure 4/4A/1st/Cl-PARP, 89.tiff]

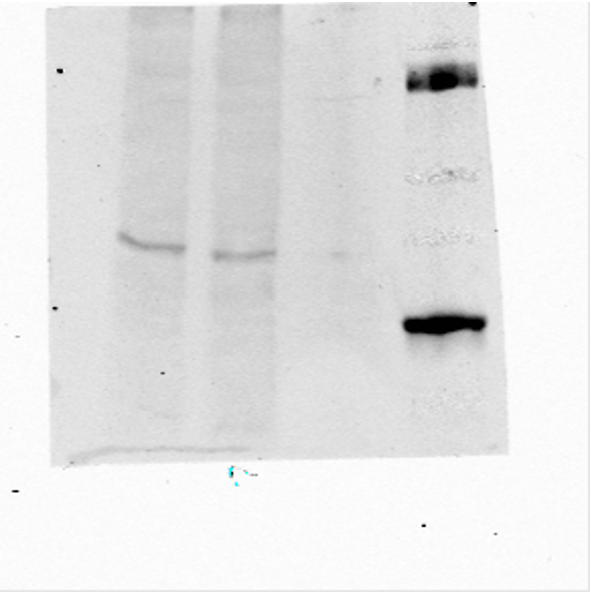

Supplement: Supplementary file 13 — Source data Fig. 4 [file 44321_2024_88_MOESM13_ESM.zip › Figure 4/4A/2nd/DUSP6.tiff]

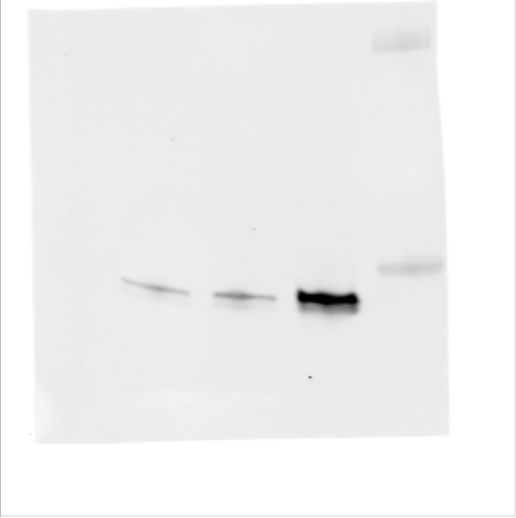

Supplement: Supplementary file 13 — Source data Fig. 4 [file 44321_2024_88_MOESM13_ESM.zip › Figure 4/4A/2nd/Cl-PARP, 25.tiff]

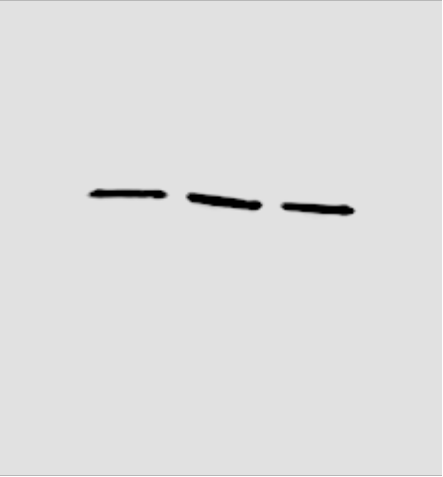

Supplement: Supplementary file 13 — Source data Fig. 4 [file 44321_2024_88_MOESM13_ESM.zip › Figure 4/4A/2nd/B-actin.tiff]

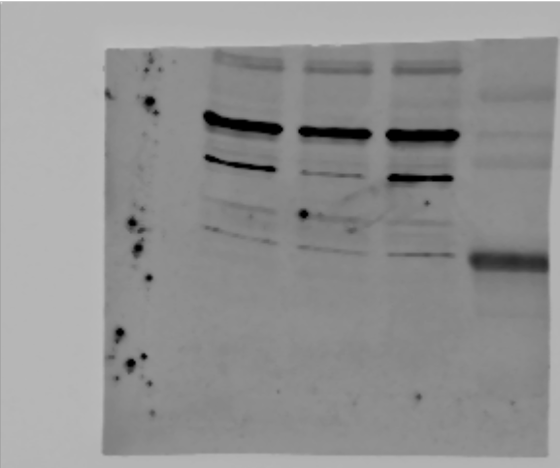

Supplement: Supplementary file 13 — Source data Fig. 4 [file 44321_2024_88_MOESM13_ESM.zip › Figure 4/4A/2nd/DUSP1.tiff]

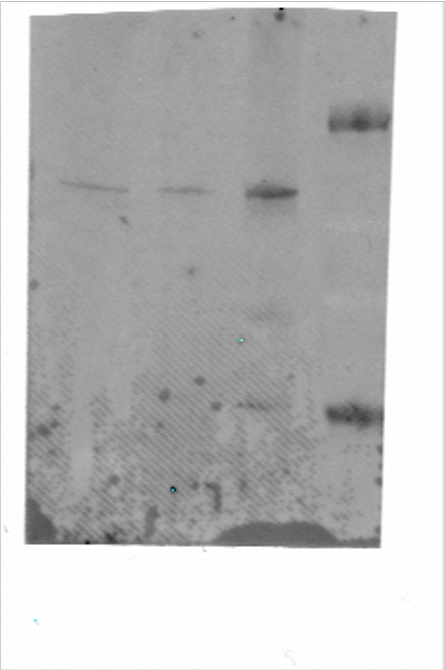

Supplement: Supplementary file 13 — Source data Fig. 4 [file 44321_2024_88_MOESM13_ESM.zip › Figure 4/4A/2nd/Cl-PARP, 89.tiff]

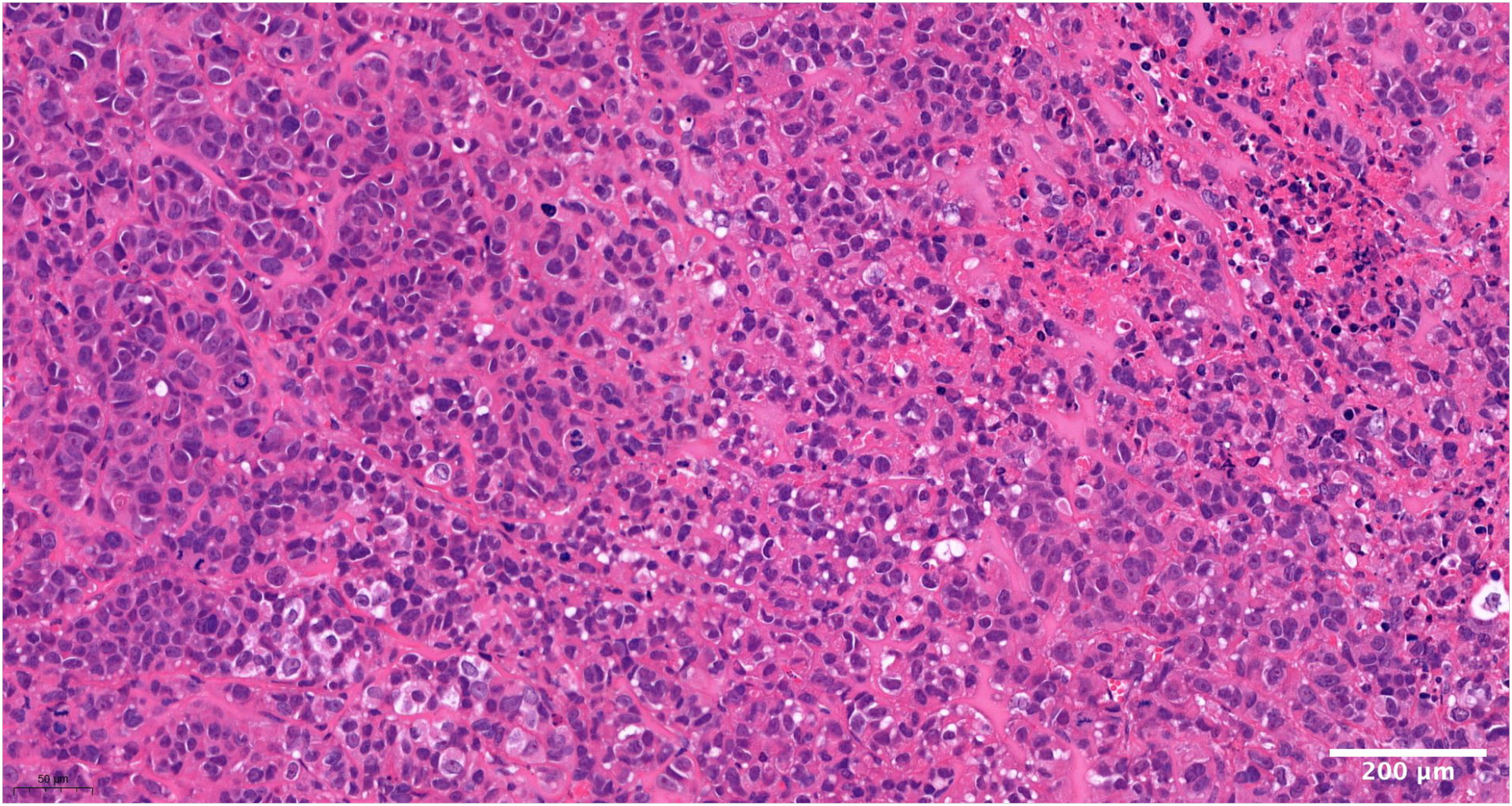

Supplement: Supplementary file 14 — Source data Fig. 5 [file 44321_2024_88_MOESM14_ESM.zip › Figure 5/5F/BCI.tiff]

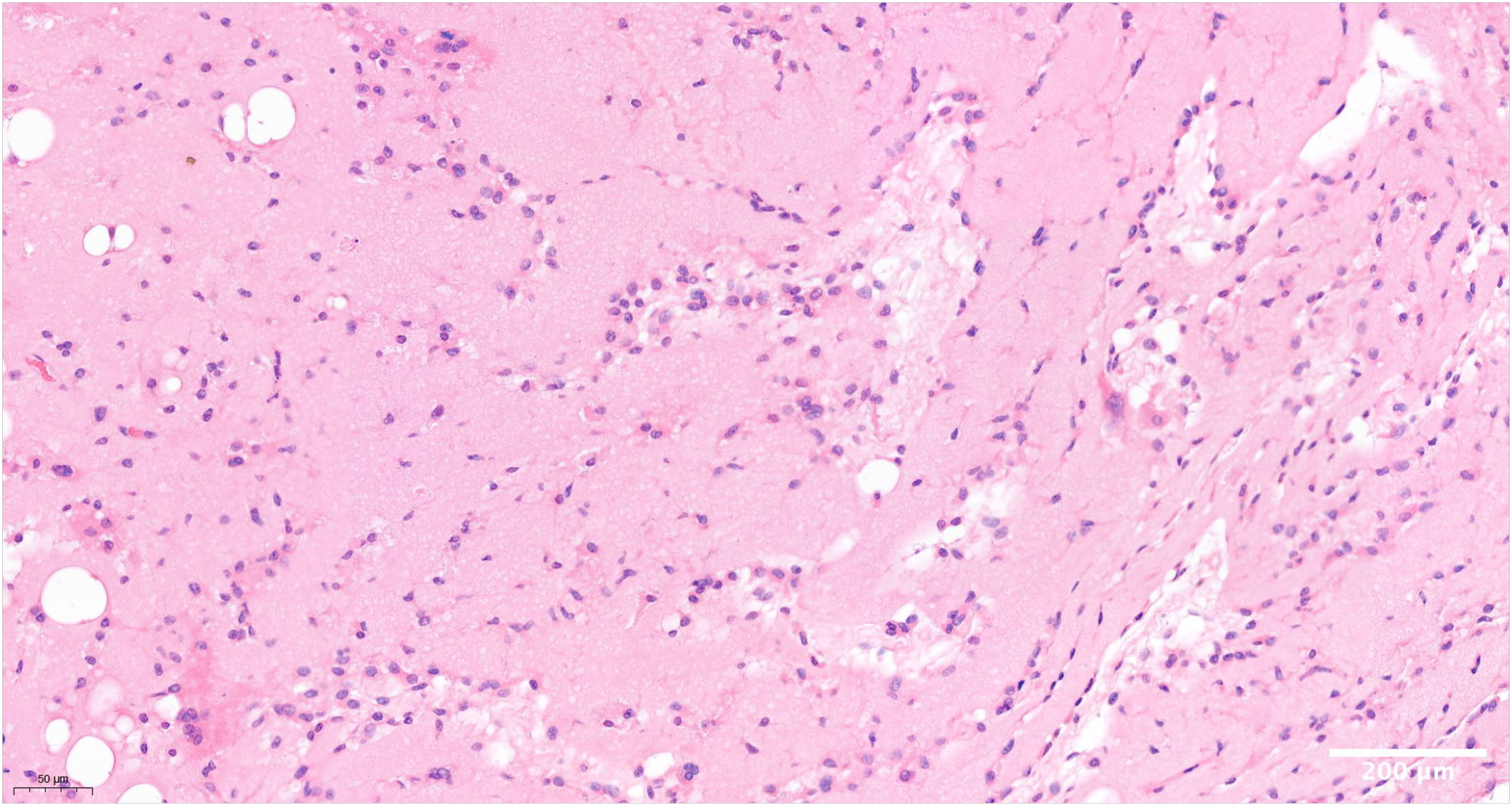

Supplement: Supplementary file 14 — Source data Fig. 5 [file 44321_2024_88_MOESM14_ESM.zip › Figure 5/5F/Lapatinib+BCI.tiff]

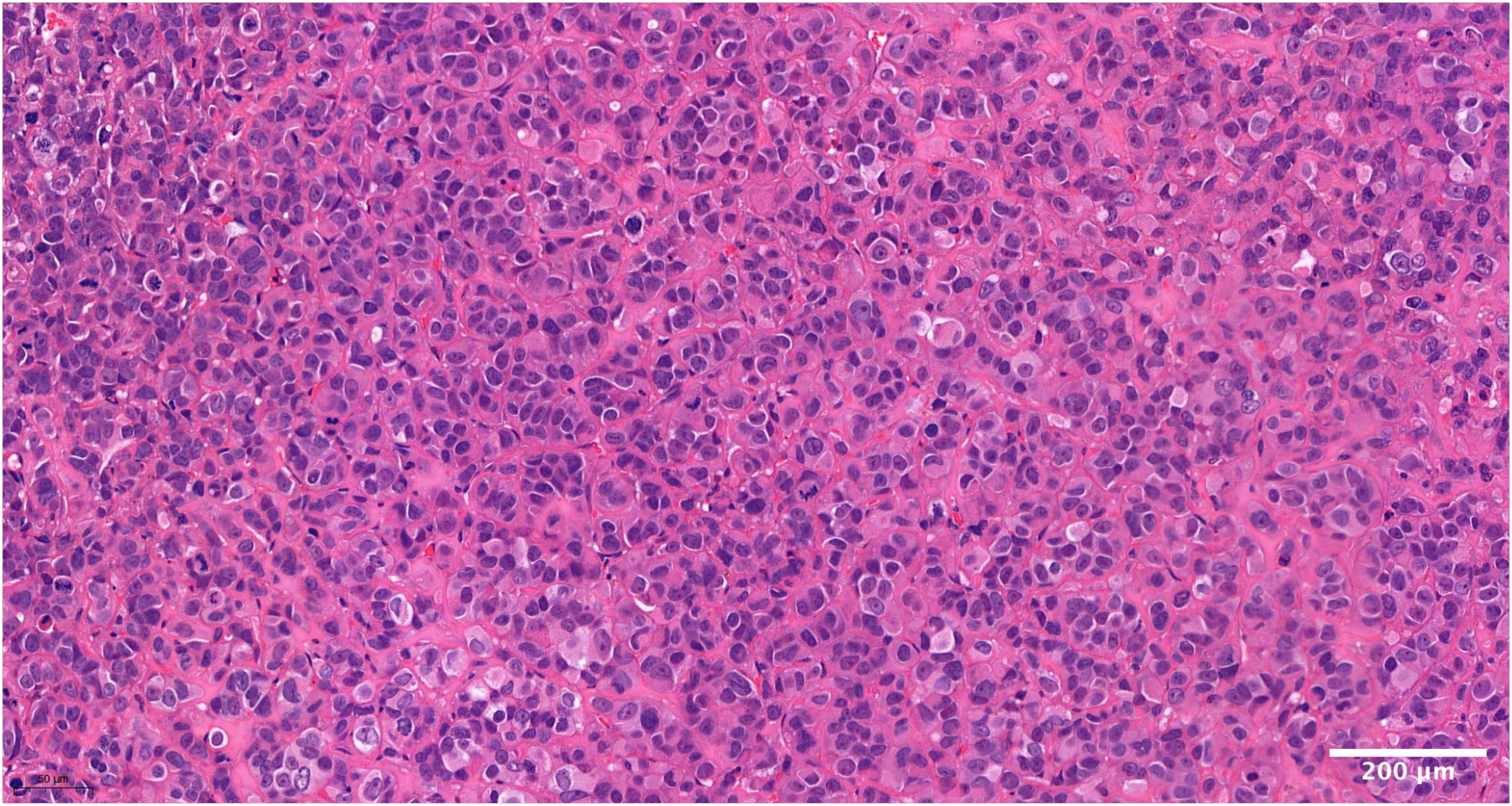

Supplement: Supplementary file 14 — Source data Fig. 5 [file 44321_2024_88_MOESM14_ESM.zip › Figure 5/5F/Control.tiff]

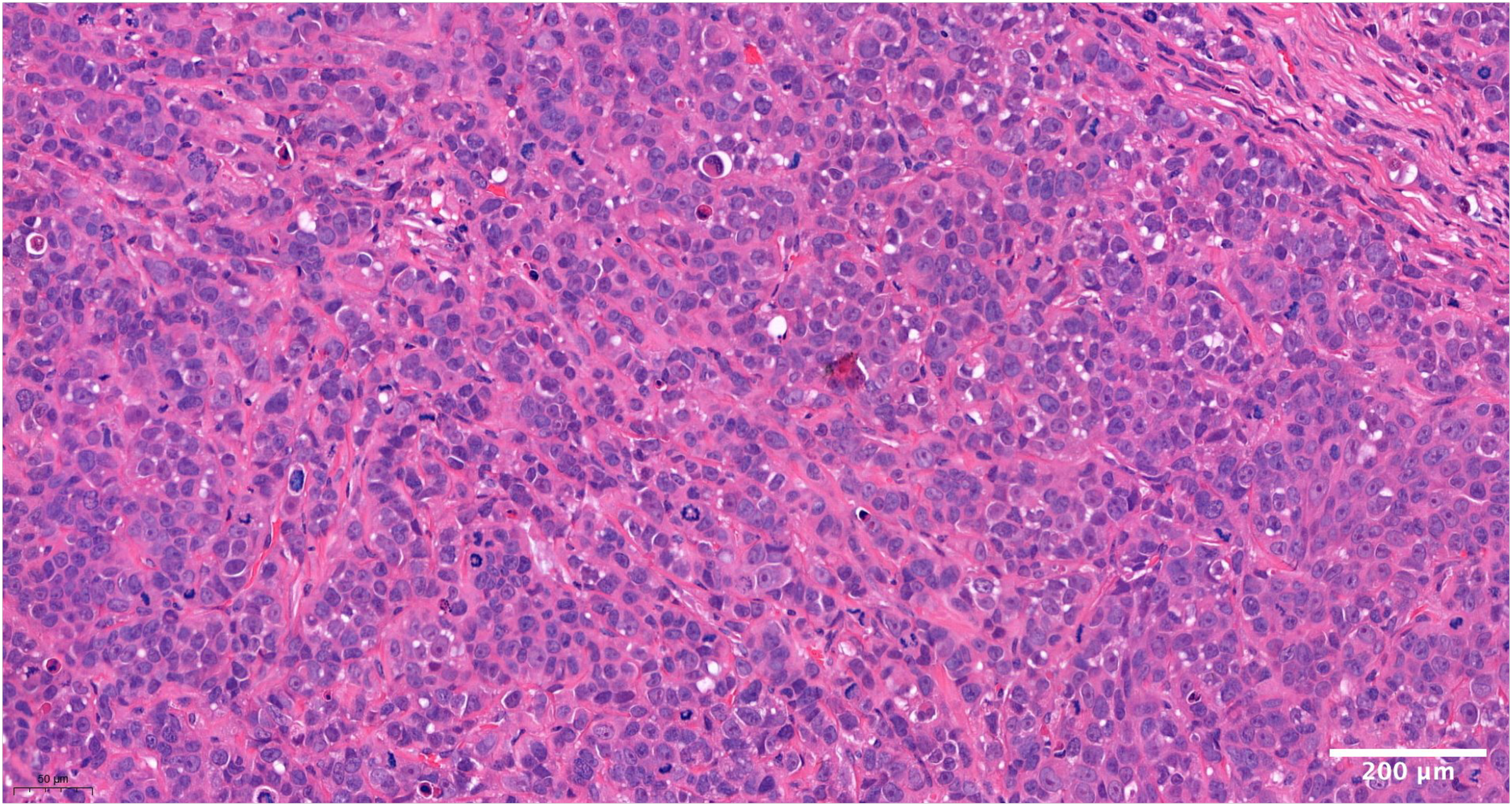

Supplement: Supplementary file 14 — Source data Fig. 5 [file 44321_2024_88_MOESM14_ESM.zip › Figure 5/5F/Lapatinib.tiff]

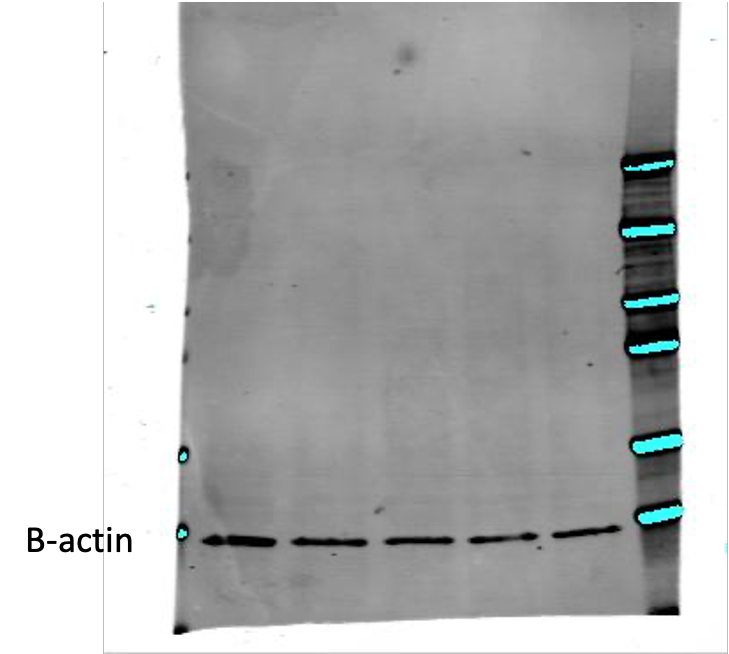

Supplement: Supplementary file 15 — Source data Fig. 6 [file 44321_2024_88_MOESM15_ESM.zip › Figure 6/6F/B-actin.tiff]

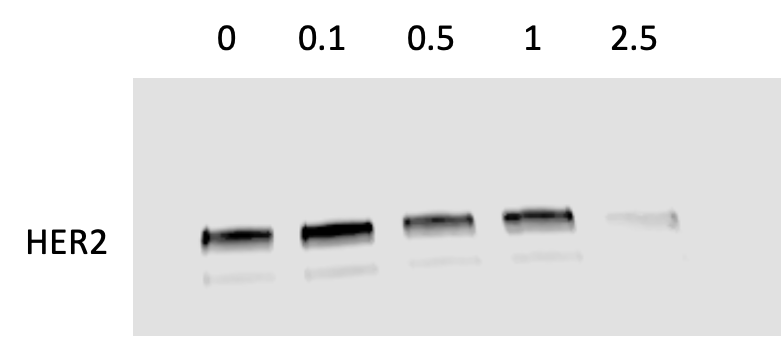

Supplement: Supplementary file 15 — Source data Fig. 6 [file 44321_2024_88_MOESM15_ESM.zip › Figure 6/6F/HER2.tiff]

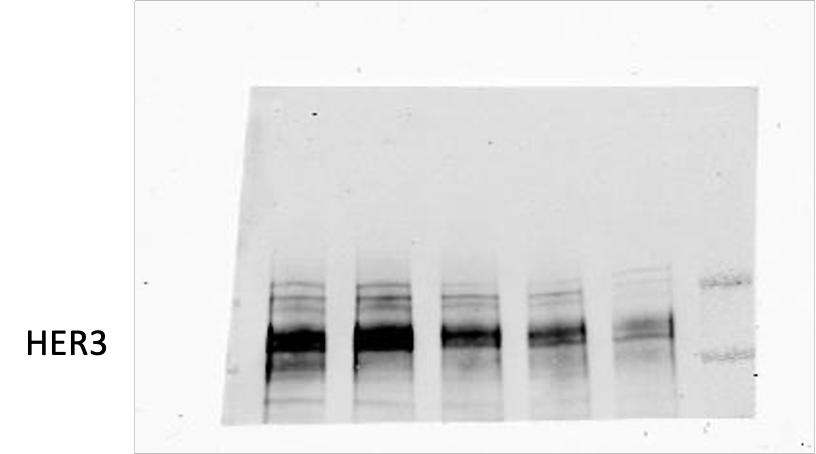

Supplement: Supplementary file 15 — Source data Fig. 6 [file 44321_2024_88_MOESM15_ESM.zip › Figure 6/6F/HER3.tiff]

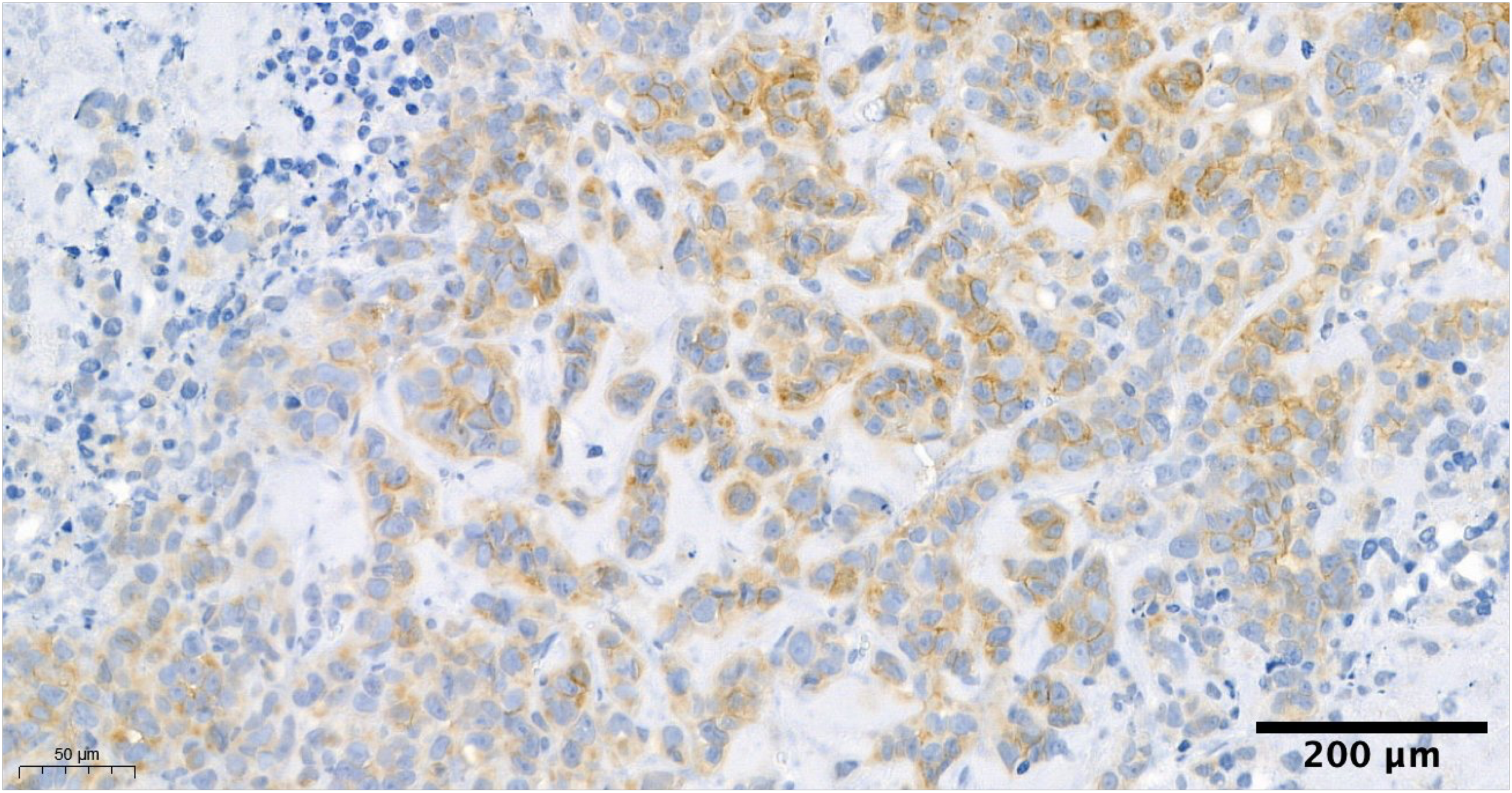

Supplement: Supplementary file 15 — Source data Fig. 6 [file 44321_2024_88_MOESM15_ESM.zip › Figure 6/6I/HER3, BCI.tiff]

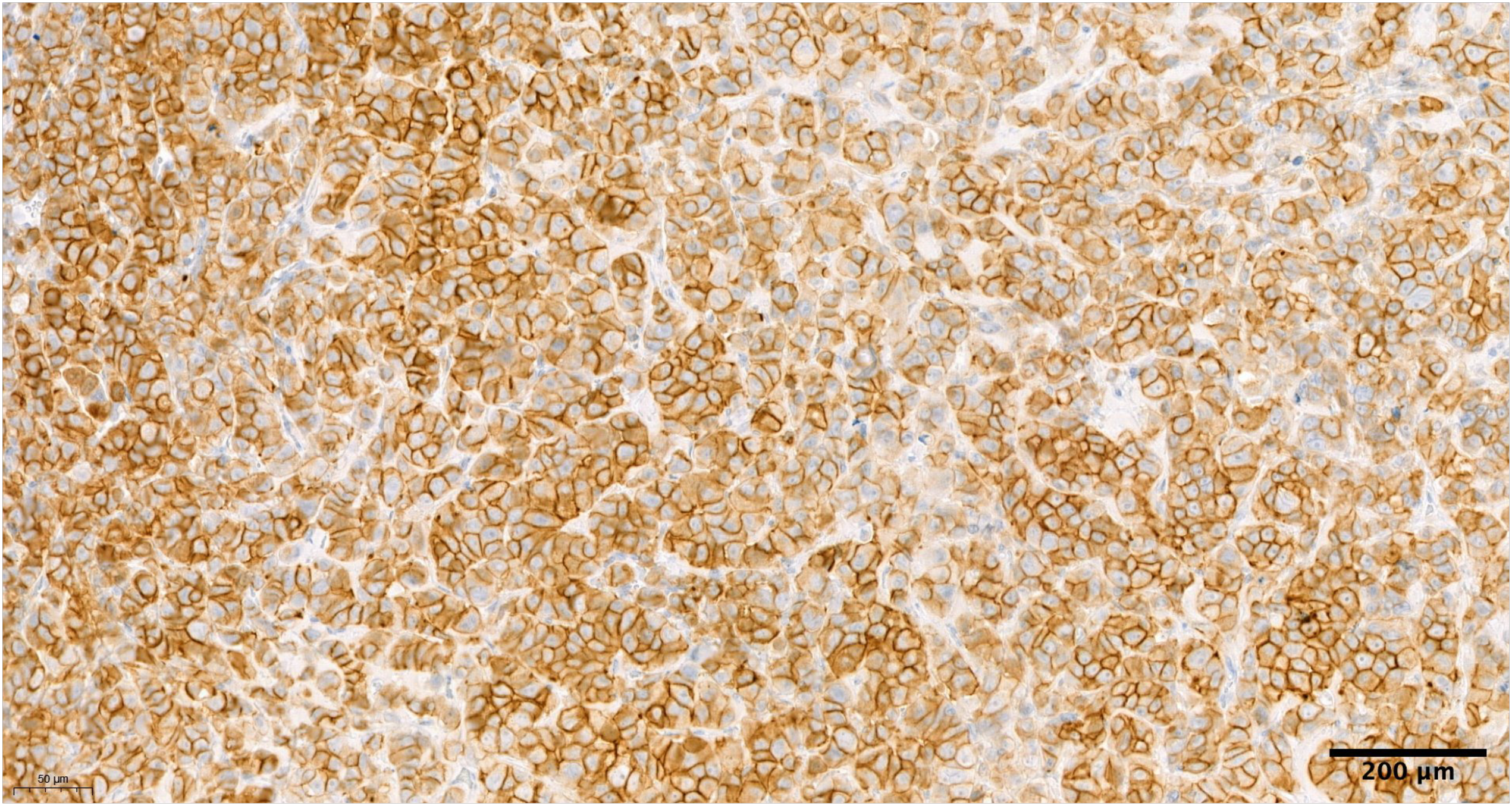

Supplement: Supplementary file 15 — Source data Fig. 6 [file 44321_2024_88_MOESM15_ESM.zip › Figure 6/6I/HER2, control.tiff]

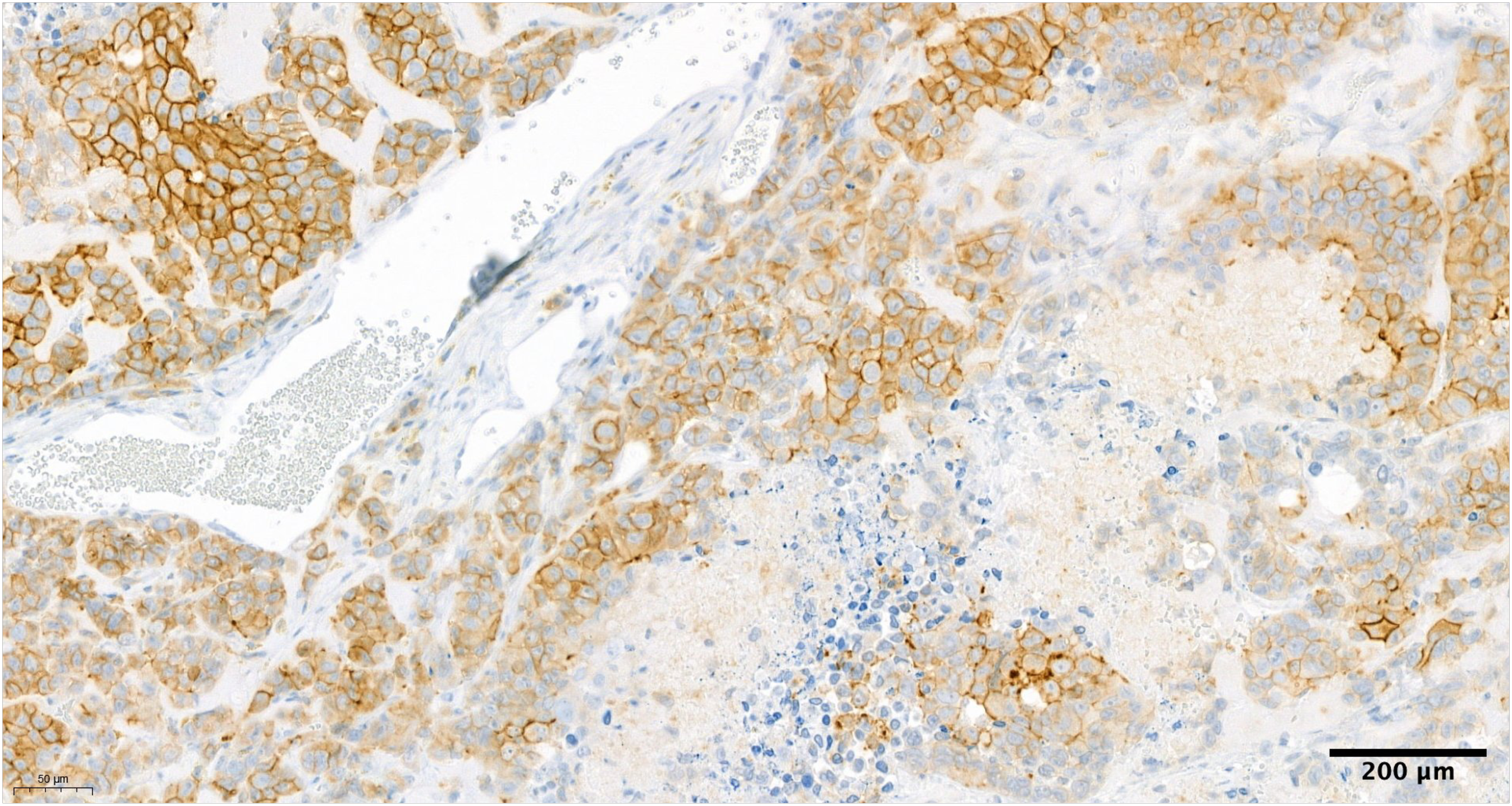

Supplement: Supplementary file 15 — Source data Fig. 6 [file 44321_2024_88_MOESM15_ESM.zip › Figure 6/6I/HER2, BCI.tiff]

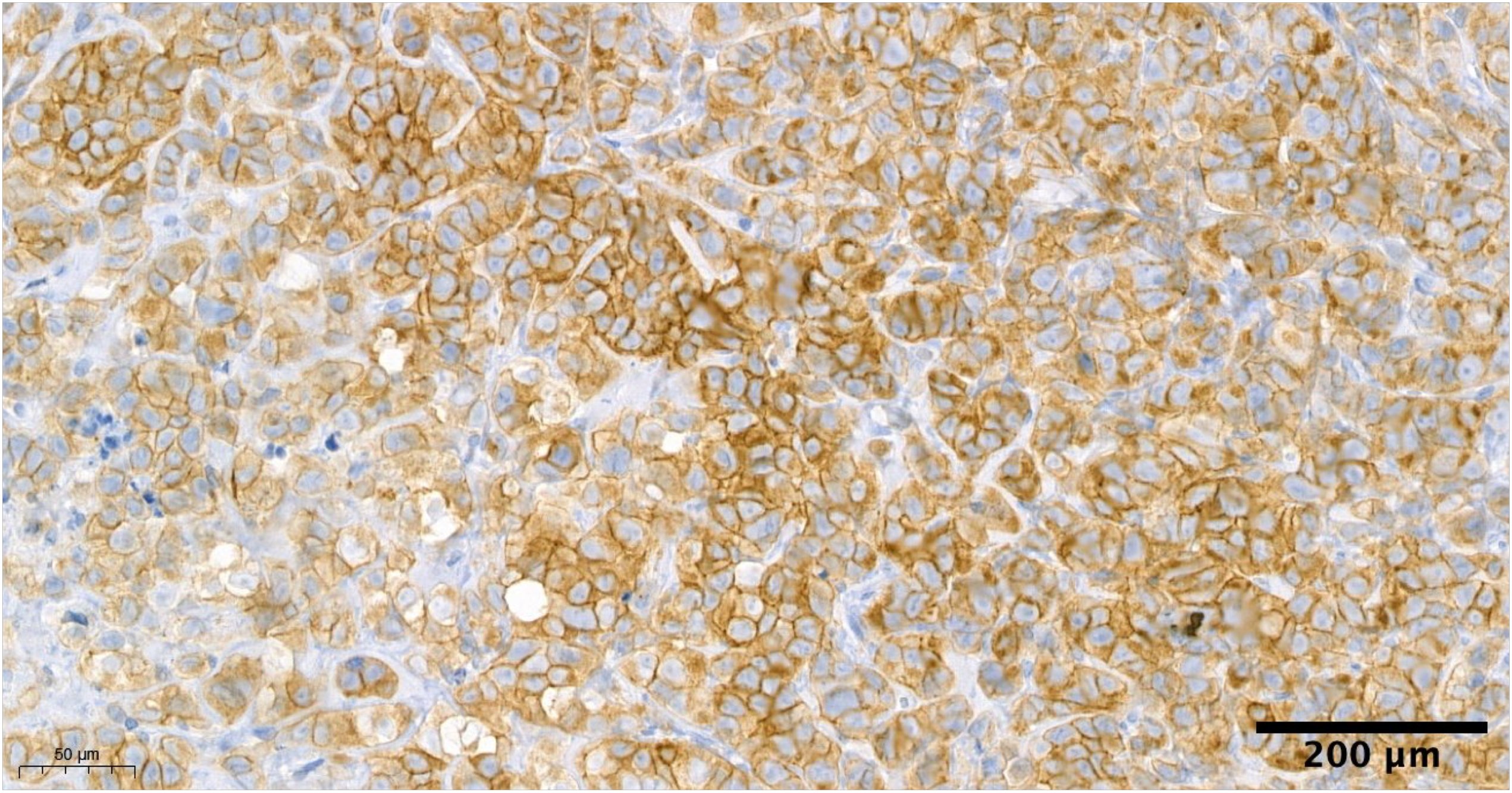

Supplement: Supplementary file 15 — Source data Fig. 6 [file 44321_2024_88_MOESM15_ESM.zip › Figure 6/6I/HER3, control.tiff]

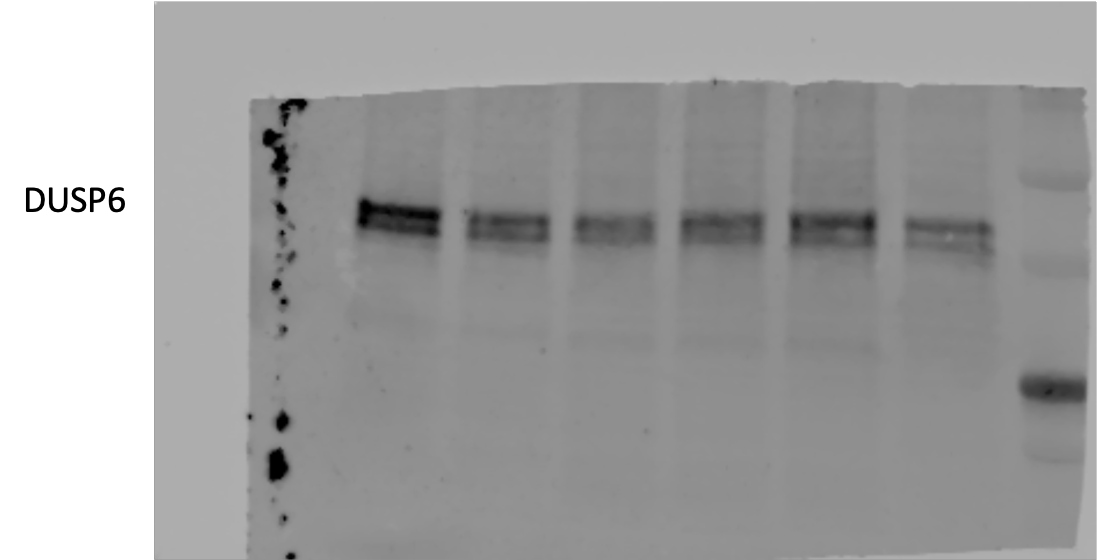

Supplement: Supplementary file 15 — Source data Fig. 6 [file 44321_2024_88_MOESM15_ESM.zip › Figure 6/6G/DUSP6.tiff]

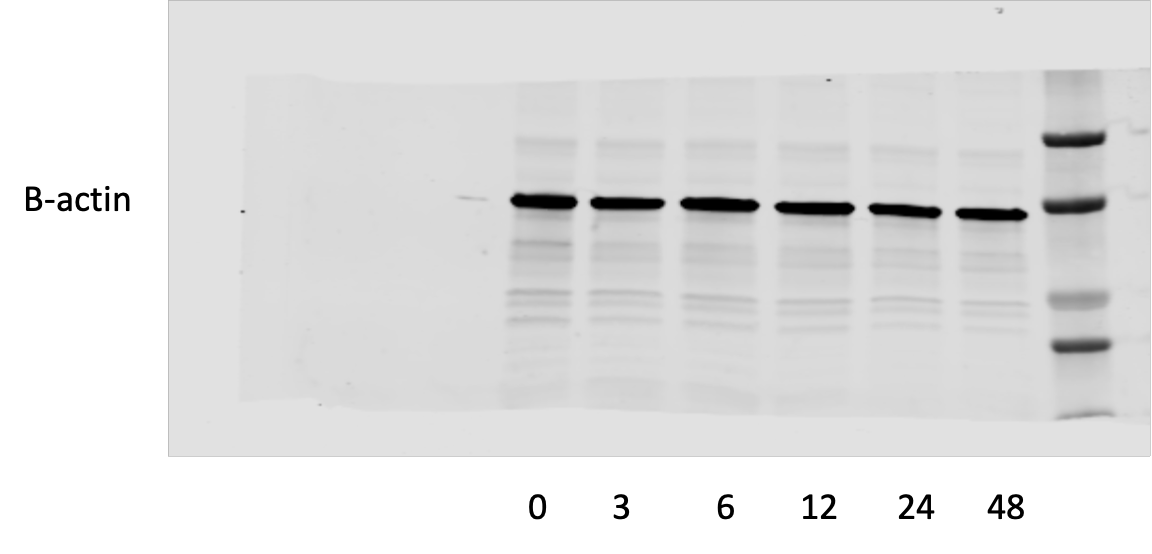

Supplement: Supplementary file 15 — Source data Fig. 6 [file 44321_2024_88_MOESM15_ESM.zip › Figure 6/6G/B-actin.tiff]

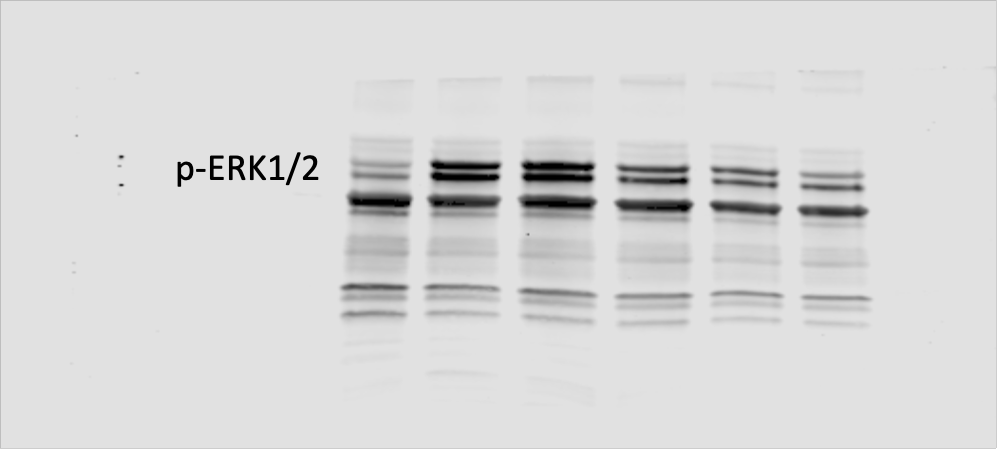

Supplement: Supplementary file 15 — Source data Fig. 6 [file 44321_2024_88_MOESM15_ESM.zip › Figure 6/6G/p-ERK1:2.tiff]

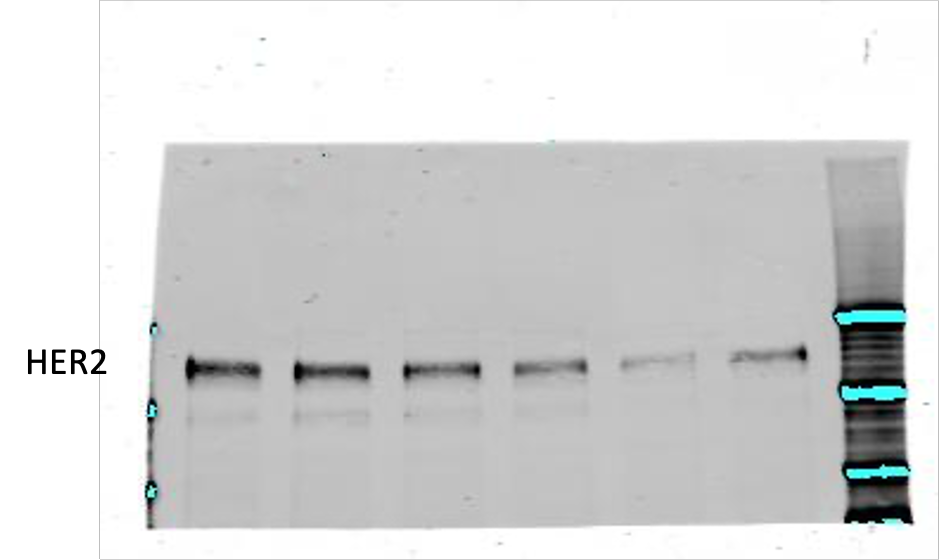

Supplement: Supplementary file 15 — Source data Fig. 6 [file 44321_2024_88_MOESM15_ESM.zip › Figure 6/6G/HER2.tiff]

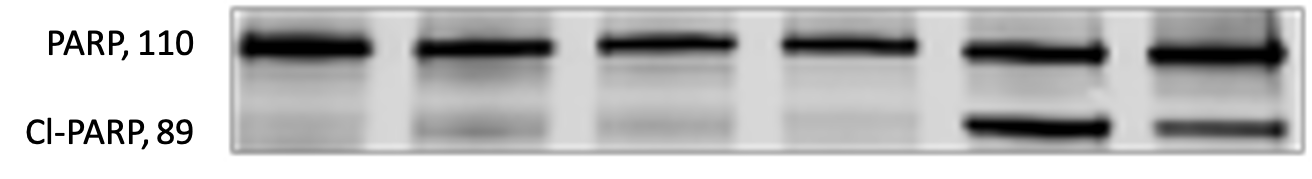

Supplement: Supplementary file 15 — Source data Fig. 6 [file 44321_2024_88_MOESM15_ESM.zip › Figure 6/6G/Cl-PARP, 89.tiff]

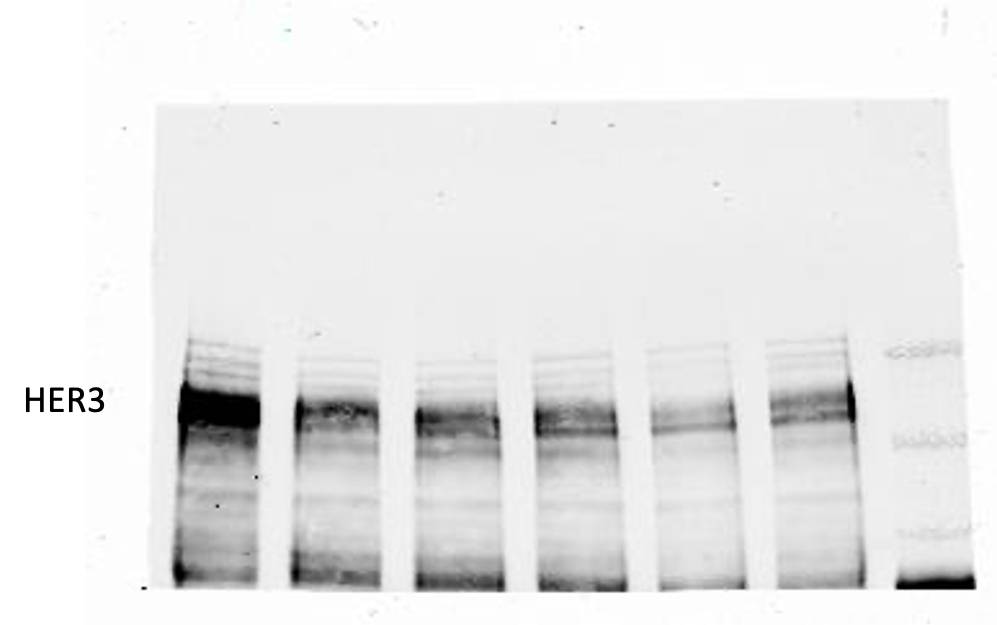

Supplement: Supplementary file 15 — Source data Fig. 6 [file 44321_2024_88_MOESM15_ESM.zip › Figure 6/6G/HER3.png]

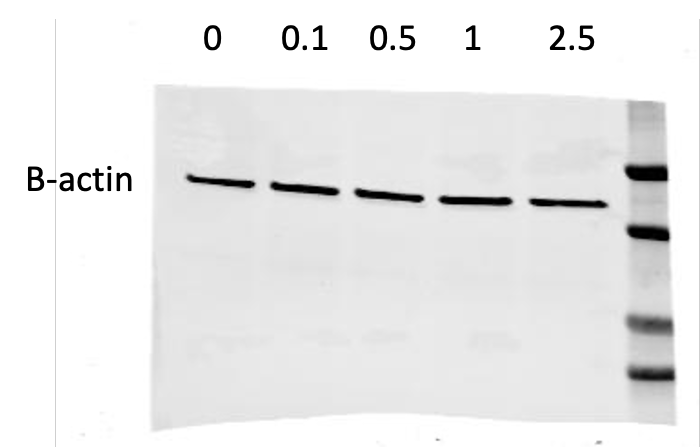

Supplement: Supplementary file 15 — Source data Fig. 6 [file 44321_2024_88_MOESM15_ESM.zip › Figure 6/6E/B-actin.tiff]

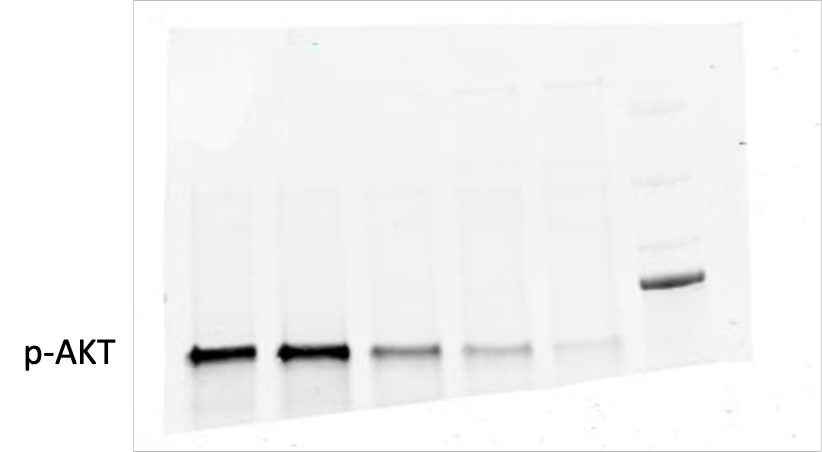

Supplement: Supplementary file 15 — Source data Fig. 6 [file 44321_2024_88_MOESM15_ESM.zip › Figure 6/6E/p-AKT.tiff]

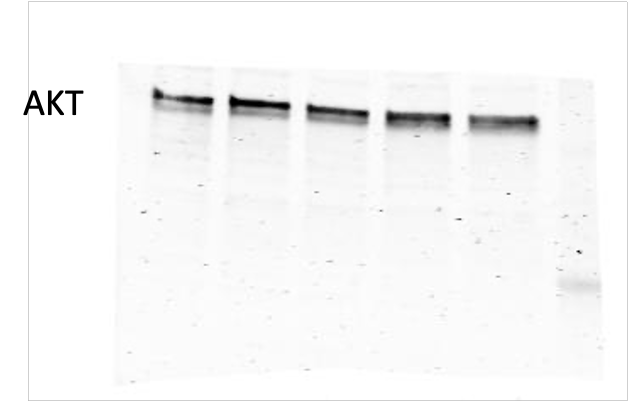

Supplement: Supplementary file 15 — Source data Fig. 6 [file 44321_2024_88_MOESM15_ESM.zip › Figure 6/6E/AKT.tiff]

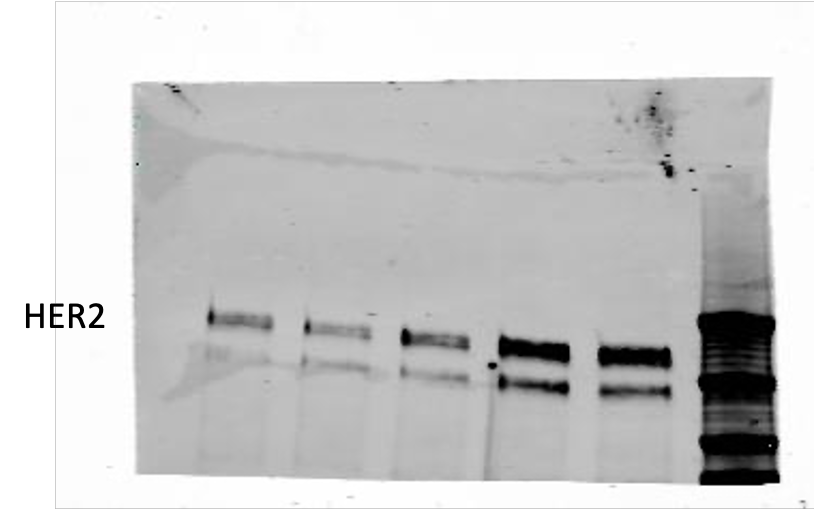

Supplement: Supplementary file 15 — Source data Fig. 6 [file 44321_2024_88_MOESM15_ESM.zip › Figure 6/6E/HER2.tiff]

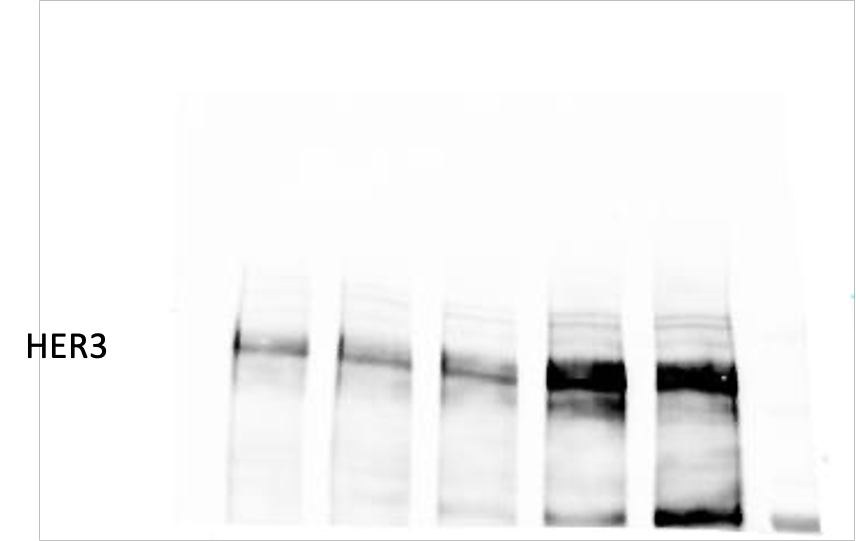

Supplement: Supplementary file 15 — Source data Fig. 6 [file 44321_2024_88_MOESM15_ESM.zip › Figure 6/6E/HER3.tiff]

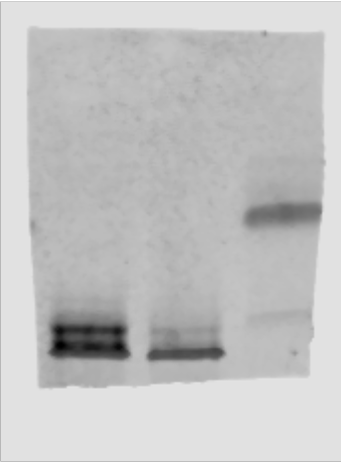

Supplement: Supplementary file 15 — Source data Fig. 6 [file 44321_2024_88_MOESM15_ESM.zip › Figure 6/6H/3rd/DUSP6.tiff]

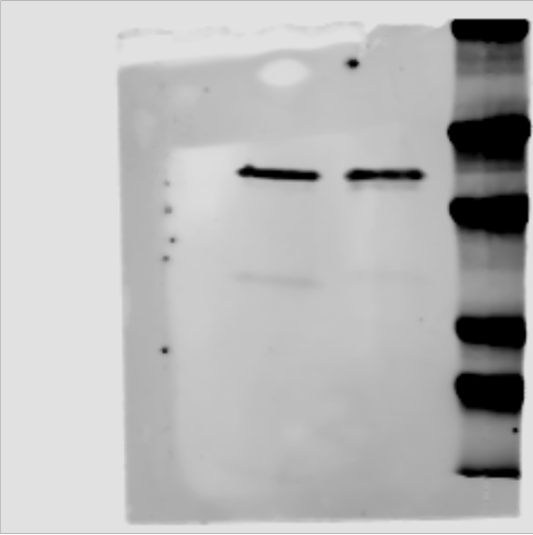

Supplement: Supplementary file 15 — Source data Fig. 6 [file 44321_2024_88_MOESM15_ESM.zip › Figure 6/6H/3rd/B-actin.tiff]

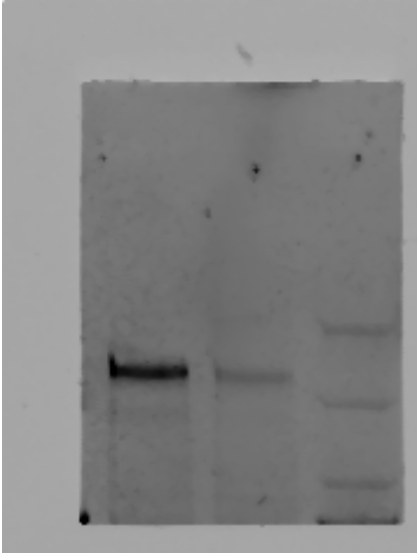

Supplement: Supplementary file 15 — Source data Fig. 6 [file 44321_2024_88_MOESM15_ESM.zip › Figure 6/6H/3rd/HER2.tiff]

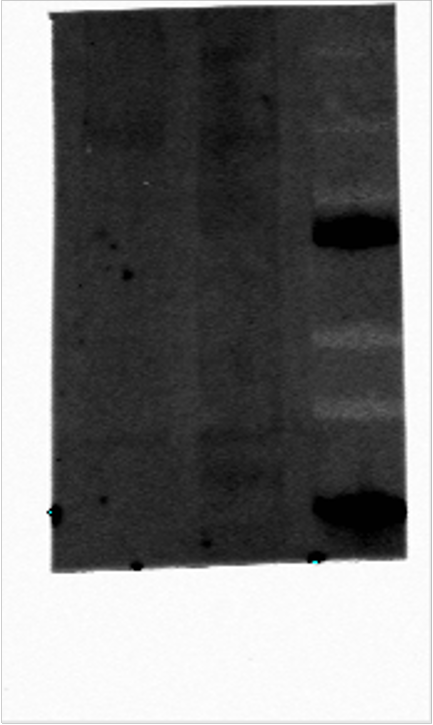

Supplement: Supplementary file 15 — Source data Fig. 6 [file 44321_2024_88_MOESM15_ESM.zip › Figure 6/6H/3rd/HER3.tiff]

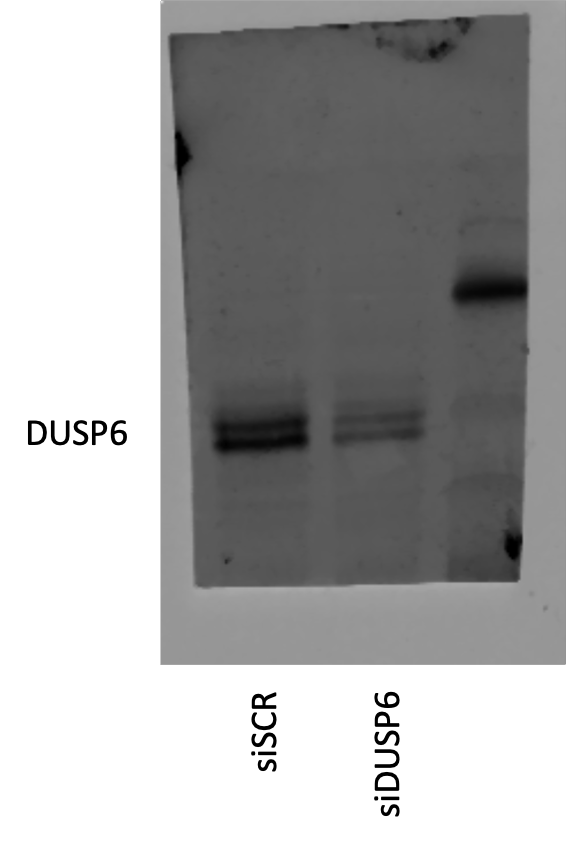

Supplement: Supplementary file 15 — Source data Fig. 6 [file 44321_2024_88_MOESM15_ESM.zip › Figure 6/6H/1st/DUSP6.tiff]

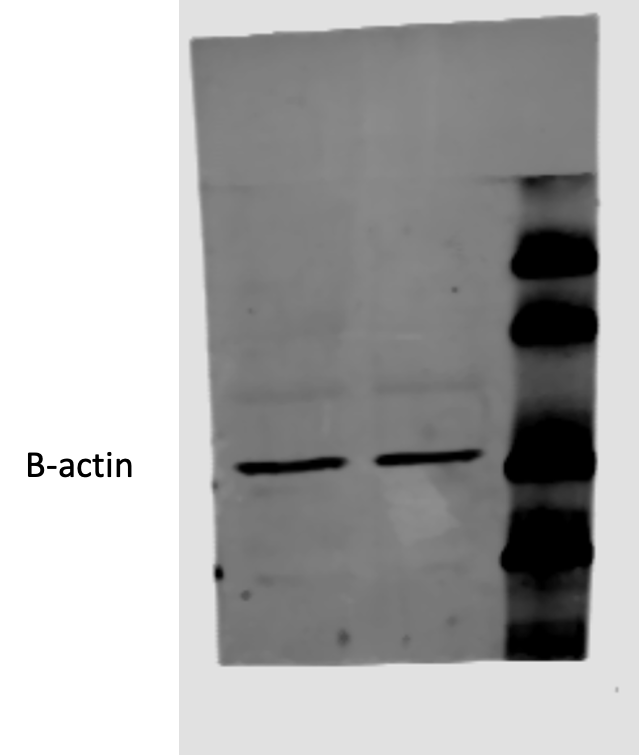

Supplement: Supplementary file 15 — Source data Fig. 6 [file 44321_2024_88_MOESM15_ESM.zip › Figure 6/6H/1st/B-actin.tiff]

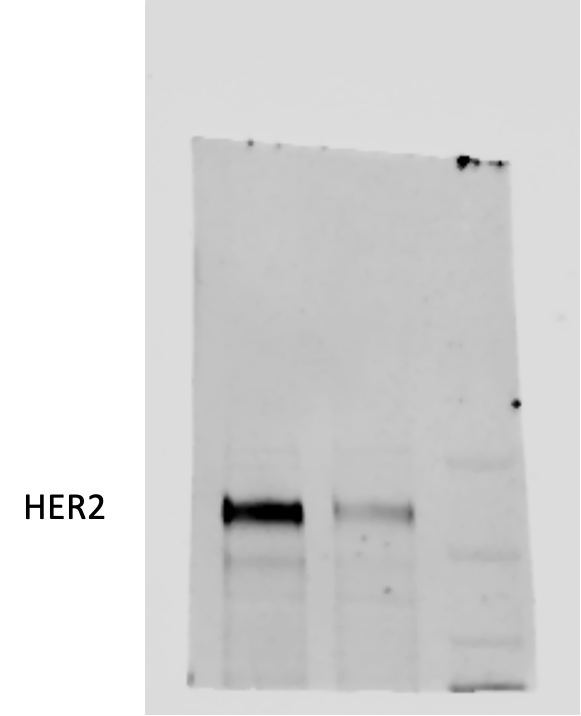

Supplement: Supplementary file 15 — Source data Fig. 6 [file 44321_2024_88_MOESM15_ESM.zip › Figure 6/6H/1st/HER2.tiff]

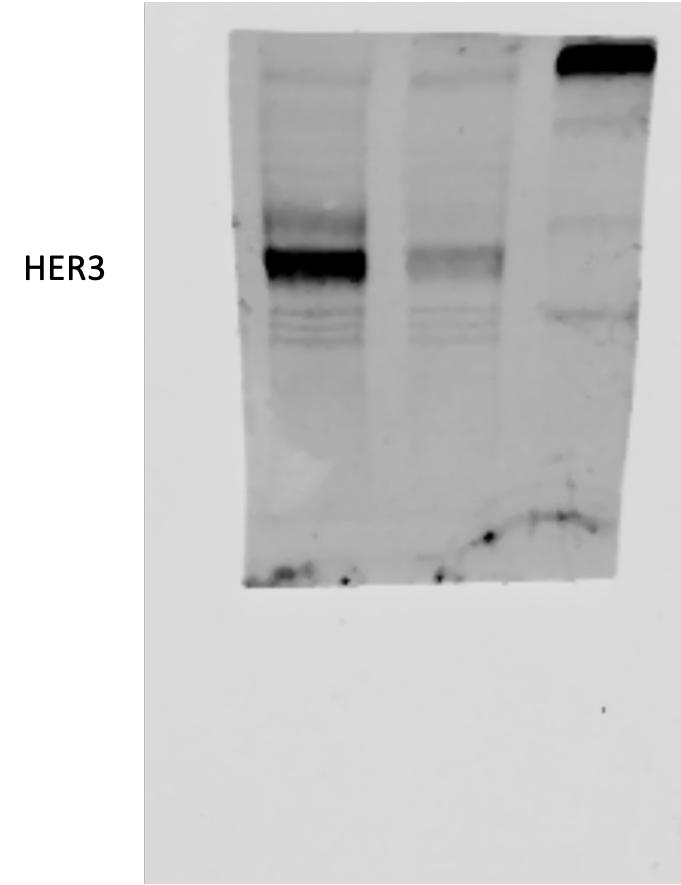

Supplement: Supplementary file 15 — Source data Fig. 6 [file 44321_2024_88_MOESM15_ESM.zip › Figure 6/6H/1st/HER3.tiff]

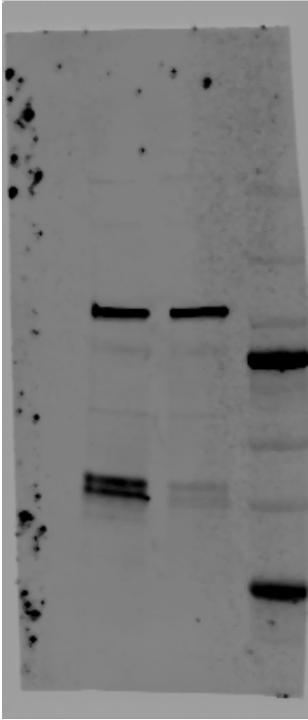

Supplement: Supplementary file 15 — Source data Fig. 6 [file 44321_2024_88_MOESM15_ESM.zip › Figure 6/6H/2nd/DUSP6.tiff]

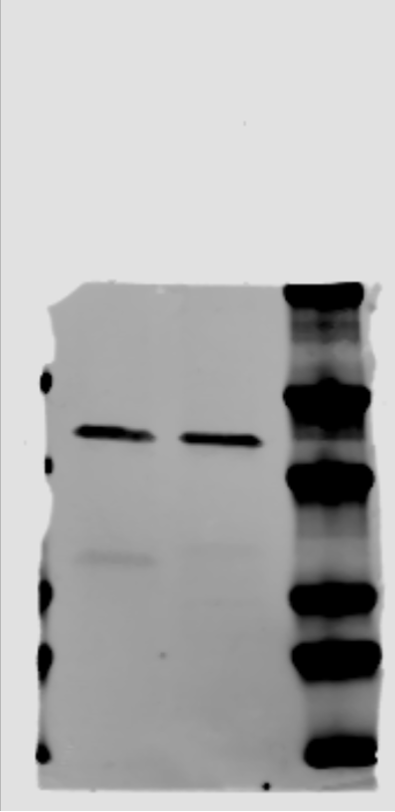

Supplement: Supplementary file 15 — Source data Fig. 6 [file 44321_2024_88_MOESM15_ESM.zip › Figure 6/6H/2nd/B-actin.tiff]

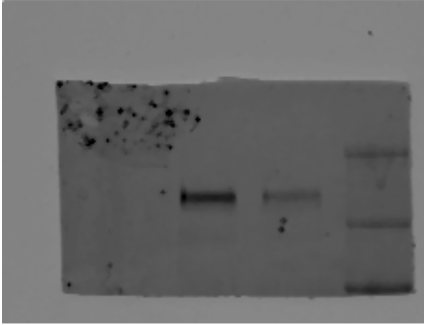

Supplement: Supplementary file 15 — Source data Fig. 6 [file 44321_2024_88_MOESM15_ESM.zip › Figure 6/6H/2nd/HER2.tiff]

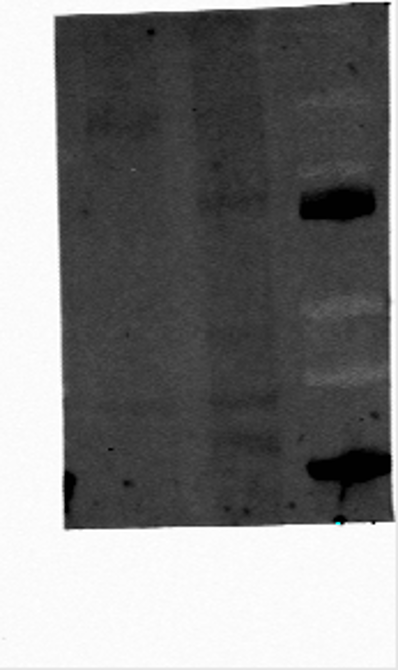

Supplement: Supplementary file 15 — Source data Fig. 6 [file 44321_2024_88_MOESM15_ESM.zip › Figure 6/6H/2nd/HER3.tiff]

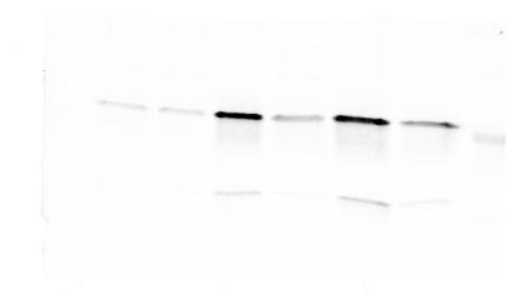

Supplement: Supplementary file 15 — Source data Fig. 6 [file 44321_2024_88_MOESM15_ESM.zip › Figure 6/6C/3rd/Cl-PARP, 25.tiff]

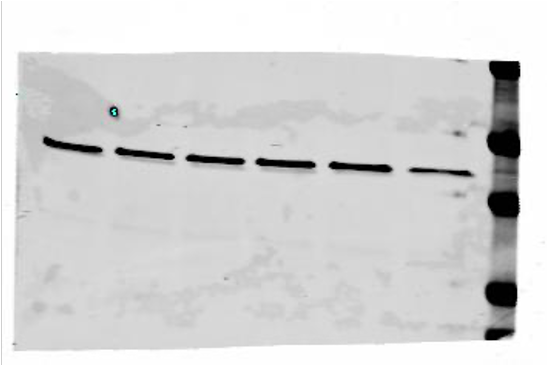

Supplement: Supplementary file 15 — Source data Fig. 6 [file 44321_2024_88_MOESM15_ESM.zip › Figure 6/6C/3rd/B-actin.tiff]

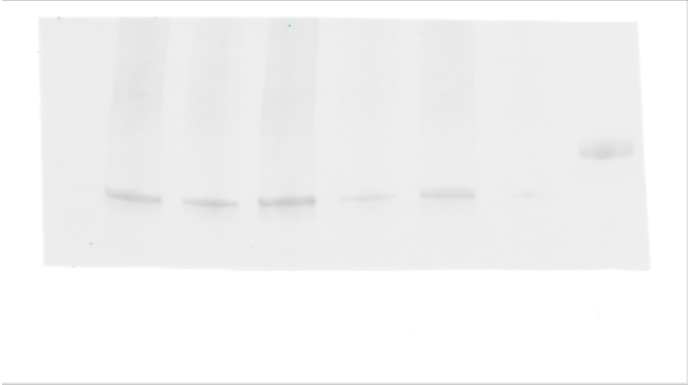

Supplement: Supplementary file 15 — Source data Fig. 6 [file 44321_2024_88_MOESM15_ESM.zip › Figure 6/6C/3rd/p-AKT.tiff]

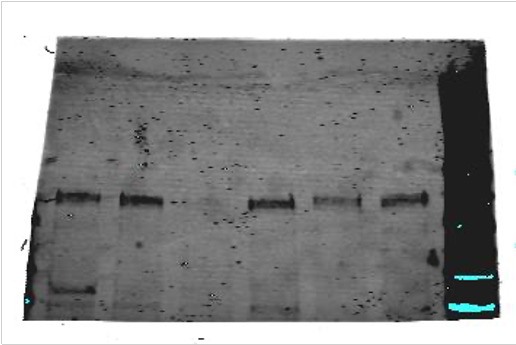

Supplement: Supplementary file 15 — Source data Fig. 6 [file 44321_2024_88_MOESM15_ESM.zip › Figure 6/6C/3rd/HER2.tiff]

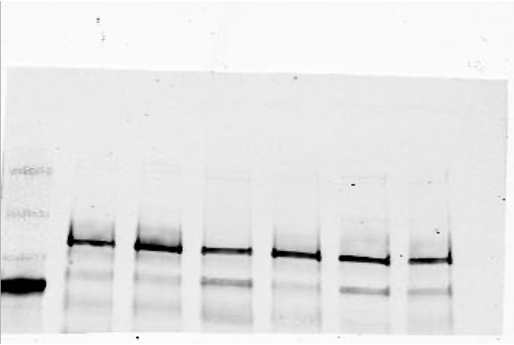

Supplement: Supplementary file 15 — Source data Fig. 6 [file 44321_2024_88_MOESM15_ESM.zip › Figure 6/6C/3rd/Cl-PARP, 89.tiff]

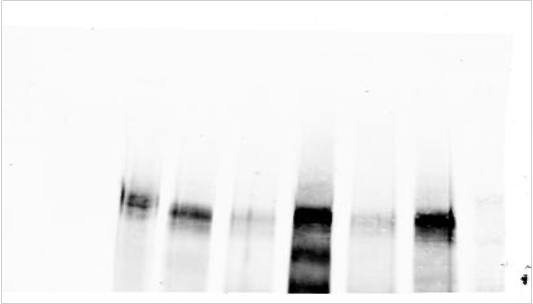

Supplement: Supplementary file 15 — Source data Fig. 6 [file 44321_2024_88_MOESM15_ESM.zip › Figure 6/6C/3rd/HER3.tiff]

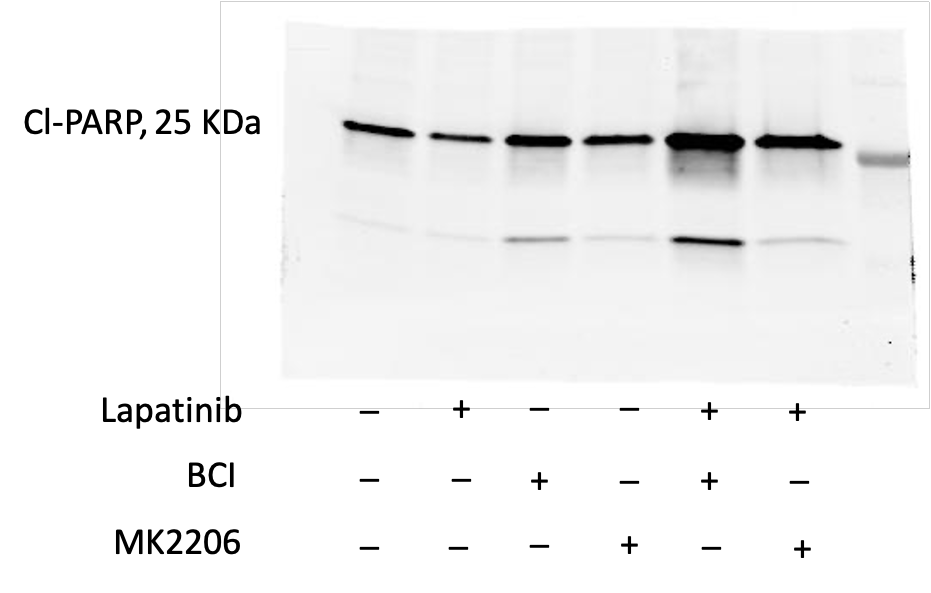

Supplement: Supplementary file 15 — Source data Fig. 6 [file 44321_2024_88_MOESM15_ESM.zip › Figure 6/6C/1st/Cl-PARP, 25.tiff]

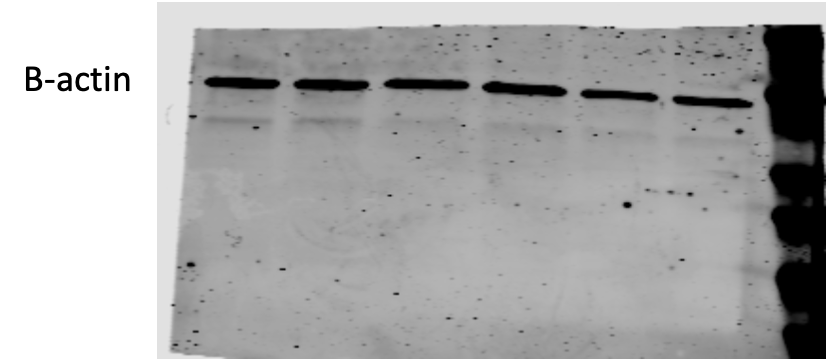

Supplement: Supplementary file 15 — Source data Fig. 6 [file 44321_2024_88_MOESM15_ESM.zip › Figure 6/6C/1st/B-actin.tiff]

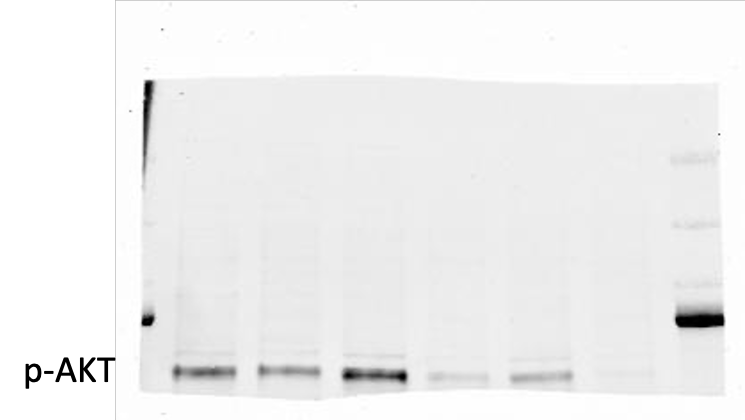

Supplement: Supplementary file 15 — Source data Fig. 6 [file 44321_2024_88_MOESM15_ESM.zip › Figure 6/6C/1st/p-AKT.tiff]

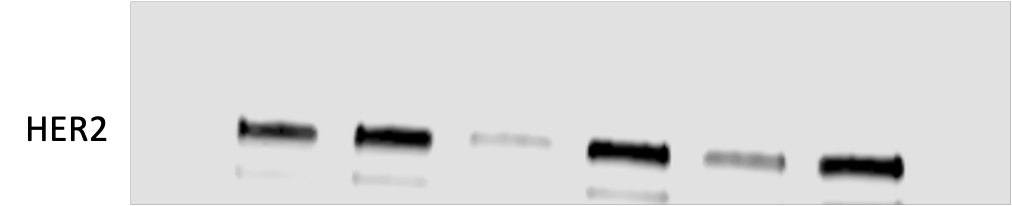

Supplement: Supplementary file 15 — Source data Fig. 6 [file 44321_2024_88_MOESM15_ESM.zip › Figure 6/6C/1st/HER2.tiff]

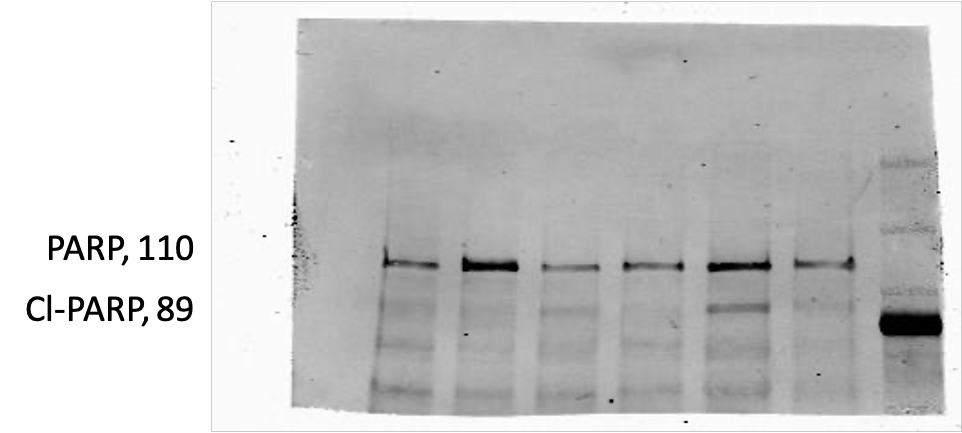

Supplement: Supplementary file 15 — Source data Fig. 6 [file 44321_2024_88_MOESM15_ESM.zip › Figure 6/6C/1st/Cl-PARP, 89.tiff]

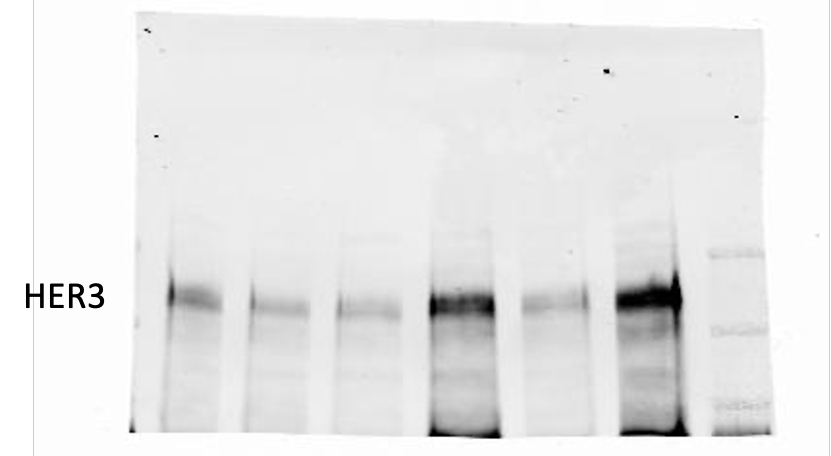

Supplement: Supplementary file 15 — Source data Fig. 6 [file 44321_2024_88_MOESM15_ESM.zip › Figure 6/6C/1st/HER3.tiff]

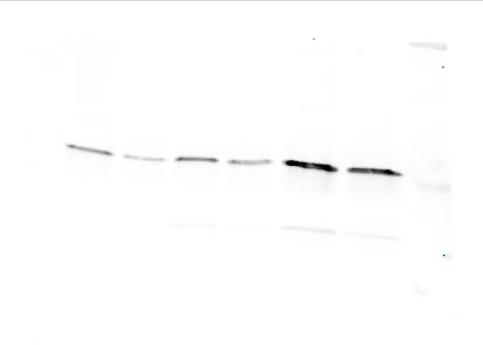

Supplement: Supplementary file 15 — Source data Fig. 6 [file 44321_2024_88_MOESM15_ESM.zip › Figure 6/6C/2nd/Cl-PARP, 25.tiff]

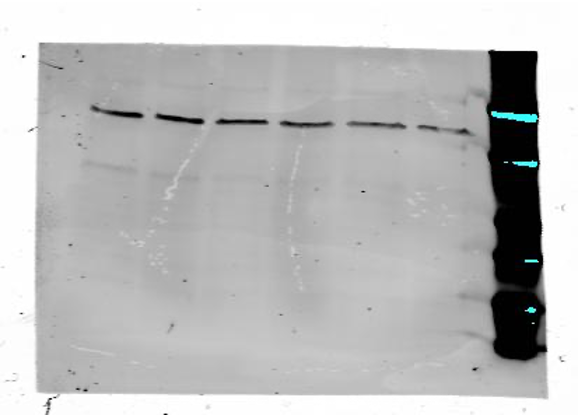

Supplement: Supplementary file 15 — Source data Fig. 6 [file 44321_2024_88_MOESM15_ESM.zip › Figure 6/6C/2nd/B-actin.tiff]

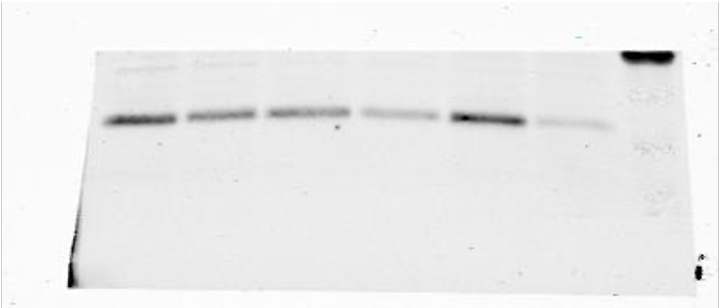

Supplement: Supplementary file 15 — Source data Fig. 6 [file 44321_2024_88_MOESM15_ESM.zip › Figure 6/6C/2nd/p-AKT.tiff]

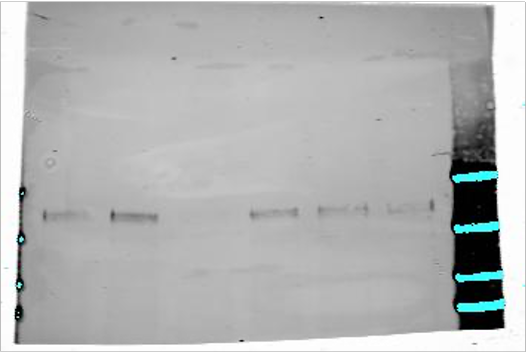

Supplement: Supplementary file 15 — Source data Fig. 6 [file 44321_2024_88_MOESM15_ESM.zip › Figure 6/6C/2nd/HER2.tiff]

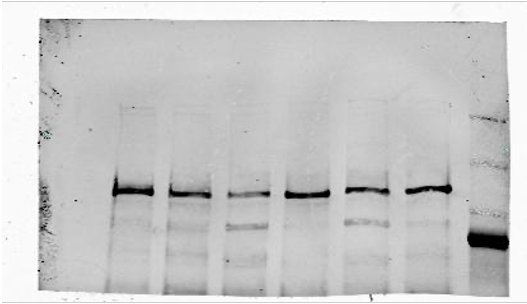

Supplement: Supplementary file 15 — Source data Fig. 6 [file 44321_2024_88_MOESM15_ESM.zip › Figure 6/6C/2nd/Cl-PARP, 89.tiff]

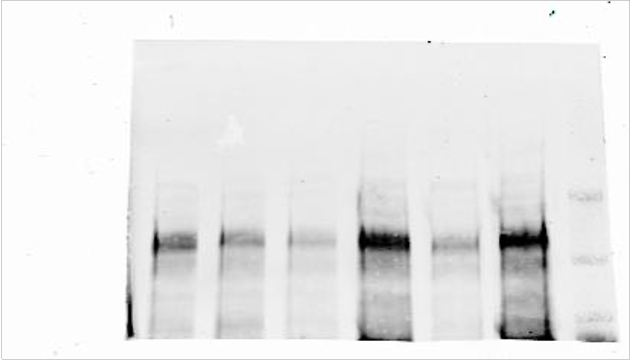

Supplement: Supplementary file 15 — Source data Fig. 6 [file 44321_2024_88_MOESM15_ESM.zip › Figure 6/6C/2nd/HER3.tiff]

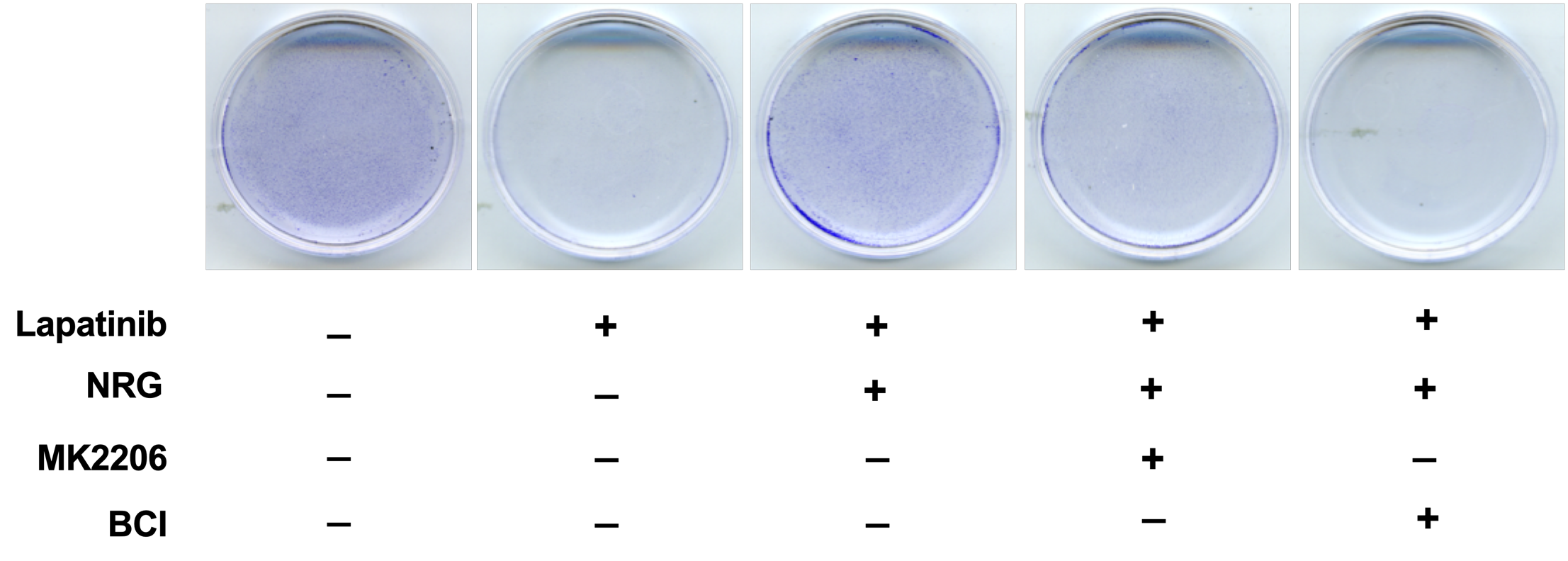

Supplement: Supplementary file 16 — Source data Fig. 7 [file 44321_2024_88_MOESM16_ESM.zip › Figure 7/7D/7D.tiff]

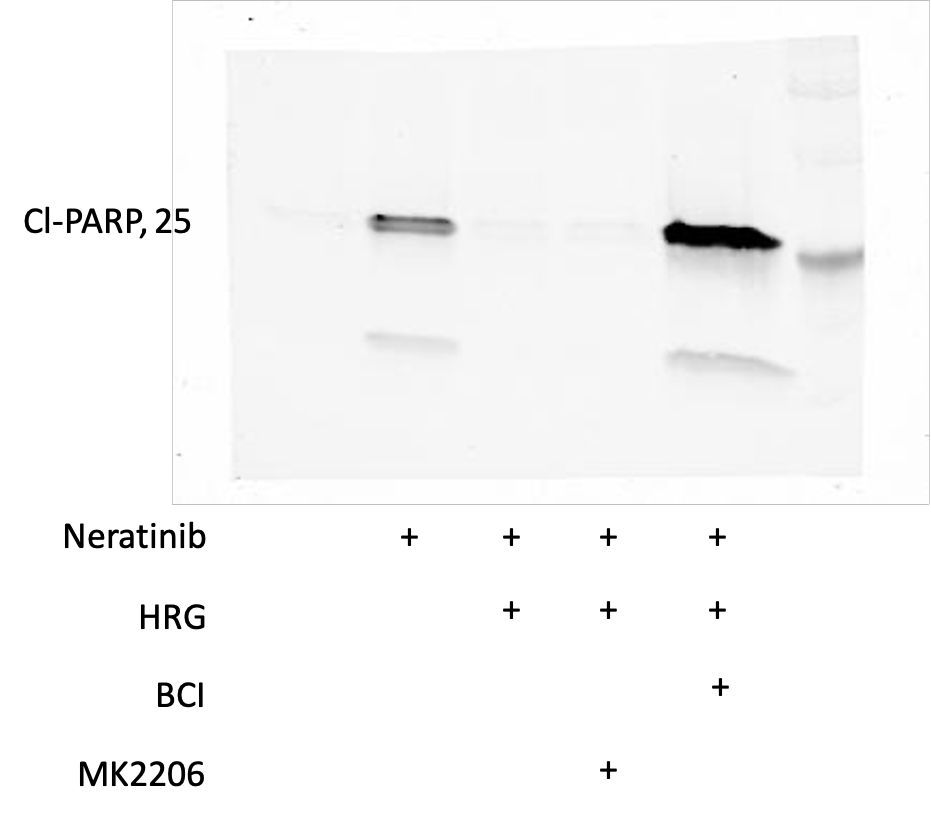

Supplement: Supplementary file 16 — Source data Fig. 7 [file 44321_2024_88_MOESM16_ESM.zip › Figure 7/7B/Cl-PARP, 25.tiff]

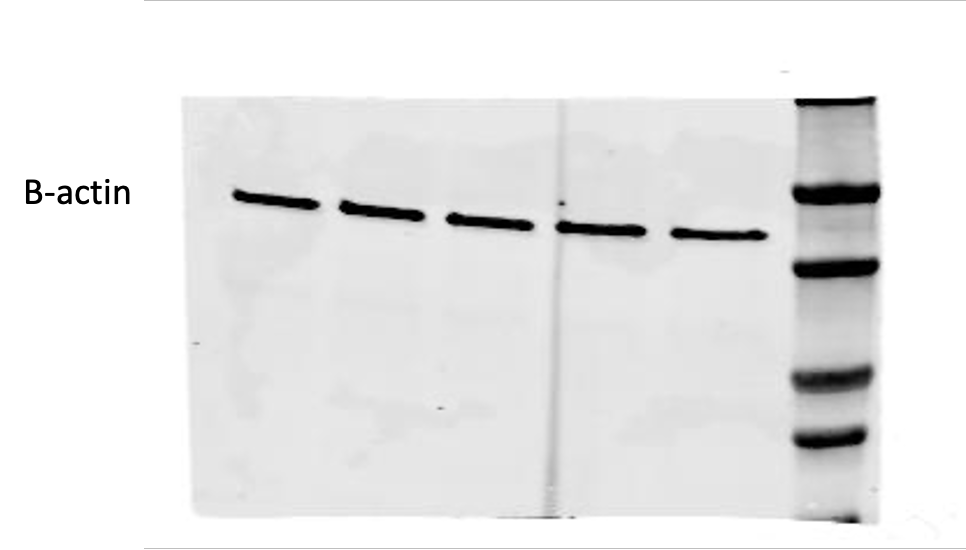

Supplement: Supplementary file 16 — Source data Fig. 7 [file 44321_2024_88_MOESM16_ESM.zip › Figure 7/7B/B-actin.tiff]

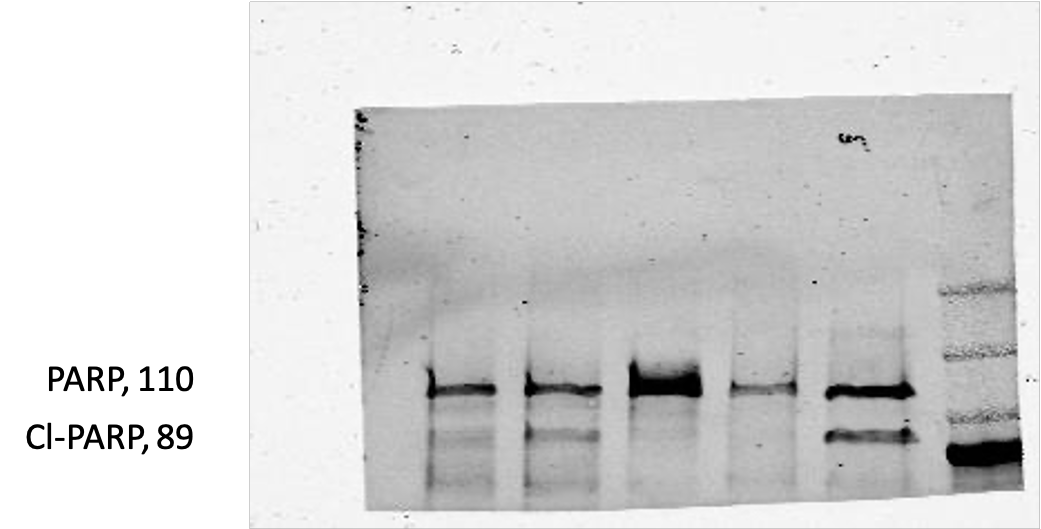

Supplement: Supplementary file 16 — Source data Fig. 7 [file 44321_2024_88_MOESM16_ESM.zip › Figure 7/7B/Cl-PARP, 89.tiff]

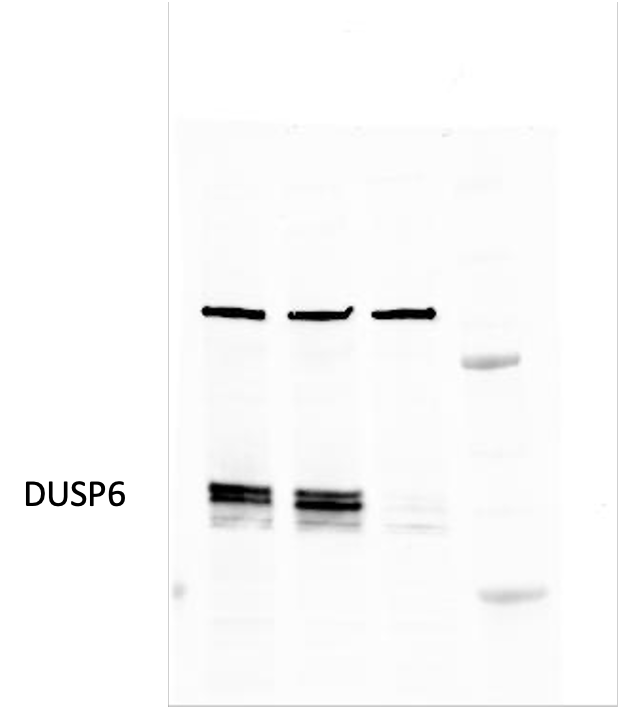

Supplement: Supplementary file 17 — Source data Fig. 8 [file 44321_2024_88_MOESM17_ESM.zip › Figure 8/8G/DUSP6.tiff]

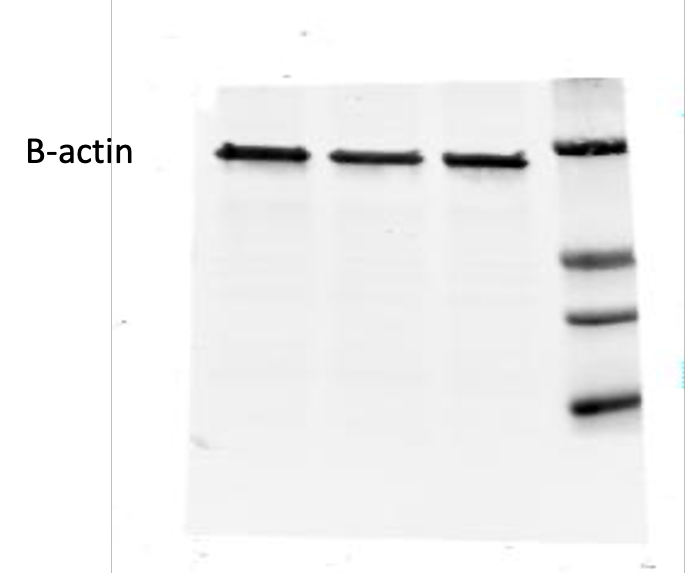

Supplement: Supplementary file 17 — Source data Fig. 8 [file 44321_2024_88_MOESM17_ESM.zip › Figure 8/8G/B-actin.tiff]

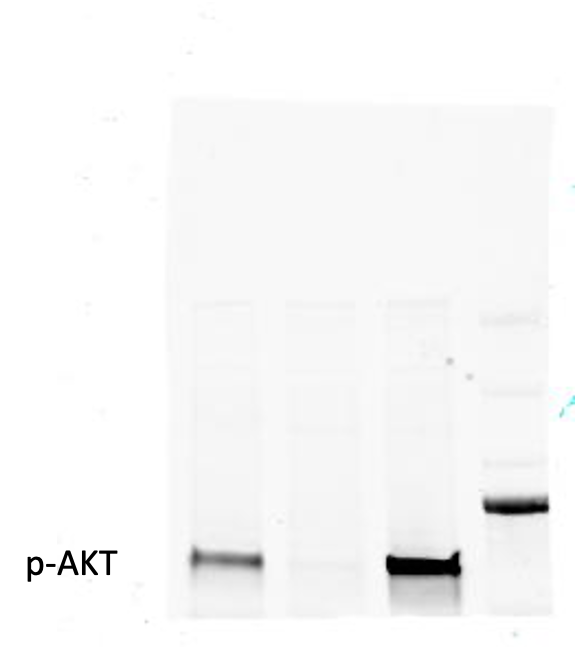

Supplement: Supplementary file 17 — Source data Fig. 8 [file 44321_2024_88_MOESM17_ESM.zip › Figure 8/8G/p-AKT.tiff]

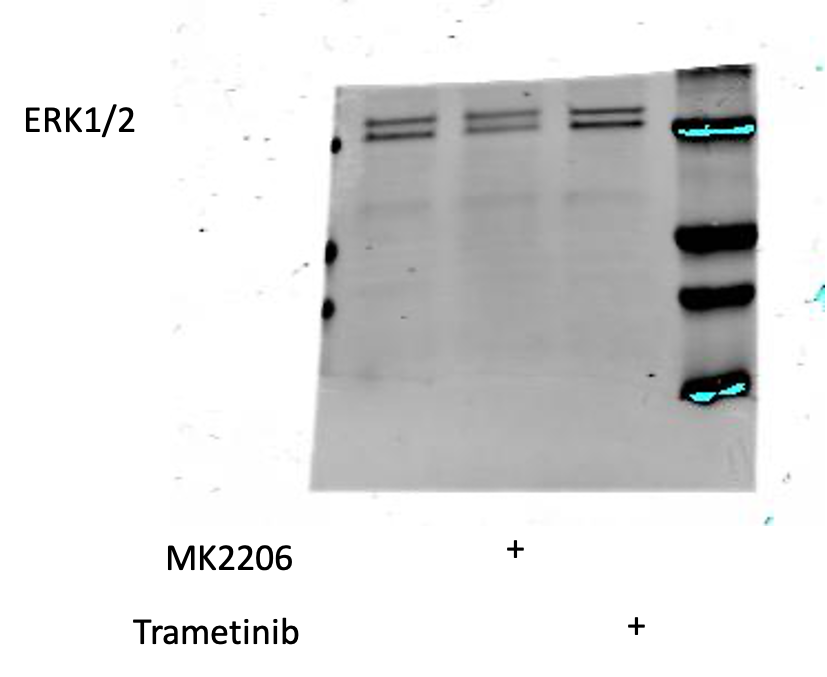

Supplement: Supplementary file 17 — Source data Fig. 8 [file 44321_2024_88_MOESM17_ESM.zip › Figure 8/8G/ERK1:2.tiff]

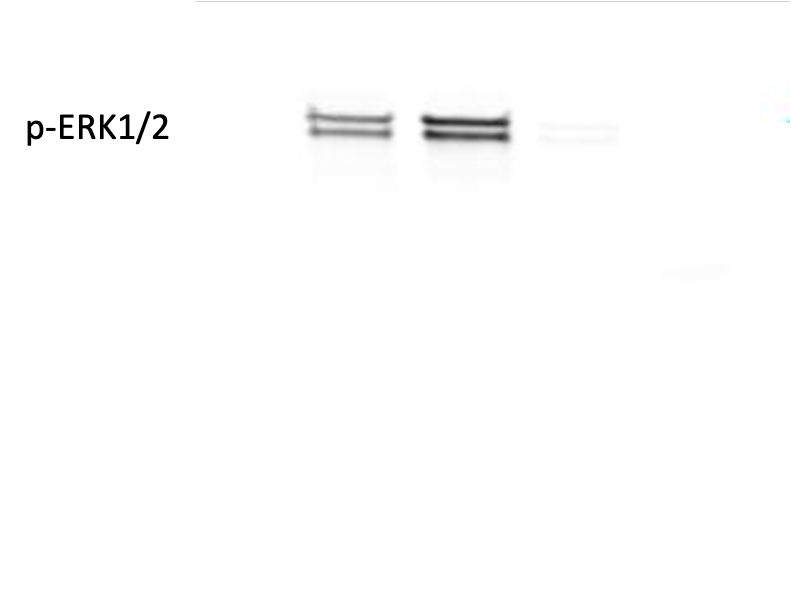

Supplement: Supplementary file 17 — Source data Fig. 8 [file 44321_2024_88_MOESM17_ESM.zip › Figure 8/8G/p-ERK1:2.tiff]

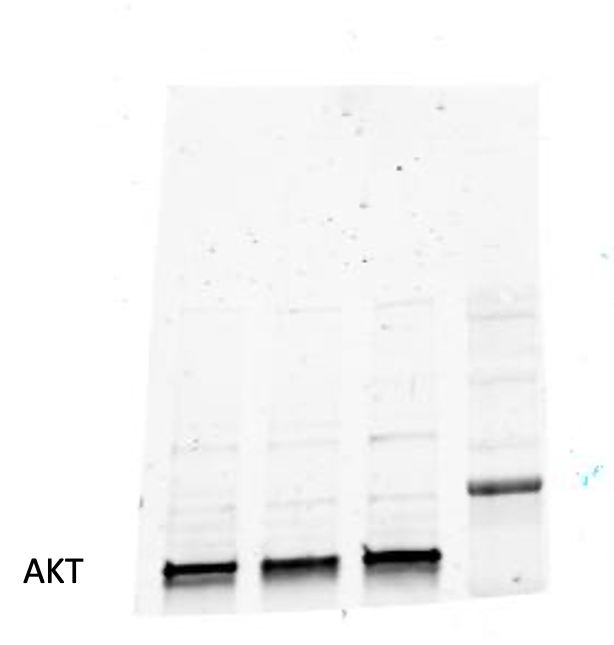

Supplement: Supplementary file 17 — Source data Fig. 8 [file 44321_2024_88_MOESM17_ESM.zip › Figure 8/8G/AKT.tiff]

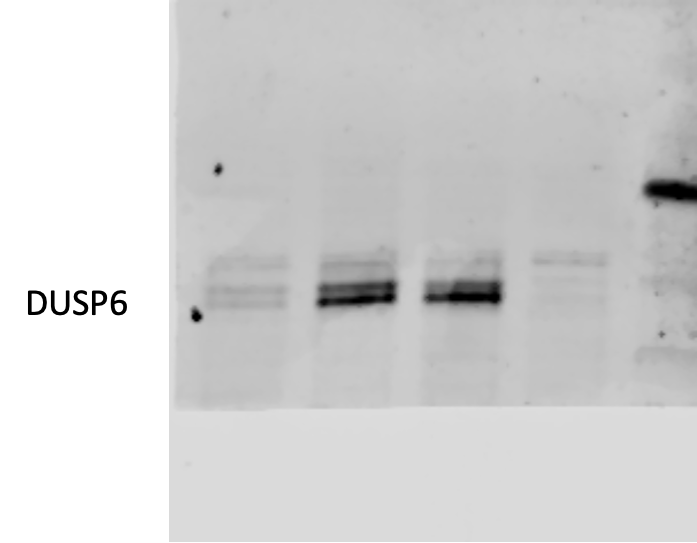

Supplement: Supplementary file 17 — Source data Fig. 8 [file 44321_2024_88_MOESM17_ESM.zip › Figure 8/8F/DUSP6.tiff]

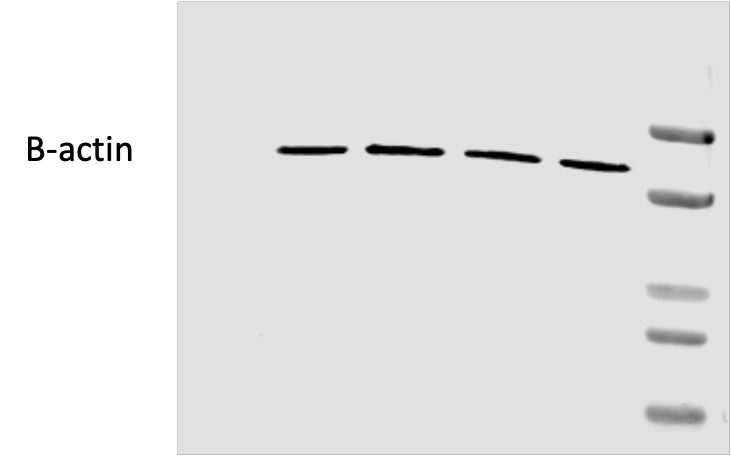

Supplement: Supplementary file 17 — Source data Fig. 8 [file 44321_2024_88_MOESM17_ESM.zip › Figure 8/8F/B-actin.tiff]

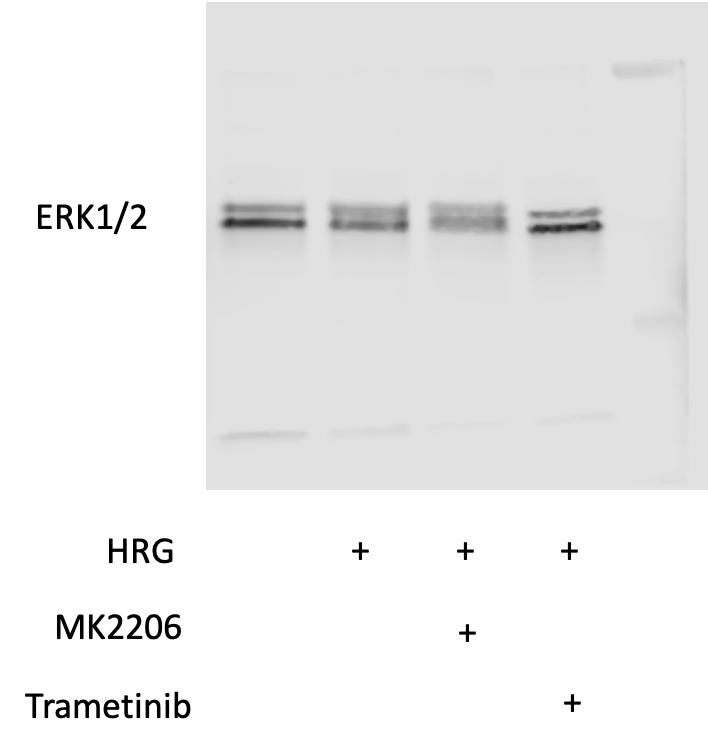

Supplement: Supplementary file 17 — Source data Fig. 8 [file 44321_2024_88_MOESM17_ESM.zip › Figure 8/8F/ERK1:2.tiff]

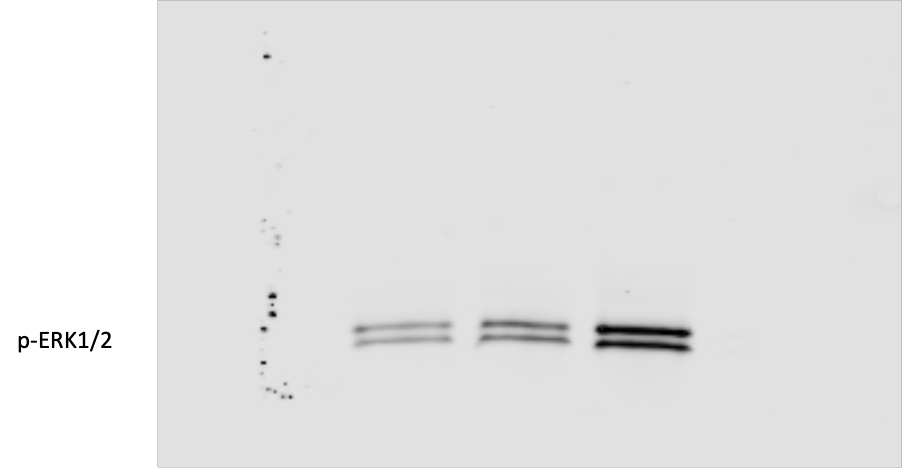

Supplement: Supplementary file 17 — Source data Fig. 8 [file 44321_2024_88_MOESM17_ESM.zip › Figure 8/8F/p-ERK1:2.tiff]

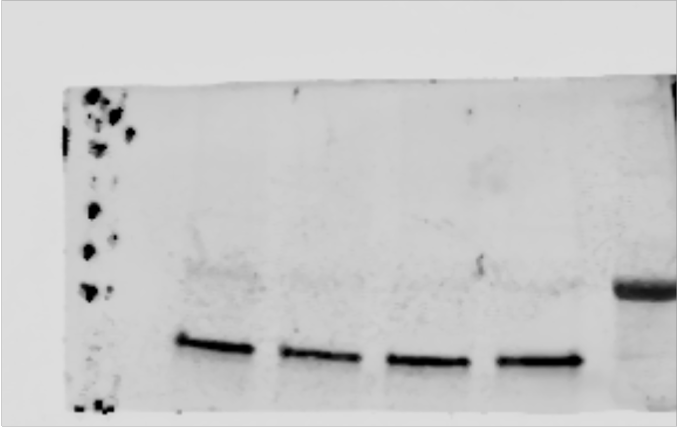

Supplement: Supplementary file 17 — Source data Fig. 8 [file 44321_2024_88_MOESM17_ESM.zip › Figure 8/8F/AKT.tiff]

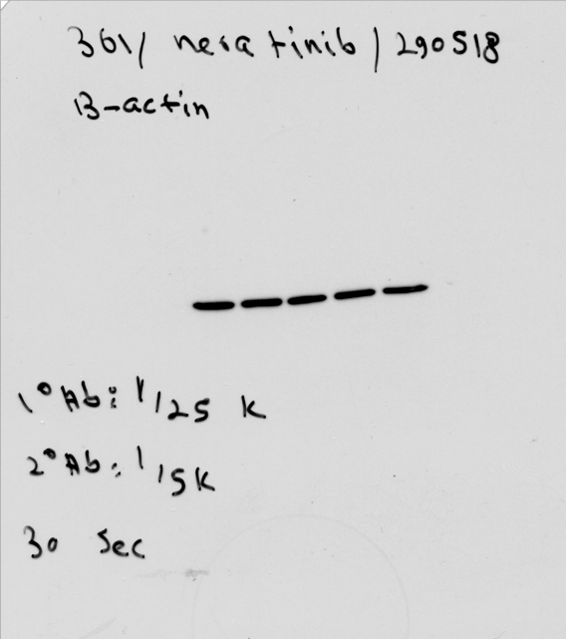

Supplement: Supplementary file 17 — Source data Fig. 8 [file 44321_2024_88_MOESM17_ESM.zip › Figure 8/8C/B-actin.tiff]

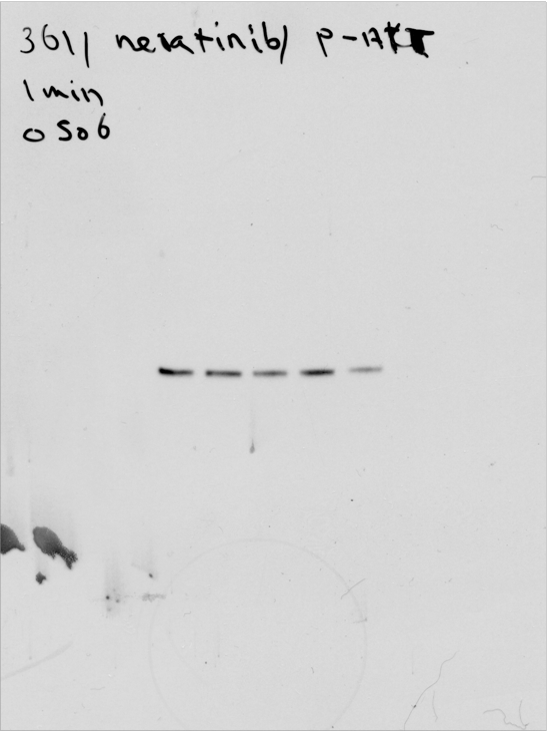

Supplement: Supplementary file 17 — Source data Fig. 8 [file 44321_2024_88_MOESM17_ESM.zip › Figure 8/8C/p-AKT.tiff]

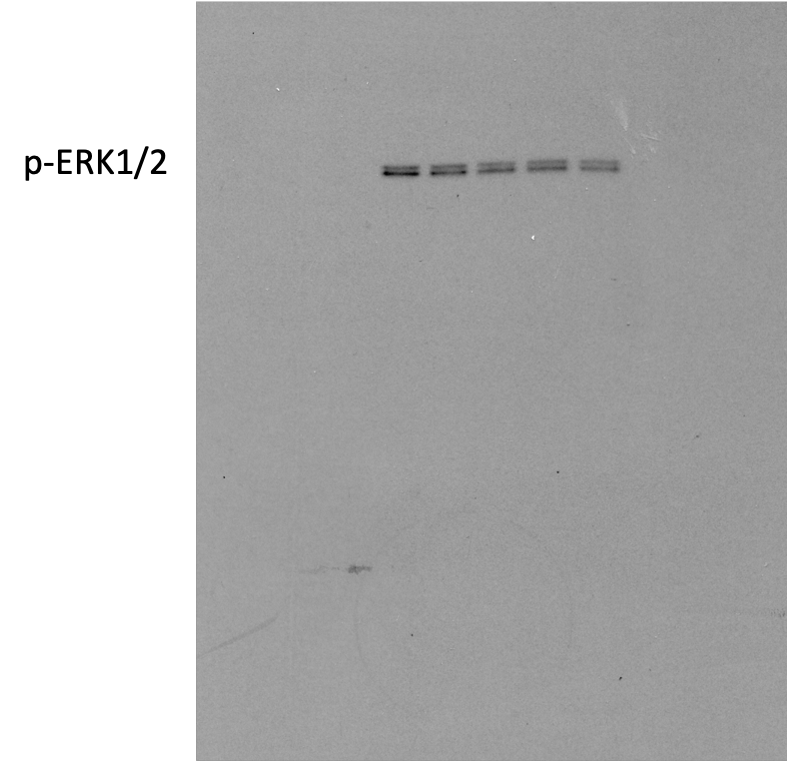

Supplement: Supplementary file 17 — Source data Fig. 8 [file 44321_2024_88_MOESM17_ESM.zip › Figure 8/8C/p-ERK1:2.tiff]

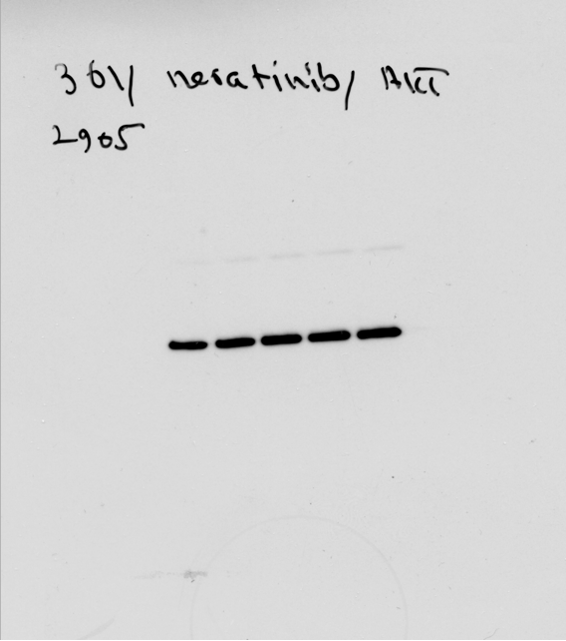

Supplement: Supplementary file 17 — Source data Fig. 8 [file 44321_2024_88_MOESM17_ESM.zip › Figure 8/8C/AKT.tiff]

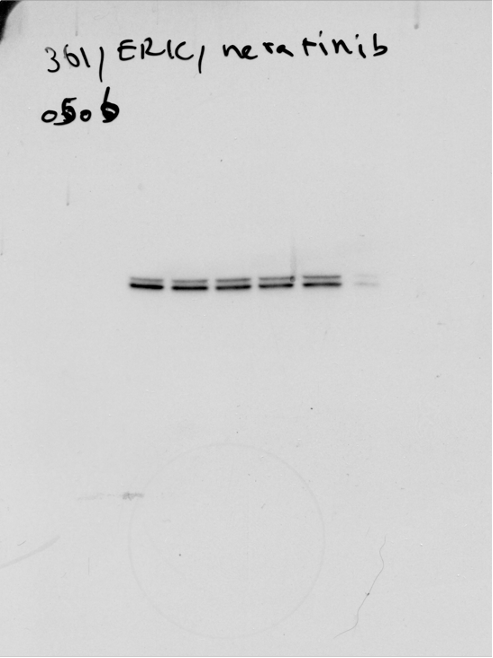

Supplement: Supplementary file 17 — Source data Fig. 8 [file 44321_2024_88_MOESM17_ESM.zip › Figure 8/8C/ERK.tiff]

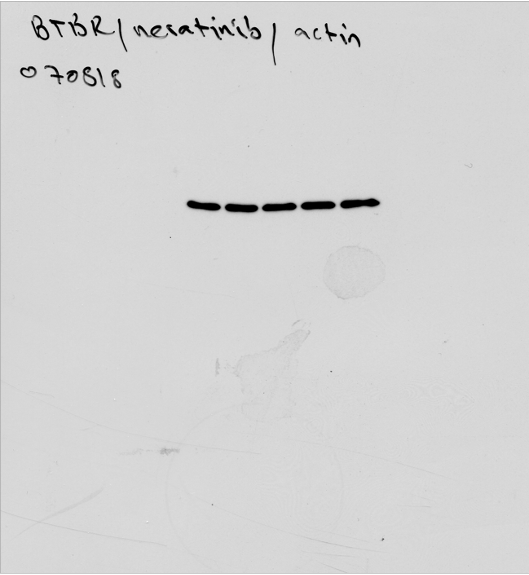

Supplement: Supplementary file 17 — Source data Fig. 8 [file 44321_2024_88_MOESM17_ESM.zip › Figure 8/8B/B-actin.tiff]
