# Supplementary figures and images for: DUSP6 inhibition overcomes neuregulin/HER3-driven therapy tolerance in HER2+ breast cancer (part 2 of 2)
Source: EMBO Mol Med. 2024 Jun 17;16(7):8. doi: 10.1038/s44321-024-00088-0 (PMC11251193; doi:10.1038/s44321-024-00088-0)

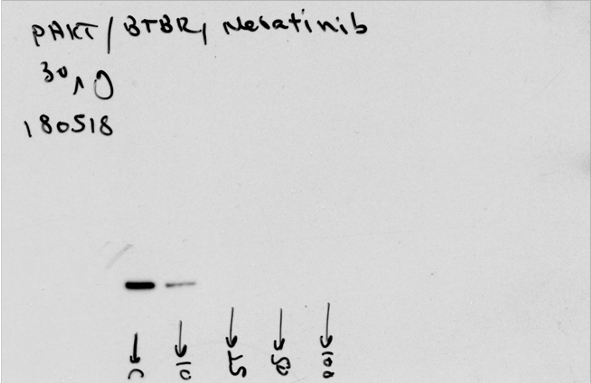

Supplement: Supplementary file 17 — Source data Fig. 8 [file 44321_2024_88_MOESM17_ESM.zip › Figure 8/8B/p-AKT.tiff]

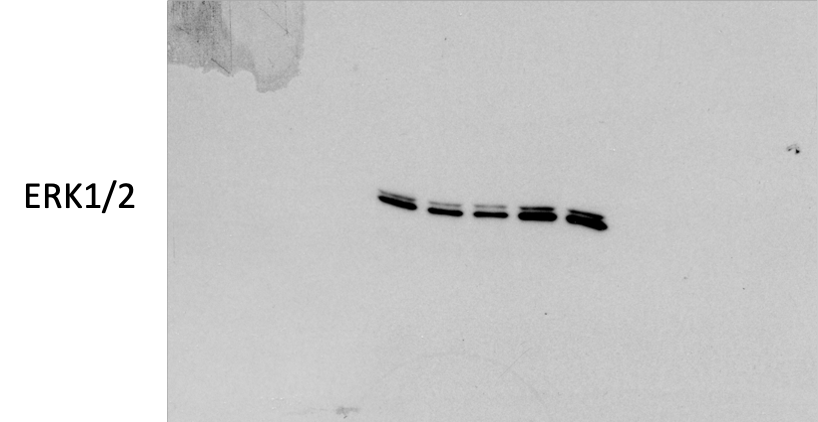

Supplement: Supplementary file 17 — Source data Fig. 8 [file 44321_2024_88_MOESM17_ESM.zip › Figure 8/8B/ERK1:2.tiff]

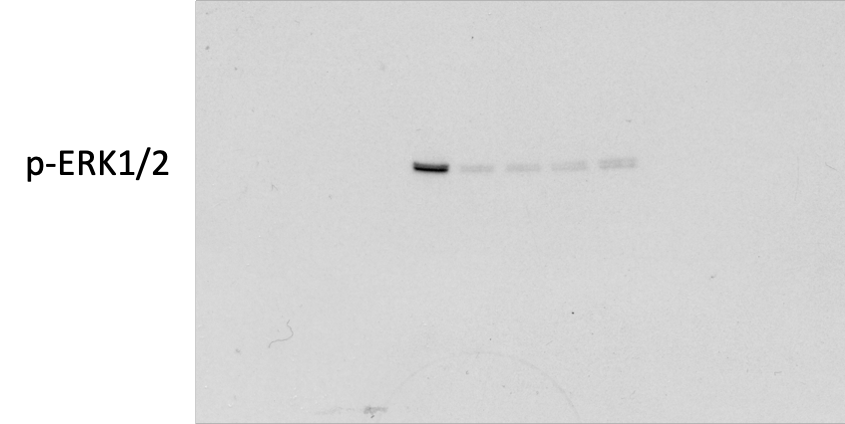

Supplement: Supplementary file 17 — Source data Fig. 8 [file 44321_2024_88_MOESM17_ESM.zip › Figure 8/8B/p-ERK1:2.tiff]

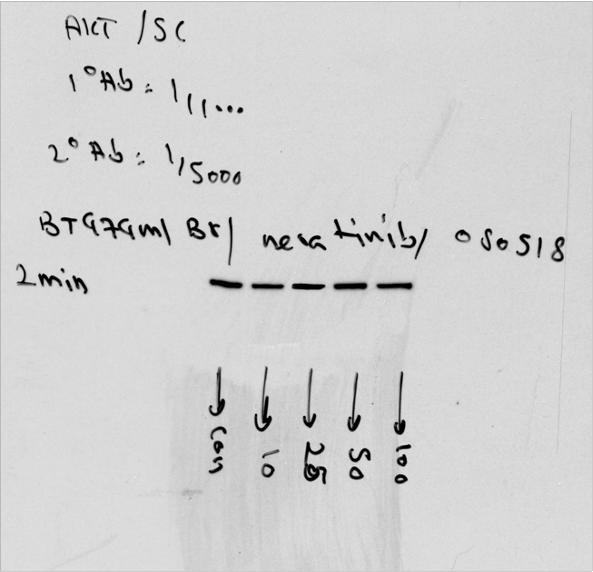

Supplement: Supplementary file 17 — Source data Fig. 8 [file 44321_2024_88_MOESM17_ESM.zip › Figure 8/8B/AKT.tiff]

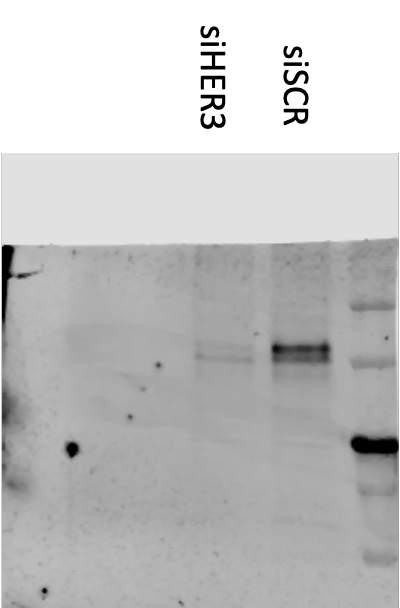

Supplement: Supplementary file 17 — Source data Fig. 8 [file 44321_2024_88_MOESM17_ESM.zip › Figure 8/8D/3rd/DUSP6.tiff]

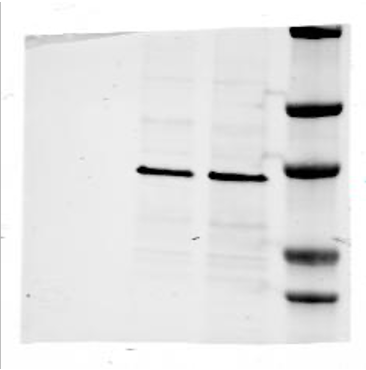

Supplement: Supplementary file 17 — Source data Fig. 8 [file 44321_2024_88_MOESM17_ESM.zip › Figure 8/8D/3rd/B-actin.tiff]

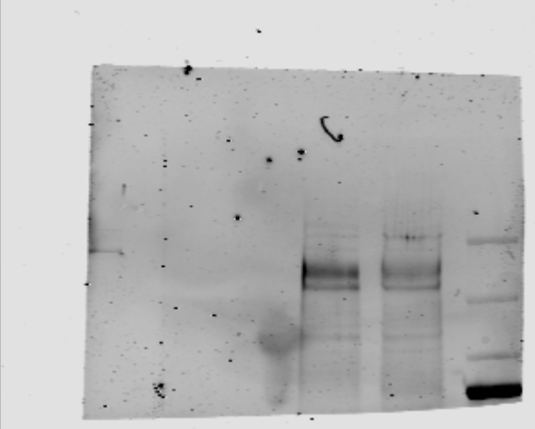

Supplement: Supplementary file 17 — Source data Fig. 8 [file 44321_2024_88_MOESM17_ESM.zip › Figure 8/8D/3rd/HER3.tiff]

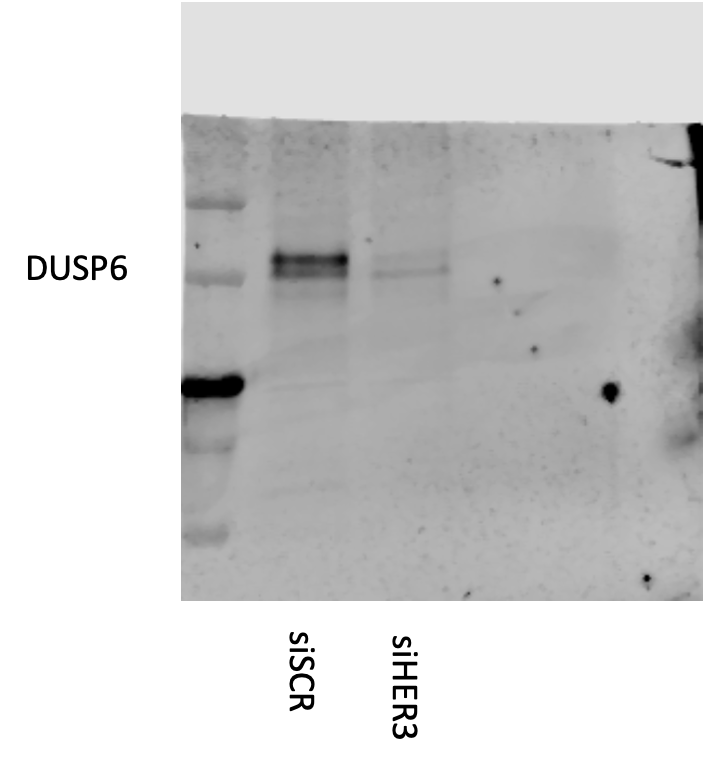

Supplement: Supplementary file 17 — Source data Fig. 8 [file 44321_2024_88_MOESM17_ESM.zip › Figure 8/8D/1st/DUSP6.tiff]

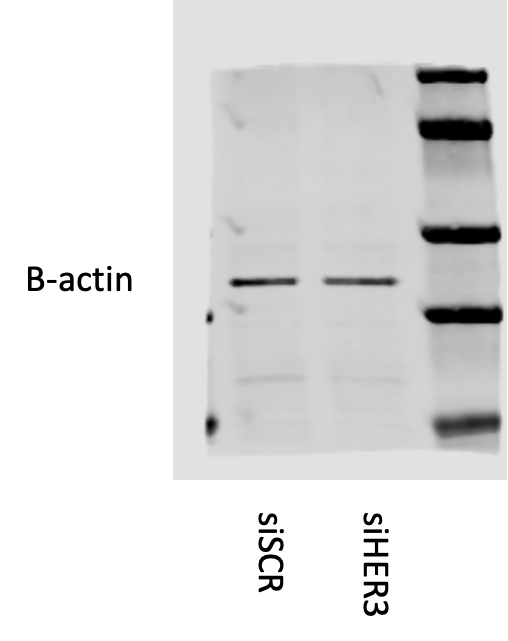

Supplement: Supplementary file 17 — Source data Fig. 8 [file 44321_2024_88_MOESM17_ESM.zip › Figure 8/8D/1st/B-actin.tiff]

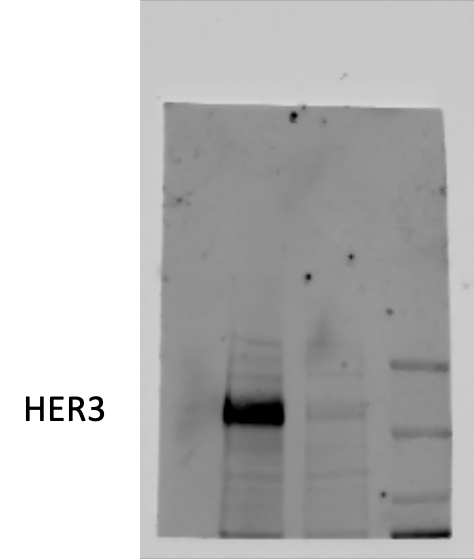

Supplement: Supplementary file 17 — Source data Fig. 8 [file 44321_2024_88_MOESM17_ESM.zip › Figure 8/8D/1st/HER3.tiff]

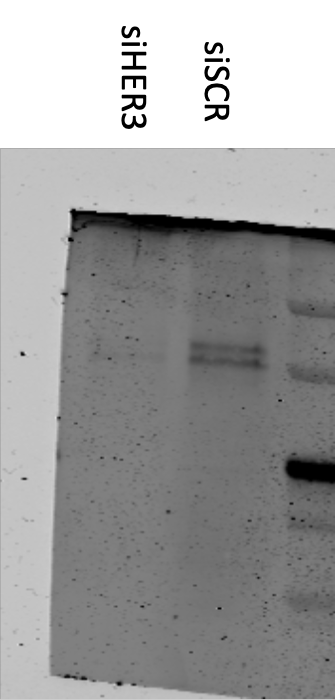

Supplement: Supplementary file 17 — Source data Fig. 8 [file 44321_2024_88_MOESM17_ESM.zip › Figure 8/8D/2nd/DUSP6.tiff]

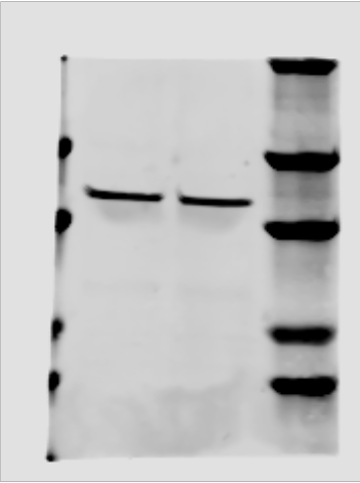

Supplement: Supplementary file 17 — Source data Fig. 8 [file 44321_2024_88_MOESM17_ESM.zip › Figure 8/8D/2nd/B-actin.tiff]

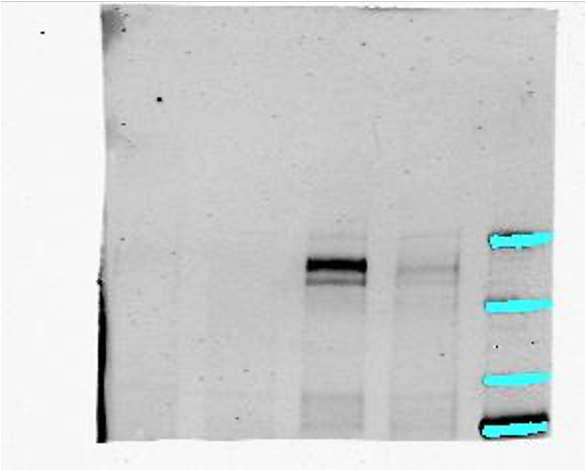

Supplement: Supplementary file 17 — Source data Fig. 8 [file 44321_2024_88_MOESM17_ESM.zip › Figure 8/8D/2nd/HER3.tiff]
