# Supplementary material for: DUSP6 inhibition overcomes neuregulin/HER3-driven therapy tolerance in HER2+ breast cancer
Source: EMBO Mol Med. 2024 Jun 17;16(7):8. doi: 10.1038/s44321-024-00088-0 (PMC11251193; doi:10.1038/s44321-024-00088-0)
Supplement: Supplementary file 18 — Expanded View Figures [file 44321_2024_88_MOESM18_ESM.pdf]

Expanded View Figures

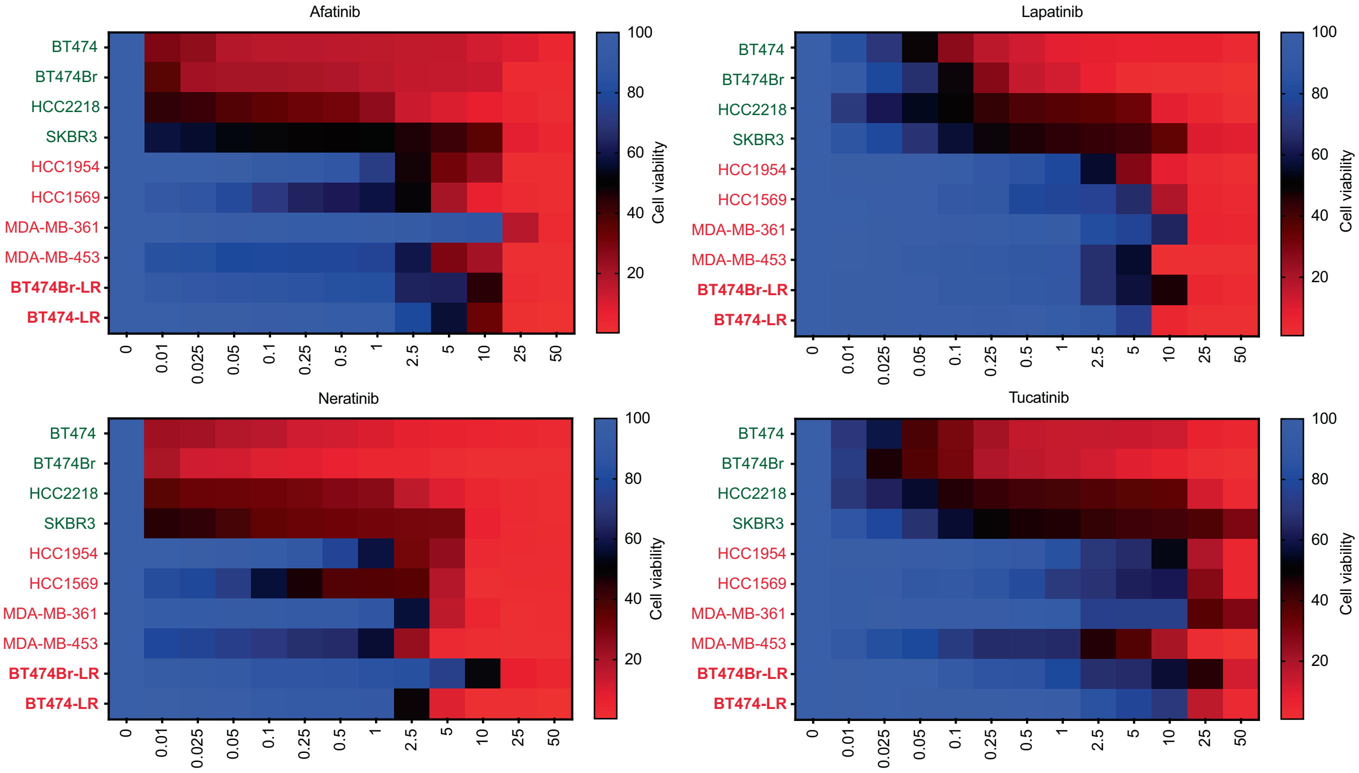

Figure EV1. Profiling of a panel of HER2+ breast cancer cell lines for their sensitivity to HER2 targeting small molecule tyrosine kinase inhibitors.

The primary HER2i sensitive cells are marked on green, and the acquired HER2i resistant cells in red. The long-term resistant (LR) BT-474 and BT-474Br generated by 9-month treatment with lapatinib in this study are denoted in bold. The cells were treated with the increasing concentrations of the indicated HER2i compounds (in μM) for 48 h and cell viability was measured using WST-1 assay.

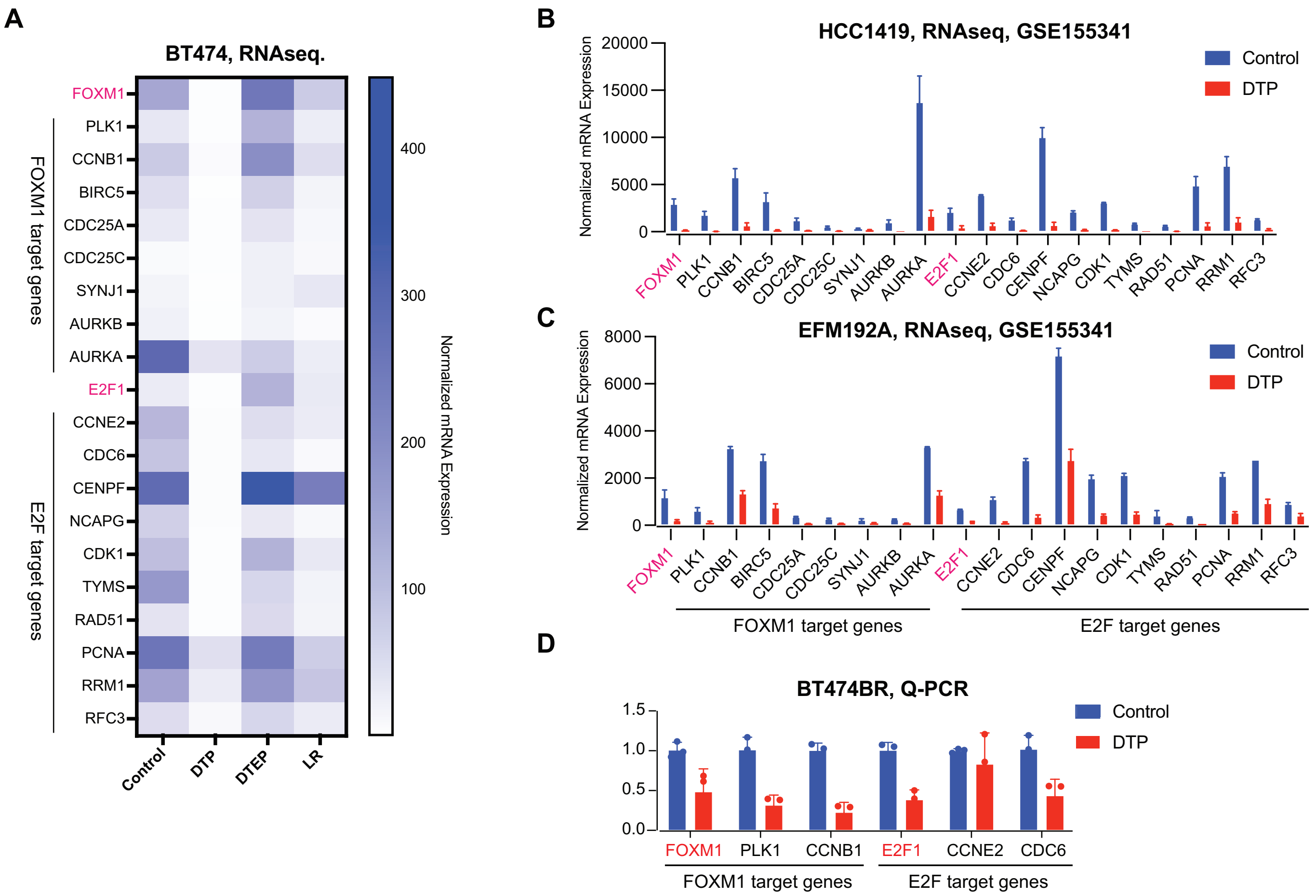

Figure EV2. E2F1 and FOXM1 target genes are inhibited across HER2 + DTP cells.

(**A**) mRNA levels of selected predicted FOXM1 and E2F1 target genes regulated during different steps of acquisition of lapatinib resistance in BT474 cells (Fig. 1). Data is blotted based on RNA sequencing analysis (Dataset EV1). (**B**, **C**) Expression of the predicted FOXM1 and E2F1 target genes in HER2 + HCC1419 and EFM192A cells between the DTP and the control cells. The RNA-seq data was obtained from Dataref: (Chang et al, 2022) (GSE155342). The cells were treated with lapatinib (2.5 μM) for 14 days to reach the DTP state. Data are shown as mean ± SD ($n = 2$). (**D**) Changes in the expression of FOXM1, PLK1, CCNB1, E2F1, CCNE2, and CDC6 in BT474Br cells between the control and the DTP cells treated for 9 days with lapatinib (1 μM). Data is based on Q-PCR analysis from three technical replicates. The analysis was limited to only these genes due to lack of sufficient mRNA material from the strongly growth suppressed DTP cells. Data are shown as mean ± SD ($n = 3$).

© The Author(s)

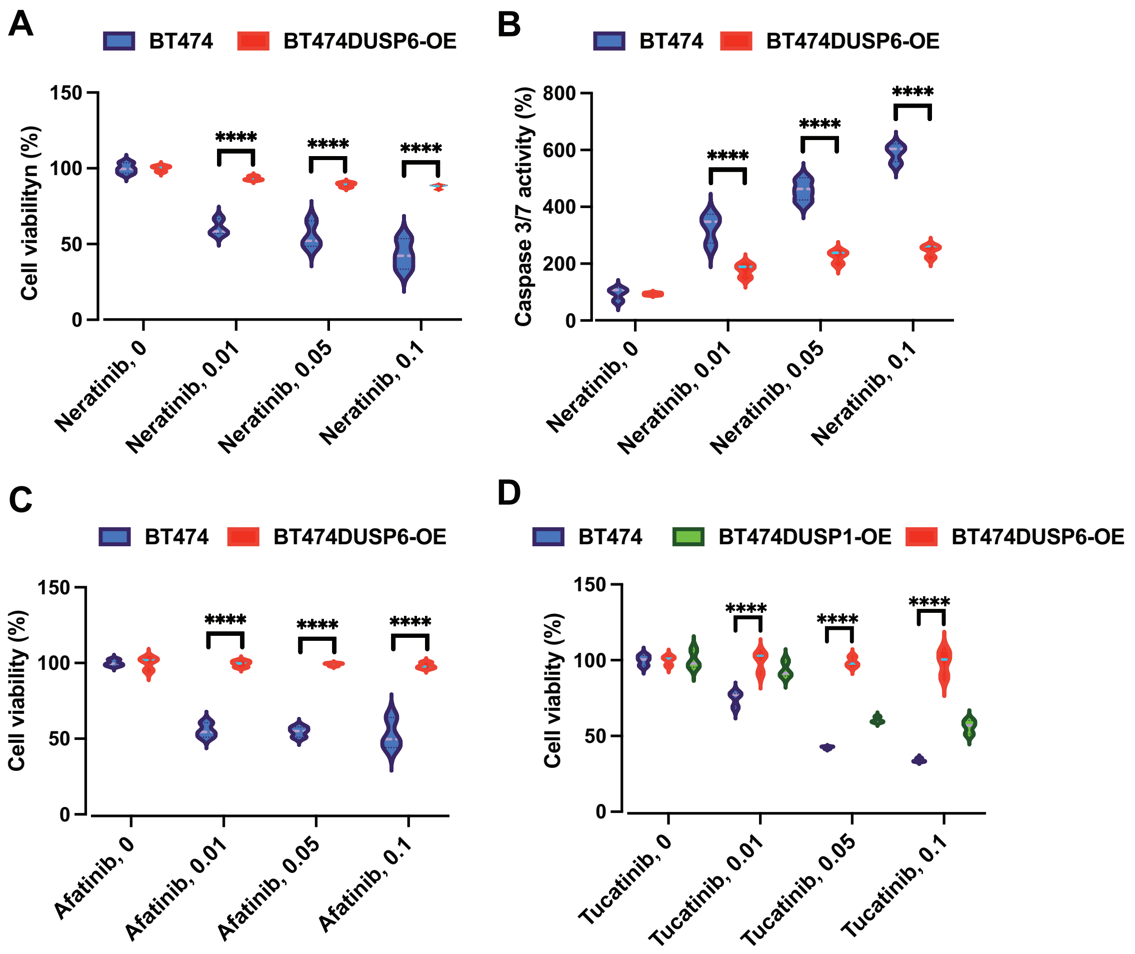

Figure EV3. DUSP6 overexpression protects HER2+ cells from HER2i-induced cell death.

(**A**, **B**) Ectopic overexpression of DUSP6 in BT474 cells inhibits neratinib-elicited effects on cell viability and apoptosis, as measured by WST1 cell viability assay and caspase 3/7 activity, respectively. Data were collected from three independent experiments each performed in triplicate and analyzed by two-way ANOVA followed by Tukey' post hoc test. Statistically significant values of ****$p < 0.0001$ were determined. (**C**) Ectopic overexpression of DUSP6 in BT474 cells inhibits the afatinib-elicited effects on cell viability, as measured by WST1 assay. Data were collected from three independent experiments each performed in triplicate and analyzed by two-way ANOVA followed by Tukey' post hoc test. Statistically significant values of ****$p < 0.0001$ were determined. (**D**) Ectopic overexpression of DUSP6, but not of DUSP1, inhibits the Tucatinib-elicited effects on cell viability, as measured by WST1 assay. Data were collected from three independent experiments each performed in triplicate and analyzed by two-way ANOVA followed by Tukey' post hoc test. Statistically significant values of ****$p < 0.0001$ were determined.

© The Author(s)

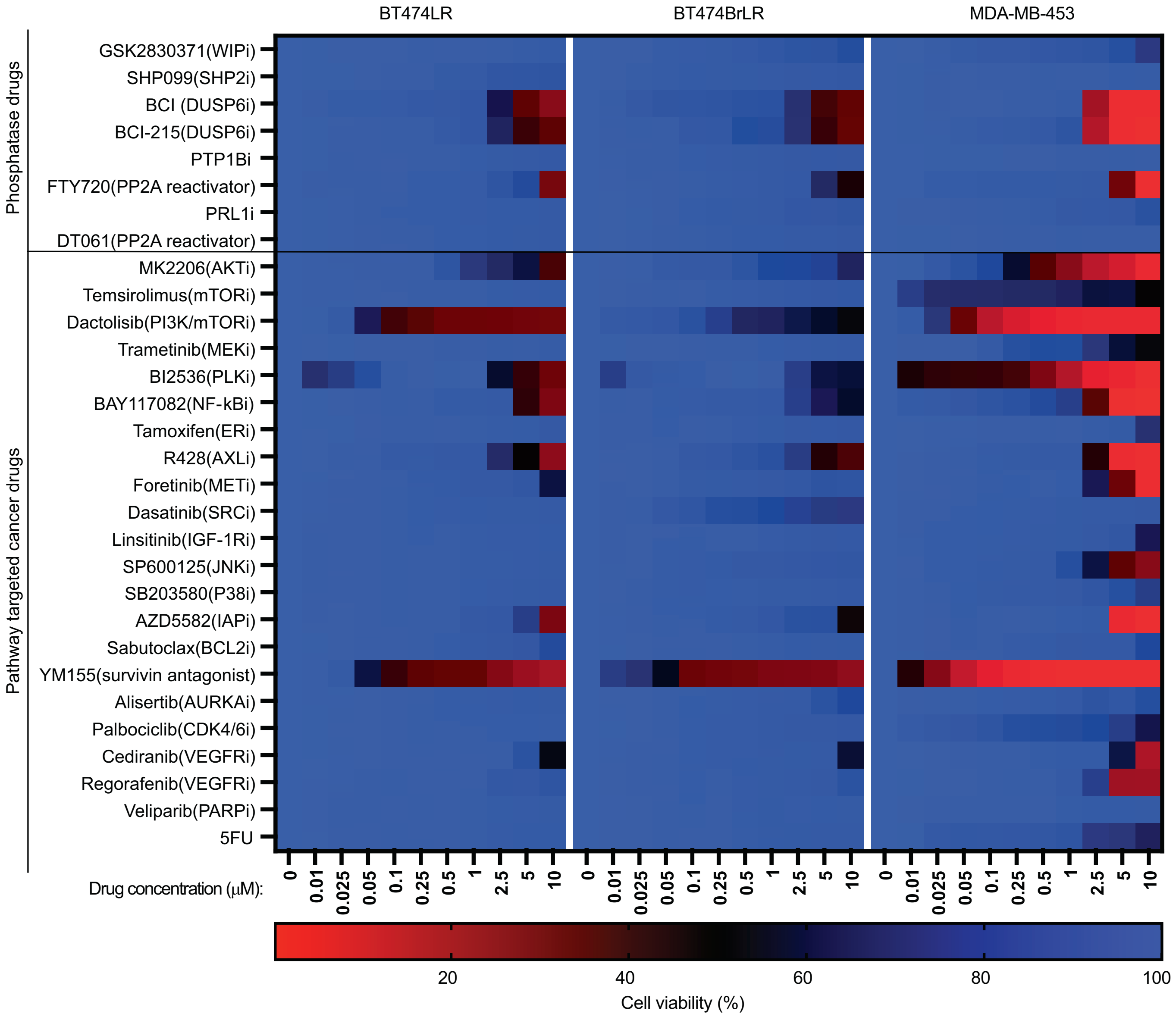

Figure EV4. The anti-proliferative activities of a library of small molecule modulators of phosphatases, kinases, and anti-apoptotic proteins in HER2i-resistant cells.

The indicated cells were treated with the increasing concentrations (in μM) of the compounds for 48 h and cell viability was measured using WST-1 assay. The long-term resistant (LR) BT474 and BT474Br generated de novo by 9-month treatment with lapatinib in this study had comparable drug sensitivity profile to acquired resistant cell line MDA-MB-453. The primary target of the used compound is indicated in parenthesis.

 © The Author(s)

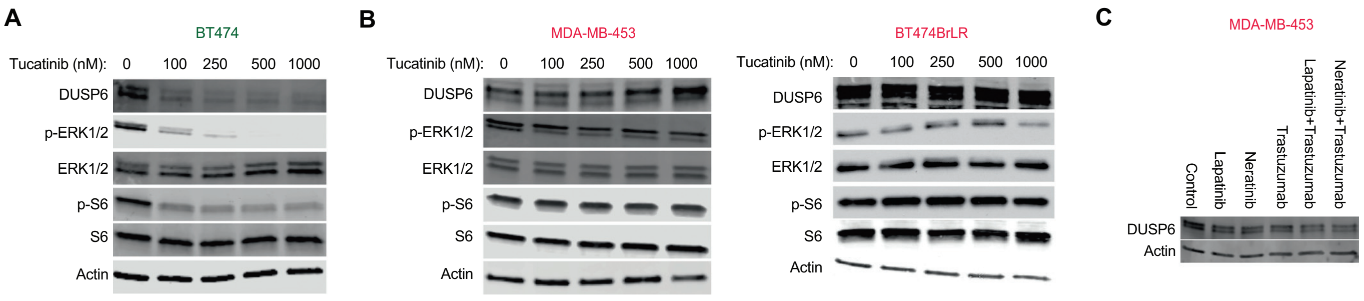

Figure EV5. Lack of DUSP6 inhibition is associated with HER2i resistance.

(**A**, **B**) The effects of tucatinib on DUSP6 expression and the signaling pathway activities in (**A**) HER2i sensitive (green) BT474 cells or (**B**) HER2i resistant (red) MDA-MB-453 and BT474BrLR cells. The cells were treated with increasing concentrations of tucatinib for 48 h, followed by Western blot analysis. (**C**) DUSP6 expression in MDA-MB-453 cells is resistant to multiple HER2is including antibody therapy with trastuzumab. The cells were treated with indicated drugs for 48 h, followed by Western blot analysis.
